# Discovery of Novel 2‑Morpholine Tetrahydroisoquinoline CXCR4 Antagonists with Unique Properties

**DOI:** 10.1021/acs.jmedchem.5c03471

**Published:** 2026-04-06

**Authors:** Yesim Altas Tahirovic, Zafer Sahin, Edgars Jecs, Eric J. Miller, Huy H. Nguyen, Robert J. Wilson, Michelle Kim, Marshall Fritz, Yusr Zaghlula, Savita K. Sharma, Perry Bartsch, Tao Wang, Chi S. Sum, Mary E. Cvijic, Anthony A. Paiva, Gretchen M. Schroeder, Lawrence J. Wilson, Dennis C. Liotta

**Affiliations:** † Department of Chemistry, 1371 Emory University, 1515 Dickey Drive NE, Atlanta, Georgia 30322, United States; ‡ 111059 Bristol-Myers Squibb Research & Development, Princeton, New Jersey 08543, United States

## Abstract

The exploration of tetrahydroisoquinoline-based CXCR4 antagonists as therapeutics is described. Starting from TIQ-15, halogen and heterocycle derivatives led to the identification of the 2-position N-morpholine. The initial compounds, **28** and **42**, had significant improvements in CYP 2D6 inhibition and PAMPA permeability while maintaining CXCR4 potency. These were evaluated in a mouse pharmacokinetic (PK) study, exhibiting low oral bioavailability. In a second round of medicinal chemistry, the *N*-methyl derivative **45** provided surprisingly good CXCR4 potency. The compound had high permeability, modest metabolic stability, and CYP 2D6 inhibition, with a high hERG therapeutic index (TI) and improved exposure when dosed orally to mice. Subsequently, extensive changes to the morpholine ring of **45** led to compounds **75** and **81**, which provided potent CXCR4 activity, high permeability, good selectivity against CYP 2D6, and a high hERG TI. Pharmacokinetic studies in mice for **75** and **81** showed similar improvements to **45** in exposure after oral dosing.

## Graphical abstract

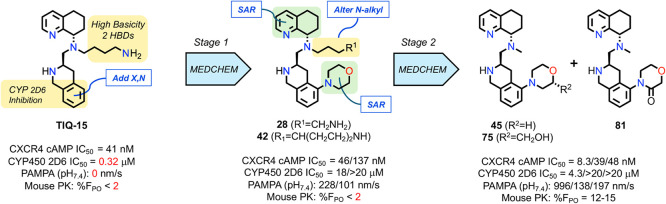

## Introduction

The chemokine CXCR4 has been the center of intense study recently due to the central role it plays in immune system regulation and has been implicated in the pathology of many human diseases.^1–12^ Specifically, this C-X-C type 4 receptor is a seven transmembrane G-protein-coupled receptor of the rhodopsin-like GPCR family and responds to its endogenous ligand CXCL12 (Stromal Cell-Derived Factor-1, SDF-1).^13,14^ Initially, the CXCR4 receptor was discovered by identification, as a coreceptor needed for HIV entry and infection.^15^ Further research has shown that this ligand–receptor system plays a critical role in several important biological processes in healthy tissues, such as hematopoietic stem cell (HSC) quiescence and homing to the bone marrow, chemotaxis, cell survival, and proliferation.^16–19^ The CXCR4 receptor is expressed on many cell types, including hematopoietic stem cells,^20^ leukocytes,^21^ endothelial cells,^22^ and tumor cells.^23^ CXCR4 is also expressed in over 45 different types of cancer, including various types of cancers like leukemia, ovarian, breast, lung, colorectal, and prostate.^1,24^

One of our main interests lies in the critical role the CXCR4/CXCL12 axis plays in cancer and in the regulation of primary tumor growth and metastasis (Figure 1).^9,25^ Tumor-associated stromal cells constitutively express CXCL12.^26^ This paracrine signaling stimulates the proliferation and survival of CXCR4-positive tumor cells (gray). Moreover, CXCR4-expressing tumor cells migrate along the CXCL12 gradient to distant organs showing peak levels of CXCL12 expression, eventually leading to metastases. Tumor cells utilize CXCR4 to access the CXCL12-rich bone marrow microenvironment that favors their growth and survival. High levels of CXCL12 secretion by bone marrow stromal cells are essential for the homing of CXCR4-expressing tumor cells. CXCR4 antagonists can inhibit the crosstalk between tumor and stromal cells and mobilize cancer cells from this protective microenvironment, making them more sensitive to conventional drugs (standard chemotherapy).^3,27,28^ High expression of CXCL12 by tumor cells and tumor-associated stromal cells forms a local gradient of the chemokine in the tumor region. CXCR4-expressing bone marrow-derived progenitor cells are thus recruited to the tumor, where they contribute to the process of angiogenesis by supporting newly formed blood vessels and by releasing other pro-angiogenic factors (Figure 1).^24,29–31^ Furthermore, CXCR4 is also involved in tumor immunosuppression by the recruitment of FOX3P^+^ Treg cells, which block the migration and action of cancer cell-killing CD8 T-lymphocytes.^32–35^

**Figure 1:**
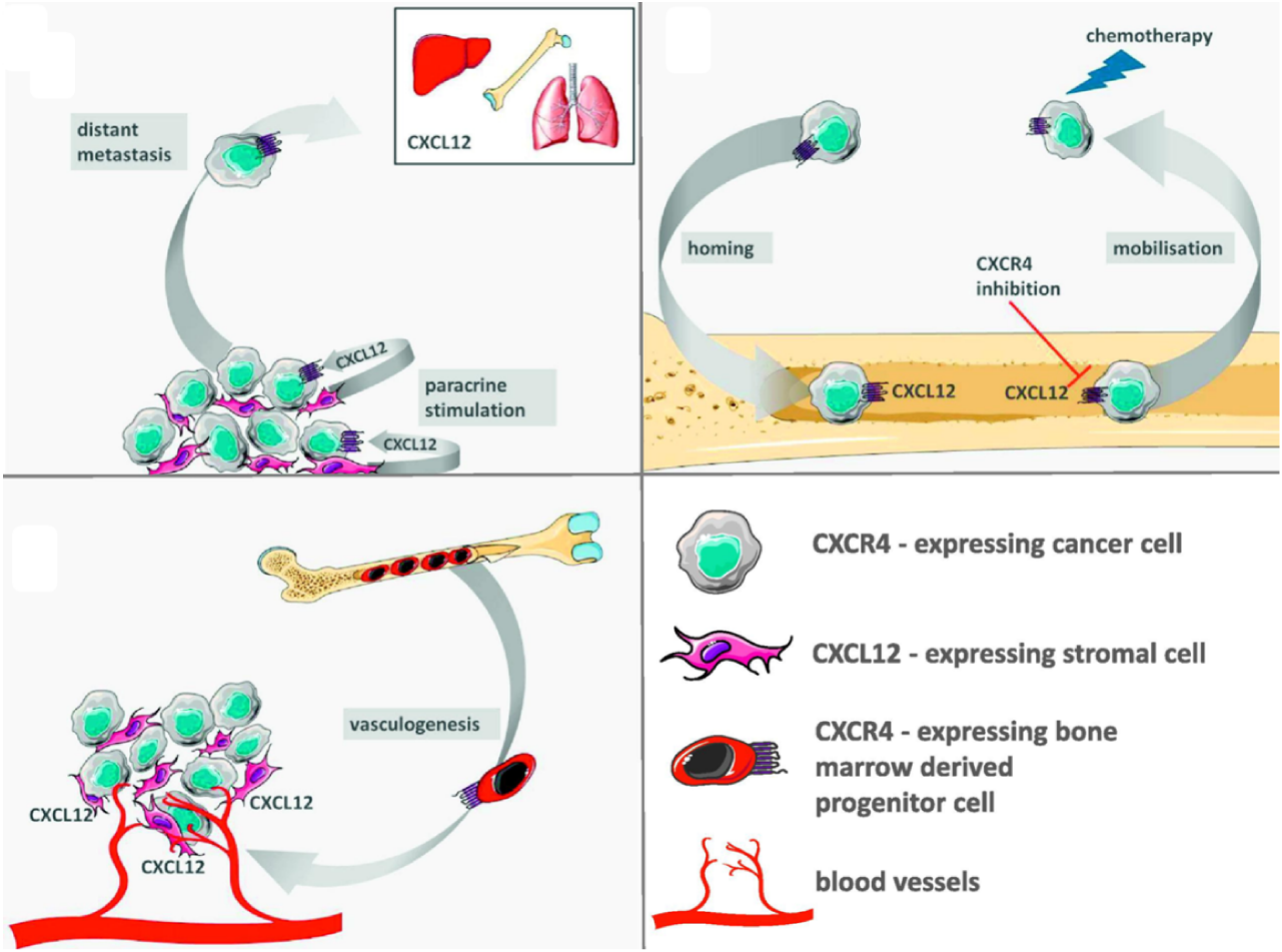
Potential CXCR4-based mechanisms of cancer progression. Reproduced from *European Journal of Cancer* (https://www.sciencedirect.com/science/journal/09598049), Vol. 49, Domanska, U. M.; Kruizinga, R. C.; Nagengast, W. B.; Timmer-Bosscha, H.; Huls, G.; de Vries, E. G. E.; Walenkamp, A. M. E., A Review on CXCR4/CXCL12 Axis in Oncology: No Place to Hide, pp 219–230, Copyright **2013**,^24^ with permission from Elsevier.

The development of CXCR4 antagonists began simultaneously with the discovery of the receptor itself. The identification of AMD3100 (**1)** (Figure 2) as an inhibitor of HIV entry, then by an unknown mechanism, resulted in the finding that this cyclam-containing molecule exhibited antagonism against the CXCR4 receptor.^36^ Ensuing clinical trials in HIV patients resulted in high levels of white blood cells during administration. This led to the successful development of AMD3100 as a hematopoietic stem cell mobilization agent used in the treatment of non-Hodgkin’s lymphoma and multiple myeloma, and the first CXCR4-based FDA-approved drug (Plerixafor).^37^ The next agent of significance to be identified was AMD11070, the first oral small-molecule CXCR4 antagonist (**2)** (Figure 2).^38,39^ Although this compound was initially investigated for HIV infection in the clinic, it has more recently been revived for use in the emerging field of immune oncology.^40,41^ As for the many other preclinical agents identified as small-molecule CXCR4 antagonists, the most notable are GSK812397 (**3**) and the thio-urea IT1t (**4**), for which a cocrystal structure with CXCR4 was solved.^42–45^ Recently, we disclosed a third-generation tetrahydroisoquinoline (THIQ)-based series of CXCR4 antagonists containing a THIQ ring exemplified by the compound TIQ-15 (**5**).^46^ This compound has a set of desirable CXCR4-based properties, including in vitro assay single-digit nanomolar potency, no metabolic effects in human liver microsomes, and even mobilization of white blood cells in mice in a dose-dependent fashion.

**Figure 2:**
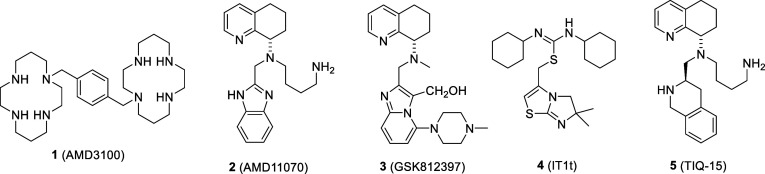
Selected small molecule CXCR4 antagonists (**1**–**5**).

Currently, we have been focused on a lead optimization effort involving **5**, probing SAR to try to improve on the potential liabilities of TIQ-15, which include CYP450 2D6 inhibition, mouse liver microsome instability, and low PAMPA/Caco-2 cell permeability. In this publication, we describe efforts that probed substituents on the phenyl portion of the THIQ ring (Figure 3) and studied the effects on various biological properties, including CXCR4 potency, microsomal stabilities, and CYP450 inhibitions. Previous efforts by our laboratory had produced several alternate series, including naphthyridines and the 2-piperazinyl-based CXCR4 antagonists (**6**).^47^ Our current efforts described herein resulted in the discovery and characterization of a novel series with a 2-N-morpholine substituent as next-generation CXCR4 antagonists (**7**). This came as the result of sequential modifications on TIQ-15 but also a priori from the observation of hypothetically replacing the nitrogen on the piperazine of **6** with an oxygen atom (Figure 3). In previous work, a morpholine replacement in the case of a similar series (**3**, GSK812397) had resulted in a large drop-off in biological activity (>100-fold).^48^ In our case, both approaches gave rise to the identification of the morpholine compounds (**7**) with enhanced potency, which was unexpected. The morpholine ring compounds could provide better physicochemical properties due to the loss of the additional basic site, which includes better permeability and tissue distribution.^49^

**Figure 3:**
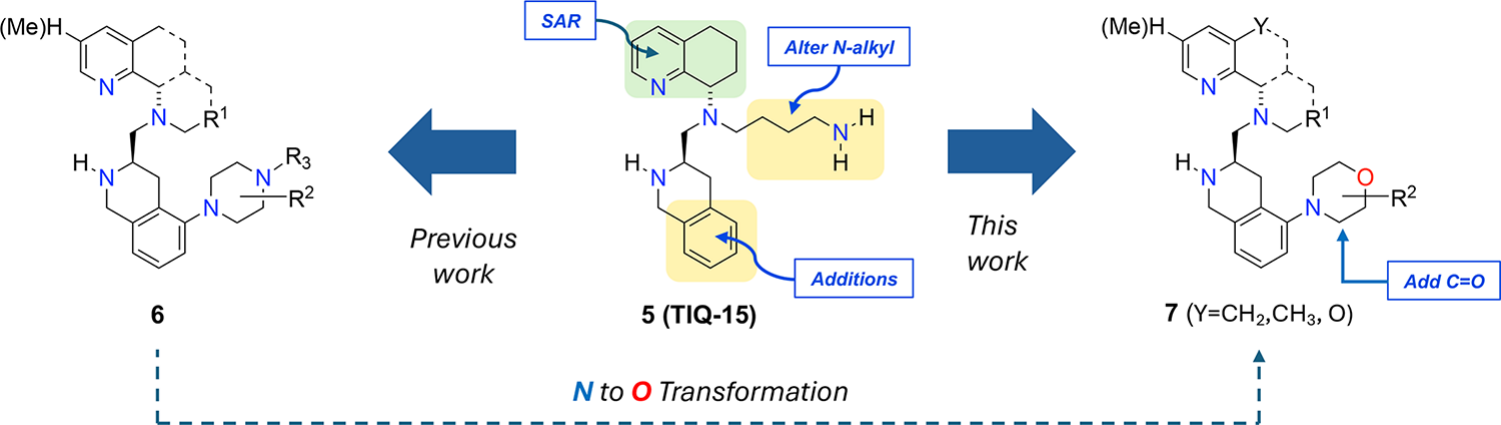
Strategy for modifications of TIQ-15 and previous observations leading to the selection of the morpholine compounds (**7**).

## Results and Discussion

### Stage 1: Identification of 2-Morpholine Tetrahydroisoquinolines

Our efforts began by probing substitutions on the phenyl portion of the THIQ ring with the butyl amine side chain, similar to TIQ-15 (**5**, Scheme 1). We focused primarily on the *R*-stereoisomers as we previously described this stereochemical preference. However, in some cases, the *S*-isomers were also synthesized and submitted for testing. We examined a limited set of groups to probe SAR. First, we performed a chlorine atom walk at the 2, 3, and 4 positions of the THIQ ring (Compounds **15**, **16**, **24**). We included a 1-naphthyl group (**17**), as that has significance in one of the amino acid substitutions on the CXCR4 peptide CVX15 by increasing CXCR4 calcium flux activity 10-fold when a 1-naphthyl alanine is interchanged with a phenylalanine in the sequence. We also synthesized 2-bromo and 2-piperazinyl substitutions, as this position seems to overlap well with the piperazine ring placement on the heterocycle of GSK812397 (**3**) and also correlates to our previous effort (**6**). The synthesis of these derivatives begins with the formation of the THIQ precursors (Scheme 1, **9a**–**c**, **17a**) via a Pictet–Spangler reaction with the corresponding phenylalanine amino acids (**8a**–**e**) in a five-step process. This route involves a somewhat lengthy five-step process, mostly interchanging nitrogen- and carboxy-based protecting groups. In the cases of the 2-chloro, 3-chloro, and 1-naphthyl derivatives, the corresponding *D*-amino acids (**8a**–**c**) were utilized, resulting in *R*-tetrahydroisoquinoline precursors (**9a**–**c**). For the 4-chloro substituent, racemic 4-chlorophenylalanine (**8d**) was utilized. In the case of the 2-bromo and 2-*N*-piperazinyl groups, the commercially available racemic 2-bromo-tetrahydroisoquinoline (**19a**) was utilized. The methyl esters of the THIQ (**20a, 20c**) were then converted to the corresponding aldehydes and combined with the (*S*)-tetrahydroquinoline amine by reductive amination. The corresponding secondary amines were combined with *tert*-butyl­(*tert*-butoxycarbonyl)­(4-oxobutyl)­carbamate (**14**) to form the tertiary amine products, with all amines Boc-protected. The diastereomeric THIQ compounds were separated at this stage into the *S*,*R* and *S*,*S* compounds. Deprotection was then performed with TFA to afford the final compounds.

**Scheme 1:**
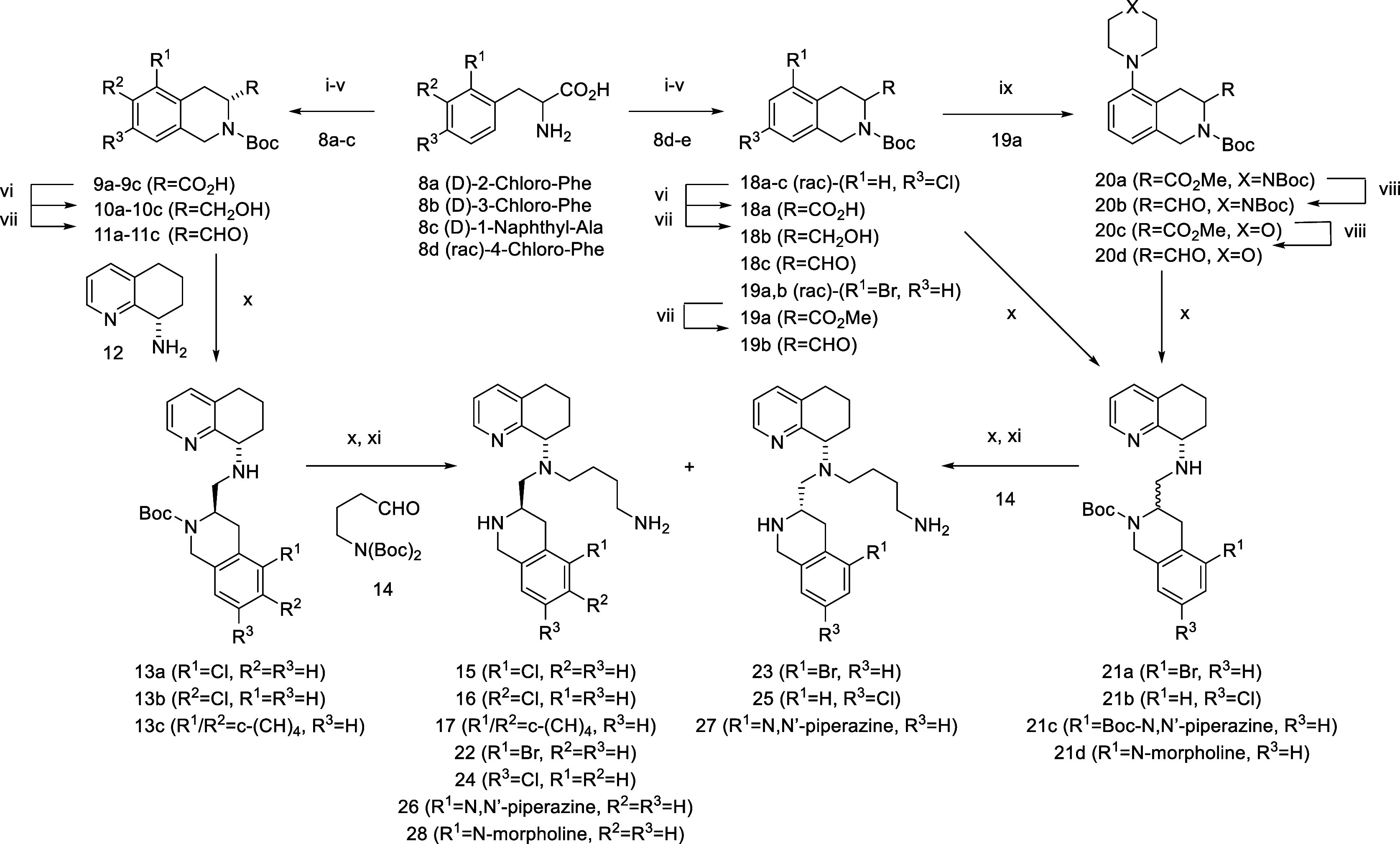
^a^

a Reagents: i. SOCl_2_, MeOH, 0–55 °C; ii. EtCO_2_Cl, Pyridine; iii. HCHO, AcOH:H_2_SO_4_ (3:1); iv. LiOH.H_2_O, 180 °C or 6 M HCl, 130 °C; v. Boc_2_O, NaOH, MeOH; vi. BH_3_.Me_2_S, 0 °C, THF; vii. Pyr-SO_3_, Et_3_N, DMSO–DCM or Dess–Martin Periodinane, DCM; viii. DIBAL-H, −78 °C, Toluene; ix. Pd_2_(dba)_3_, BINAP, Cs_2_CO_3_, N-Boc-piperazine or morpholine, 120 °C, Toluene; x. **12** NaBH­(OAc)_3_, DCM; xi. TFA, DCM.

The butylamine derivatives were screened in a battery of pharmacological assays to assess the potential of these modifications for on-target potency and preliminary absorption, distribution, metabolism, and elimination (ADME) properties. Specifically, on-target potencies were determined in both CXCR4 calcium flux and Forskolin-stimulated cAMP assays using CXCL12. The CYP450 inhibition against the two most significant isozymes, 3A4 and 2D6, was determined with fluorogenic substrates, which would predict drug–drug interaction potential. Metabolic stability in liver microsomes was determined for two species (human and mouse) by the percentage of compound remaining at 10 min to estimate first-pass metabolism effects. This set of assays was used to determine structure–activity relationships (SARs) and to define areas to both improve and optimize compound properties (Table 1).

**Table 1:**
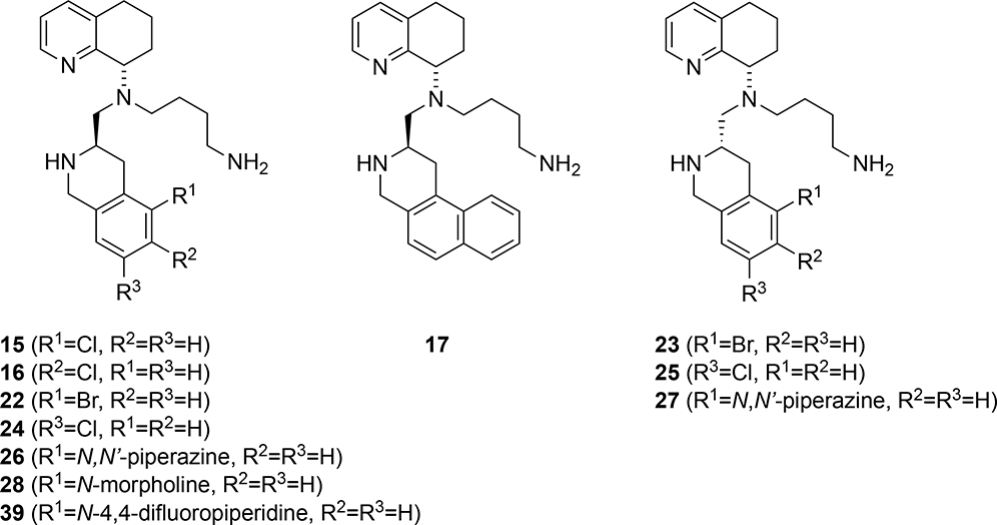
Biological Effects of THIQ Phenyl Ring Substitutions on TIQ-15

			CYP450 IC_50_ (μM)		Liver Microsomes % rem. @ 10 min.	
Compd. No.	CXCR4 Ca^2+^ Flux IC_50_ (nM)	CXCR4 cAMP IC_50_ (nM)	3A4	2D6	H	M
**TIQ15**	7.00^a^	41.0^a^	>20	0.33	77	17
**15**	8.60 ± 0.00^b^	>10000^a^	13.7	0.49	75.2	65.4
**16**	9.33 ± 0.00^b^	51.2 ± 6.76^b^	>20	0.54	95.2	27.5
**17**	20.2 ± 0.00^b^	812 ± 416^b^	4.14	0.35	87.7	37.8
**22**	24.5^a^ ^a^	49.6 ± 6.59^b^	13.5	0.33	77.3	84.4
**23**	178 ± 27.0^b^	298 ± 87.0^b^	9.76	0.57	96.1	73.0
**24**	21.4^a^	125 ± 69^b^	>20	0.42	84.7	2.75
**25**	90.9^a^	>10000^a^	>20	0.04	100	100
**26**	94.4^a^	52.3 ± 6.18^b^	>20	>20	100	N.D.
**27**	801^a^	159 ± 41.8^b^	>20	14.5	100	73.0
**28**	30.1 ± 16.0^b^	45.5 ± 14.1^b^	>20	18.9	92.3	76.9
**39**	30^a^	163 ± 33.1^b^	13.0	3.5	100	90.7

a Single run in duplicate.

b Double run in duplicate.

First, the chlorine atom walked from the 2, 3, and 4 positions (**15**, **16**, **24**) on the THIQ ring. The chlorine atom maintained on-target potency without compromising tolerability. The CXCR4 calcium flux IC_50_ values for the 2 (**15**) and 3 (**16**) substitutions were identical to TIQ-15 (9 versus 7 nM), and even the 4-chloro compound (**24**) was only about 3-fold higher (21 nM). However, it was the other assay results that proved to differentiate the chlorine compounds from TIQ-15. For example, for the 2-chloro compound (**15**), a slight increase in CYP450 3A4 inhibition was observed, while 2D6 remained below 1 μM for all three compounds. All three compounds had equally good stability compared to TIQ-15 in human liver microsomes (>70% remaining) but differed in mouse liver microsomal stabilities. Ironically, the 3-chloro derivative (**16**) seemed nearly identical to TIQ-15 in all properties. Therefore, the chlorine atom walk failed to provide any meaningful improvements over TIQ-15 regarding CYP inhibition.

Next, we turned to substitutions that would increase the steric bulk on the THIQ phenyl ring and also increase hydrophobicity. First, the 2-bromo substituent (**22**) showed a slight increase in CXCR4 activity (3-fold) as well as a significant increase in CYP450 3A4 isozyme inhibition. The 1-naphthyl substituent (**17**), which spans the 2 and 3 positions, was 3-fold less active than TIQ-15, and it also had a significant increase in CYP450 3A4 inhibition. Neither compound improved the CYP450 2D6 activity, while the 2-bromo compound did improve the mouse liver microsomal stability. Again, we were faced with an unsuccessful effort, as no improvements were seen with these two compounds versus TIQ-15.

The last effort in the butyl amine side chain series involved placing nitrogen rings at the 2-position. We knew from the literature that 2-amino groups might be tolerated in this portion of the molecule based on the efforts involving the identification of GSK-812397 (**3**) as well as the previous efforts from our laboratory.^37,42^ First, the compounds with the piperazine ring (**26, 27**; Table 1), synthesized via a Buchwald coupling reaction sequence from the racemic 2-bromo THIQ compound (**19a**, Scheme 1), were submitted to the assay sequence. The piperazine compound (**26**) showed significantly less activity (IC_50_ = 121 nM, 10- to 15-fold) in the CXCR4 calcium flux assay. However, improvements in the CYP450 2D6 inhibition and liver microsomal stabilities were observed versus TIQ-15. It is also worth noting that the opposite THIQ ring isomers with the 2-piperazinyl (**27**), the 2-bromo (**23**), and 4-chloro (**25**) substituents all showed lower CXCR4 potency (>10-fold) versus their R-ring counterparts (**22, 24, 27**). Although, in the case of the 2-bromo compound (**23**), we observed a closer margin in CXCR4 potency (8-fold) with better mouse liver microsomal stability, we concluded that our synthetic efforts in the future should be directed solely toward the R-isomers.

Our final effort here involved placing a morpholine at the 2-position on the THIQ ring of TIQ-15. The morpholine ring is a drug-like fragment found in many preclinical, clinical, and human therapeutics.^49^ The 2-morpholine compound (**28**) was synthesized via an enantioselective route from compound **28** (Scheme 2)^50^ via a Buchwald and reductive amination sequence. The 2-morpholino analog of TIQ-15 (**28**) was subjected to the same bioassays (Table 1), and some surprising improvements were observed. Overall, the 2-morpholine compound **28** had a better profile with some small exceptions. This compound had much lower CYP450 2D6 activity (>50-fold, IC_50_ = 18.9 μM) as well as marked improvement in mouse liver microsomal stability (77% versus 17%) versus TIQ-15, with only a 4- to 5-fold drop in CXCR4 potency in the calcium flux assay but not in the cAMP assay. Overall, the morpholine compound (**28**) had improvements in metabolic stability and CYP450 inhibition versus TIQ-15, providing the best ring substitution investigated in this study. Having good improvements with the morpholine substitution, we also synthesized the 4,4-difluoropiperidine derivative (**39**, Table 1) as a morpholine isostere. However, this substitution resulted in a loss of some of the CXCR4 activity and an increase in CYP450 inhibition. Therefore, based on these findings, further exploration of the effects of adding morpholine with other structural variants was warranted by expanding the SARs around compound **28**.

**Scheme 2:**
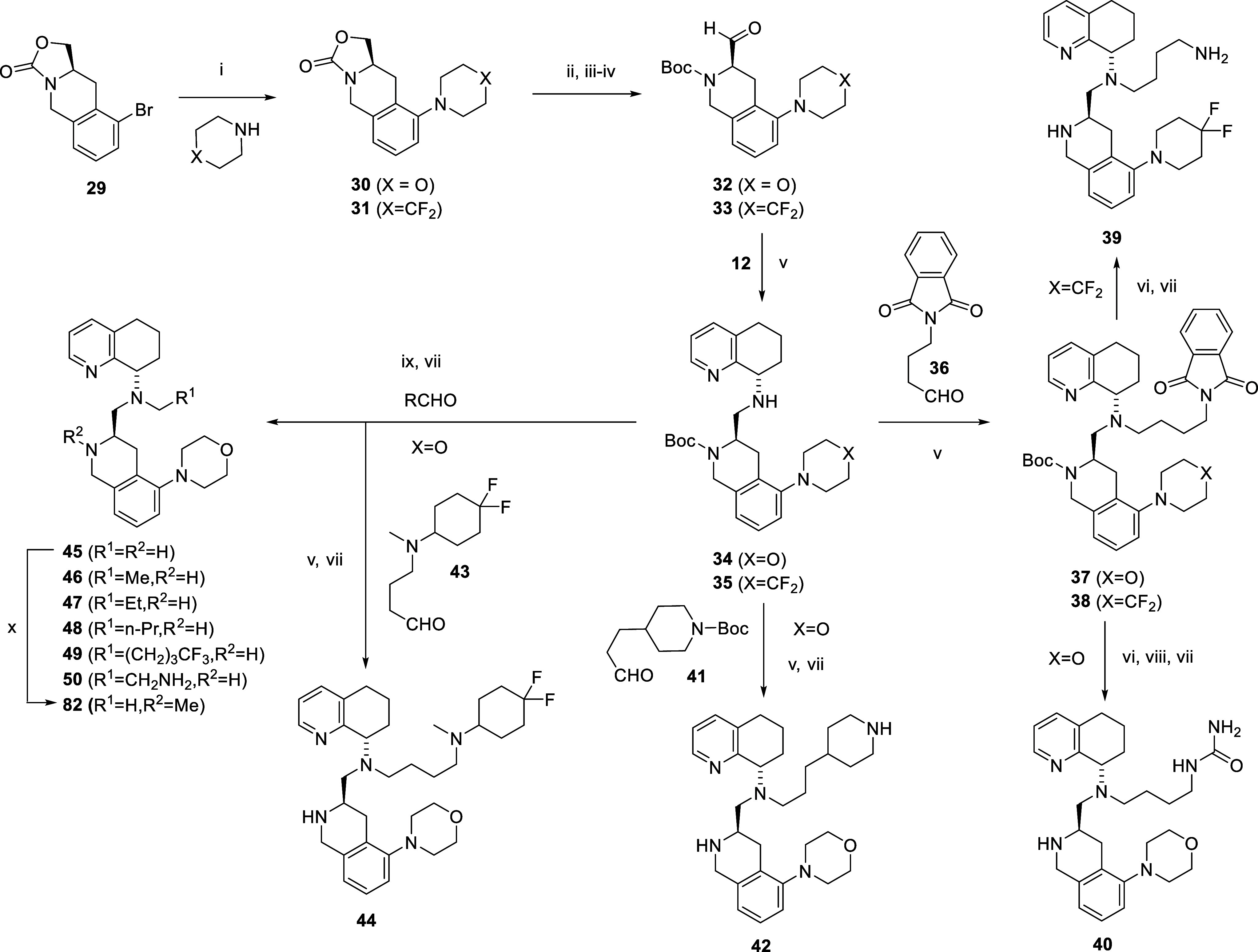
^a^

a Reagents: i. Morpholine or 4,4-difluoropiperidine, Pd_2_(dba)_3_, BINAP, Cs_2_CO_3_, toluene, 120 °C; ii NaOH, MeOH; iii. (Boc)_2_O, NaOH, THF; iv. Py-SO_3_, NEt_3_, DCM-DMSO; v. **36**, **41,** or **43**, NaBH­(OAc)_3_, 1,2-DCE, r.t.; vi. NH_2_NH_2_–H_2_O, MeOH, r.t.; vii. TFA, DCM, r.t.; viii. TMSNCO, DIPEA, THF, r.t.; ix. RCHO, NaBH­(OAc)_3_, 1,2-DCE, r.t.; x. CH_2_O, NaBH­(OAc)_3_, DCM, r.t.

Next, we decided to expand around the SAR of the 2-morpholine compound **28,** given its superior in vitro properties. First, we decided to probe the role of the *n*-butyl amine side chain, as we had done in the cases of both the TIQ-15 and 2-piperazine series.^46,50^ Initially, we targeted the previously identified advantageous substitutions, such as the propyl piperazine^51^ and methyl groups. These compounds were synthesized by slightly different routes (Schemes 2 and 3). We greatly expanded these efforts to include compounds **40**, **42**, **44**, **46–50**, **53**, **58,** and **61**. Most compounds were synthesized with reductive amination reactions using the corresponding aldehyde and STAB, followed by Boc deprotection with TFA. Other compounds (**40**, **53**, **58,** and **61**) were synthesized via a different route (Schemes 2 and 3). The 2-morpholine intermediate **34** was synthesized via an enantioselective route from compound **32** (Scheme 2, synthesized according to literature procedures)^50^ via a Buchwald and reductive amination sequence. Intermediate **34** was used to synthesize **42,** and **44–50** via reductive amination with the appropriate aldehyde (RCHO; **36**,**41**,**43**) and STAB in 1,2-dichloroethane, followed by Boc deprotection with TFA. Compound **40** was synthesized from **34** with phthalimide-protected butyraldehyde **36** via reductive amination, deprotection of the phthalimide group with hydrazine hydrate, urea formation by reaction of the primary amine with trimethyl isocyanate, and deprotection of the Boc group with TFA. Compound **53** was synthesized stepwise, starting from compound **12** and reacting with aldehyde **51** to give intermediate **52,** followed by reaction with aldehyde **32** and deprotection of the t-butyldimethylsilyl group (Scheme 3). The propyl piperazine compounds **58** (N–H) and **61** (N-ethyl) were synthesized via different pathways involving N-protected intermediates (Scheme 3). Compound **12** was reacted with Boc-protected propyl-piperazine bromide via literature procedures^51^ and followed by reductive amination with **32** and deprotection of the Boc group with TFA to give compound **58**. Next, compound **61** was synthesized by the following sequence: compound **12** was combined with Cbz-protected propyl-piperazine bromide **55**, then Boc protection; Cbz deprotection; acetaldehyde reductive amination; and finally, deprotection of the Boc group to give intermediate **60**. Finally, reductive amination with **32**, followed by TFA deprotection, gave compound **61**. Initial screening comparisons of these two compounds showed that the propyl piperazine compounds **58** and **61** offered similar properties to the butyl amine compound **28**, with higher inhibition against CYP 2D6 but lower potency against CXCR4 in the cAMP assay. The *N*-methyl compound **45** was synthesized via a previously described late-stage Buchwald reaction sequence via the bromide **37** and morpholine, and then Boc deprotection with TFA (Scheme 2).

**Scheme 3:**
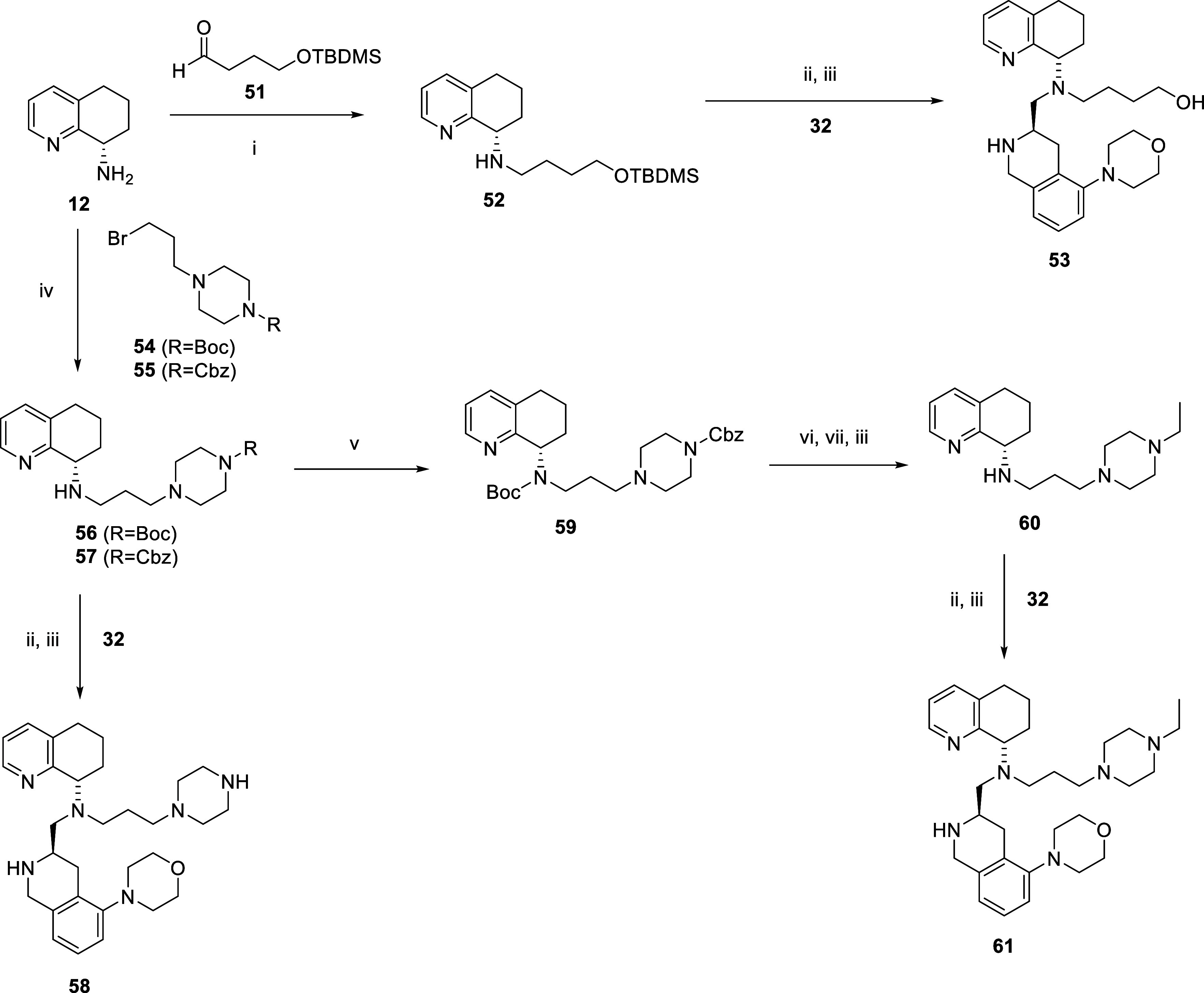
^a^

a Reagents: i. **51**, Ti­(OiPr)_4_, NaBH­(OAc)_3_, THF, r.t.; ii. **32**, Ti­(OiPr)_4_, NaBH­(OAc)_3_, 1,2-DCE; iii. TFA, DCM; iv. **54** or **55**, MeCN; v. (*t*-BuCO_2_)_2_O, 1N NaOH, 1,4-Dioxane; vi. PdOH (C), NH_4_CHO_2_, EtOH; vii. CH_3_CHO, NaBH­(OAc)_3_, 1,2-DCE.

These new side chain analogs were screened in the previously described assays (Table 2) with mixed results. All the compounds had higher potency in the cAMP assay, which varied from 5- to 200-fold (IC_50_ values from 10 nM to 1,530 nM). All the side chains with heteroatoms showed improvement in CYP 2D6 inhibition. These include the hydroxyl, urea, amino, trifluoromethyl, difluorocyclohexyl amino, and piperidinyl groups. The exceptions were the simple alkyl side chains (Et, nPr, nBu), which showed similar single-digit μM potency in CYP 2D6 inhibition. However, many of the compounds unexpectedly displayed CYP 3A4 inhibition, indicating a limit to our modifications. Furthermore, many had metabolic stability that was only slightly improved. Then, there were compounds that had good metabolic stability but stronger CYP 3A4 inhibition (**50** and **61**) or no CYP 2D6 inhibition but lower metabolic stability (**44**). Overall, the best compounds in this second modification phase were the propyl piperazine **58** and piperidine **42** that exhibited improved CYP 450 inhibition and metabolic stability compared to TIQ-15, albeit at the cost of 10-fold lower on-target CXCR4 efficacy. Overall, the on-target activity of the *N*-methyl derivative **45** in the calcium flux and cAMP assays showed excellent potency (Table 2), providing the best overall compound. In the calcium flux assay, both **45** and **28** turned out to have similar potency (Table 2) but 5- to 6-fold more activity on cAMP assay, favoring the methyl derivative. We fully expected the on-target potency to be much higher due to the loss of the potential for forming a salt bridge involving the butyl amine side chain and an aspartic acid residue with the receptor, based on our previous modeling studies.^46^ However, it seems that the morpholine oxygen may be providing this interaction with the ring oxygen atom via an oxygen–hydrogen bond as a replacement for the nitrogen atom in the piperazine ring from our earlier reported series (**6**). Also worth noting is that the compound was four times more potent than the n-propyl piperazine side-chain compound **58,** indicating that both the type and positioning on the hydrogen bond acceptor are important factors. These results indicate that the primary role of these substituents is as a hydrogen bond acceptor.

**Table 2:**
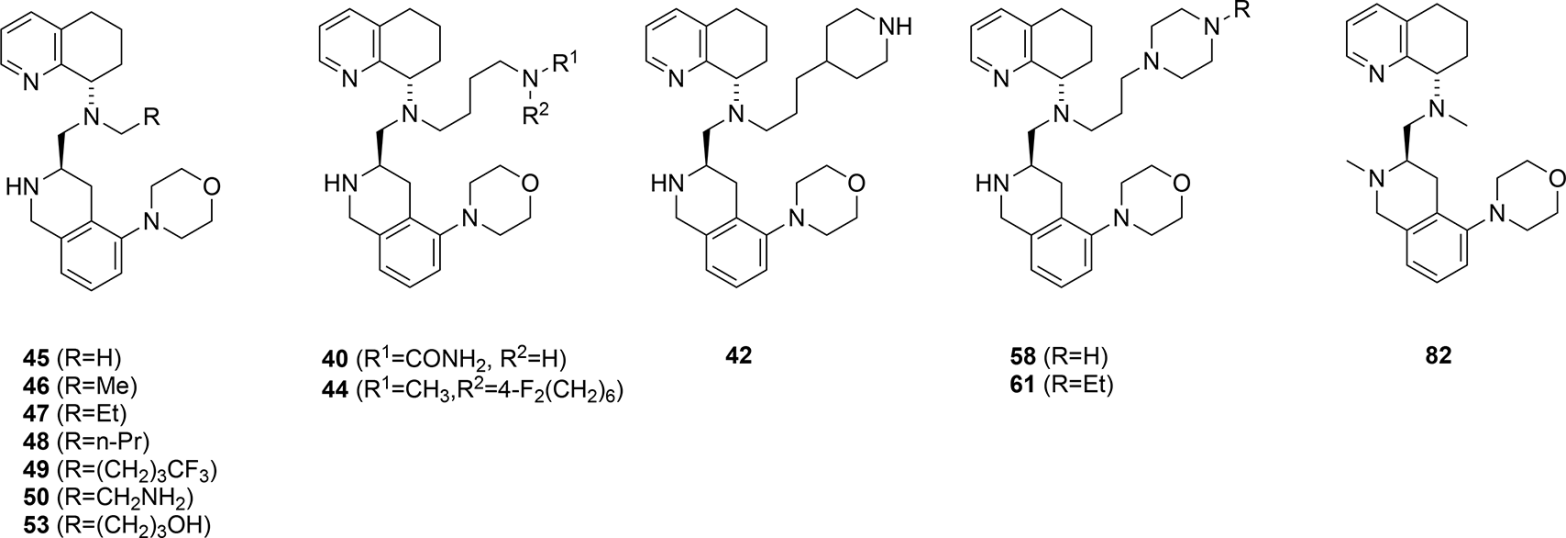
Butyl Amine Side Chain Variation

			CYP450 IC_50_ (μM)		Liver Microsomes % rem. @ 10 min.	
Compd. No.	CXCR4 Ca^2+^ Flux IC_50_ (nM)	CXCR4 cAMP IC_50_ (nM)	3A4	2D6	H	M
**28**	30.1 ± 16^a^	45.5 ± 14.1^a^	>20	18.9	92.3	76.9
**40**	41^b^	187 ± 26.0^a^	9.57	>20	33.5	68.5
**42**	18^b^	136 ± 36^a^	>20	>20	72.8	87.3
**44**	61^b^	377 ± 270^a^	>20	>20	30.2	78.5
**45**	23.8 ± 11.5^a^	8.25 ± 4.00^a^	>20	4.28	46.0	0.71
**46**	9.6^b^	76.6 ± 21.2^a^	>20	3.10	17.9	17.3
**47**	88^b^	453 ± 110^a^	17.1	18.9	12.3	65.9
**48**	120^b^	281 ± 162^a^	8.10	2.58	19.3	61.1
**49**	130^b^	444 ± 166^a^	9.60	>20	19.4	36.4
**50**	250^b^	177 ± 67.4^a^	12.4	>20	97.2	100
**53**	26^b^	1053 ± 820^a^	17.3	>20	31.8	45.6
**58**	145 ± 0.0	104 ± 36.4^a^	>20	>20	80.7	99.7
**61**	19^b^	387 ± 176^a^	17.3	>20	56.6	84
**82**	571^b^	47.1 ± 16.0^a^	>20	11.5	12	5

a Double run in duplicate.

b Single run in duplicate.

### Stage 2: Optimization of 2-Morpholine *N*-Methyl Tetrahydroisoquinoline Compounds

Although **45** provided encouragingly low CXCR4 assay potency, it also possessed weak CYP 2D6 inhibition and low metabolic stability in mouse liver microsomes. Therefore, we turned our attention to modifications on the core of **45** (Scheme 4). Literature shows that compounds with morpholine rings undergo metabolism, mostly arising from oxidation occurring on the ring carbon atoms. Therefore, the addition of steric and electronic groups would be expected to increase metabolic stability. The substituted morpholine ring analogs—mono methyl (**71**, **72**), dimethyl morpholine (**73**), bicyclic morpholine (**74**), and hydroxymethyl morpholine (**75**, **76**)—were synthesized via a late-stage Buchwald route, followed by deprotection of the Boc group with TFA. Also, we made the morpholinone, which would be expected to curb both CYP 450 inhibition and metabolic degradation (**81**) using an Ulman-type coupling (N, N’-dimethylethane-1,2-diamine, potassium carbonate, copper iodide, toluene, 110 °C). Compound **69** was synthesized following the literature procedure.^50^ Compounds **71–78** were synthesized via a Buchwald reaction from **69,** followed by deprotection with TFA in moderate yields. Compound **81** was synthesized from **69** using morpholine-3-one, potassium carbonate, copper iodide, and N,N’-dimethylethane-1,2-diamine conditions and followed by deprotection with TFA. Compounds **79** and **80** were synthesized via an early stage Buchwald reaction from **27** to get intermediates **63** and **64**. The aldehydes were synthesized from **63** and **64** via ring opening with NaOH hydrolysis, followed by Boc protection and subsequent oxidation under Parikh–Doering conditions to get **65** and **66**. Compounds **79** and **80** were synthesized from **65** and **66** using amine **67** via reductive amination, followed by TFA Boc deprotection. Compound **82** was synthesized starting with compound **45** via reductive amination using formaldehyde.

**Scheme 4:**
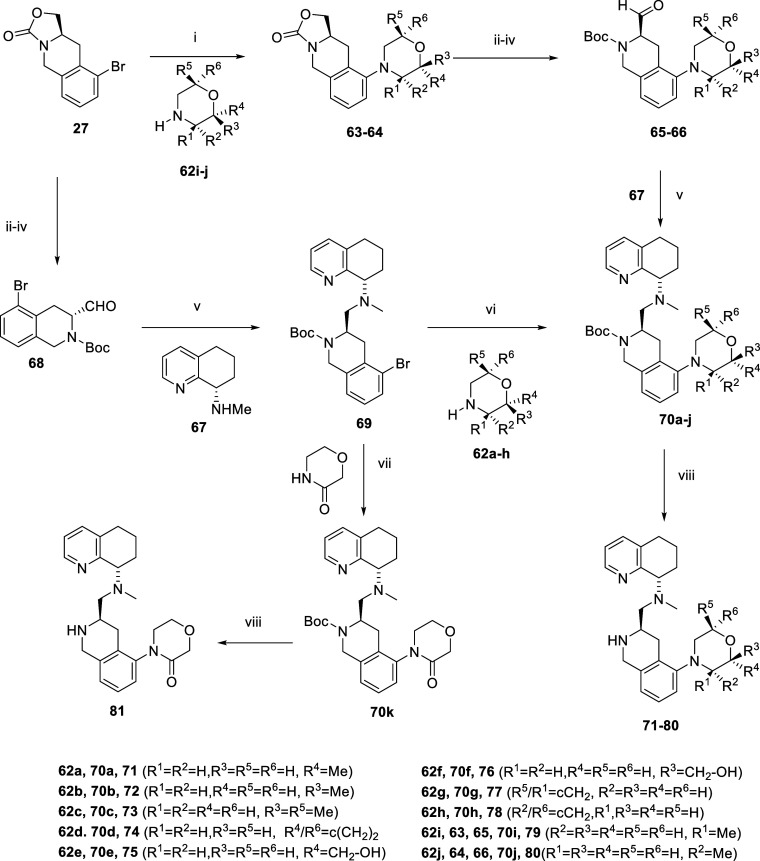
^a^

a Reagents: i. **62i** or **62j**, Pd_2_(dba)_3_, BINAP, Cs_2_CO_3_, Toluene, 120 °C; ii NaOH, MeOH, 100 °C; iii. (Boc)_2_O, NaOH, THF, r.t.; iv. Py-SO_3_, NEt_3_, DCM-DMSO, 0 °C; v. **67**, NaBH­(OAc)_3_, 1,2-DCE, r.t.; vi. **62a**–**h**, Pd_2_(dba)_3_, BINAP, Cs_2_CO_3_, toluene, 120 °C; vii. **62i**, K_2_CO_3_, CuI, MeNH­(CH_2_)_2_NHMe, Toluene, 110 °C; viii. TFA, DCM, r.t.

Next, we tested the morpholine-modified compounds in the assay panel used in the previous evaluations (Table 3). Our findings showed that overall modifications resulted in comparable or slightly higher potency in the on-target CXCR4 assays, with IC_50_ ranges of 32–270 nM in the calcium flux and 23–173 nM in the cAMP assays. The notable exception was the 2,6-dimethyl morpholine compound (**73)** (IC_50_ values of 4,705 and 1,206 nM). Also notable was the nearly 10-fold difference observed for the stereochemical partners of the 2-methyl and 2-oxa-5-azabicyclo[2.2.1]­heptanes (**79**–**81**) which could provide some insight into compound binding modes. Most compounds had less potent CYP 2D6 values, but this came at the cost of either less potent CXCR4 activity or increased CYP 3A4 potency. However, there were four compounds that had both CXCR4 IC_50_ values below 100 nM and CYP 450 IC_50_ values greater than 10 μM (**75**,**76**,**79**,**81**). Furthermore, there were five compounds with better metabolic stability than **45** (**75**–**78**, **81**). The best compounds were the hydroxymethyl derivatives **75** and **76** and the morpholinone **81**. All three had better CYP 450 and metabolic stability profiles than **45,** with only slightly higher on-target CXCR4 potencies.

**Table 3:**
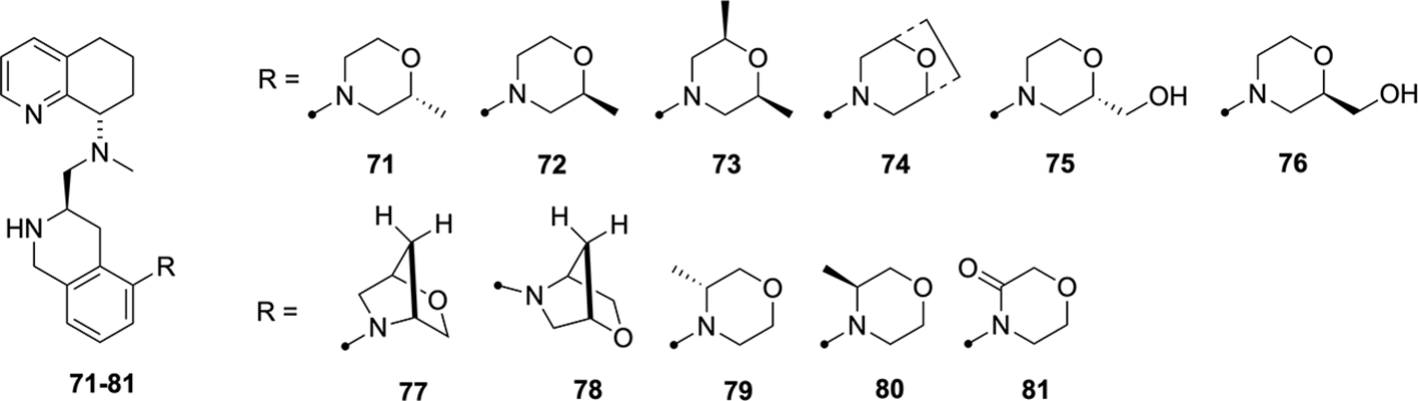
Structural Changes to the Morpholine Ring of **45**

			CYP450 IC_50_ (μM)		Liver Microsomes %rem. @ 10 min.	
Compd. No.	CXCR4 Ca^2+^ Flux IC_50_ (nM)	CXCR4 cAMP IC_50_ (nM)	3A4	2D6	H	M
**45**	24 ± 12^a^	8.25 ± 4.00^a^	>20^a^	4.28^b^	46.0	0.71
**71**	40^b^	23.2 ± 10.7^a^	>20^a^	6.2 ± 0.34^a^	37.6	29.0
**72**	270^b^	173 ± 79.3^a^	6.0 ± 2.8^a^	11.1 ± 0.6^a^	26.4	10.0
**73**	4705^b^	1210 ± 594^a^	15.8^b^	4.90^b^	13.8	6.3
**74**	80^b^	42.0 ± 6.28^a^	16.8 ± 2.4^a^	9.8 ± 0.47^a^	36.8	38.7
**75**	78^b^	37.8 ± 12.7^a^	14.9 ± 6.4^a^	>20^a^	94.4	38.0
**76**	50^b^	82.4 ± 29.2^a^	16.0 ± 1.5^a^	>20^a^	80.6	42.6
**77**	120^b^	164.7 ± 41.8^a^	6.0 ± 0.9^a^	>20^a^	63.6	29.7
**78**	31^b^	55.0 ± 20.0	3.5 ± 0.7^a^	>20^a^	48.5	36.9
**79**	32^b^	81.7 ± 50.8	17.3 ± 7.1^a^	>20^a^	5.3	29.3
**80**	57^b^	51.7 ± 30.7	9.3 ± 8.9^a^	>20^a^	9.7	49.7
**81**	68.9^b^	48.3 ± 6.62	32.6 ± 5.0^a^	>50^a^	79.1	83.2

a Double run in duplicate.

b Single run in duplicate.

Our last chemistry exploration involved changes to the top tetrahydroquinoline (THQ) ring to try to increase potency and metabolic stability. Here, we picked only two morpholine substitutions to be investigated with various THQ replacements: morpholine and 2-morpholinone (Schemes 5, 6). We selected different configurations, including methyl- and oxygen-substituted THQ versions, 3,5-dimethyl-pyridine, octahydronaphthyridine, and 2-amino-quinoline. The compounds were synthesized via mostly a late-stage Buchwald strategy, where bromo-aldehyde **68** was reacted with THQ pieces (**83**,**87**,**88**,**95**, Scheme 5) or amino-quinoline (**99**) followed by methylation and/or Boc deprotection (Scheme 6). Compound **83** was synthesized according to literature procedures.^52^ Compound **84** was synthesized from **83** with **68** via reductive amination and then subjected to a Buchwald reaction with morpholine, followed by deprotection to give **85**. Compound **84** was reacted with morpholine-3-one, with potassium carbonate, copper iodide, and N, N’-dimethylethane-1,2-diamine in toluene at 110 °C, followed by deprotection with TFA to give **86**. Analogously, compounds **91** and **92** were made from **88** and **90**, **93** and **94** from **87** and **89**, and **97** and **98** from **95** and **96** (Scheme 5). Compound **101** was synthesized from amino-quinoline **99** by reaction with **32** to give **100**, followed by methylation and Boc deprotection (Scheme 6).

**Scheme 5:**
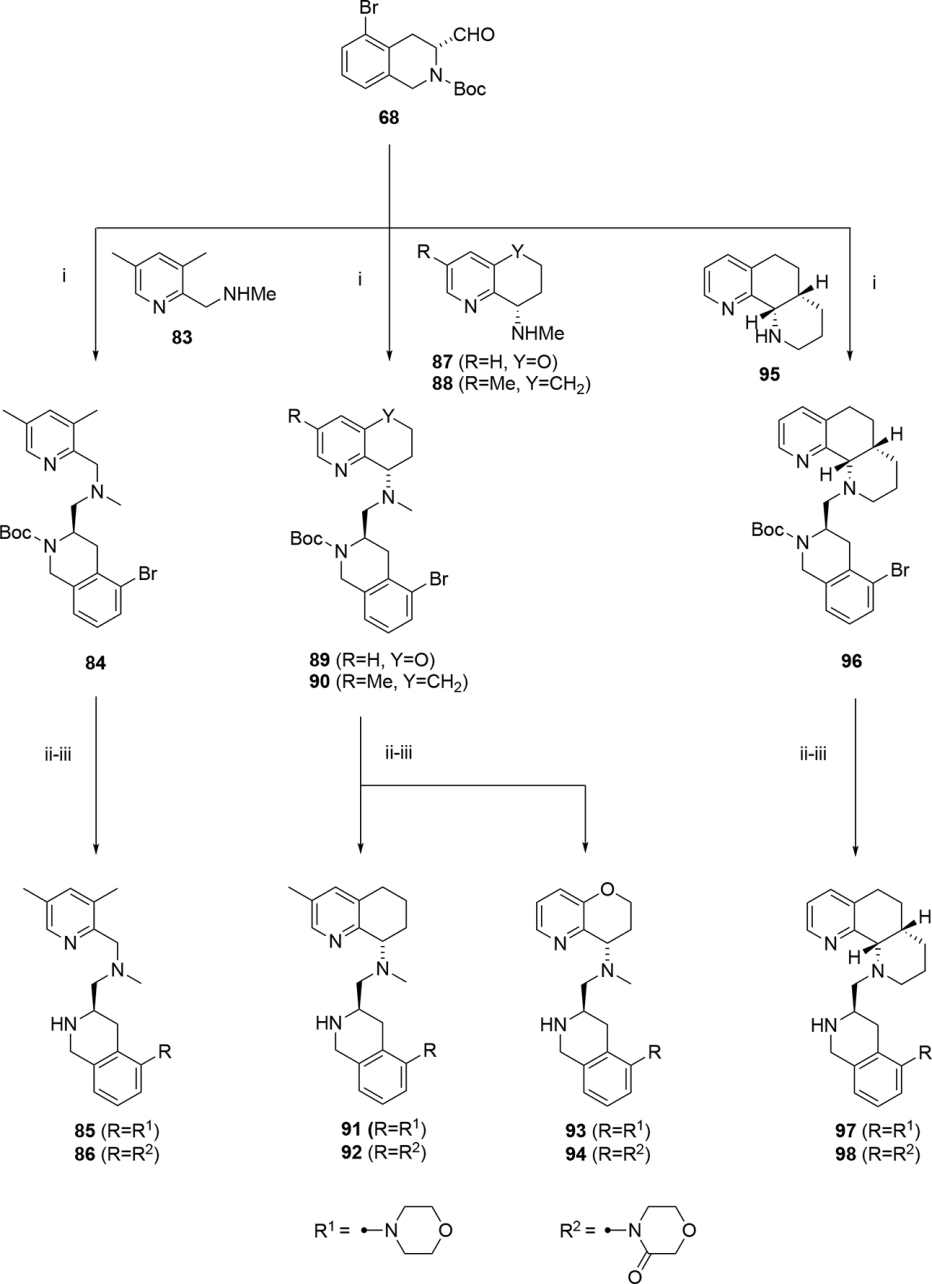
^a^

a Reagents: i. **83**, **87**, **88**, or **95**, NaBH­(OAc)_3_, AcOH, DCM, r.t.; ii. morpholine, Pd_2_(dba)_3_, BINAP, Cs_2_CO_3_, Toluene, 120 °C or morpholine-3-one, K_2_CO_3_, CuI, MeNH­(CH_2_)_2_NHMe, Toluene, 110 °C, iii. TFA, DCM, r.t.

**Scheme 6:**
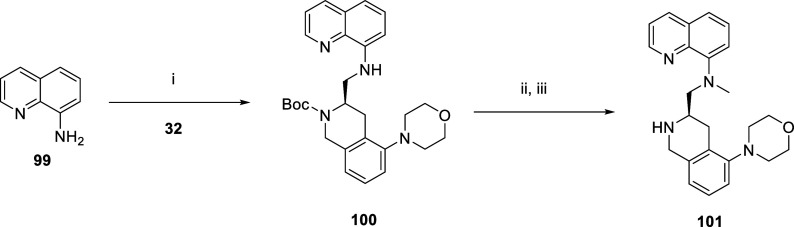
^a^

a Reagents: i. NaBH­(OAc)_3_, 1,2-DCE, r.t.; ii. 37% HCHO (H_2_O), NaBH­(OAc)_3_, 1,2-DCE, iii. TFA, DCM, r.t.

The compounds were profiled through our assay panel, and the results were obtained (Table 4). Overall, the results were different than those previously obtained in the piperazine series (**6**). Here, both the dimethylpyridine (**85**,**86**) and octahydronaphthyridine (**97**,**98**) compounds provided higher on-target CXCR4 potencies than our previous series involving either the 2-piperazinyl or *n*-butylamine side chains, which was unexpected. Although all compounds had better mouse liver microsomal stability, most had higher CYP 2D6 inhibition than **45**, and none had better overall properties than **45** or **81**. The oxygen-inserted compounds had either lower CYP 3A4 inhibition (**93**) or higher CXCR4 on-target efficacy (**94**). The best alternate THQ substitution in this effort was the 5-methyl-THQ derivative **92**, which had improved metabolic stability and CYP 2D6 inhibition compared to **45** but not **81**. The amino-quinoline compound (**101**) did not provide improvements in any areas. Additionally, there were improvements in CYP 450 and metabolic stability for some compounds, but at the cost of on-target potency.

**Table 4:**
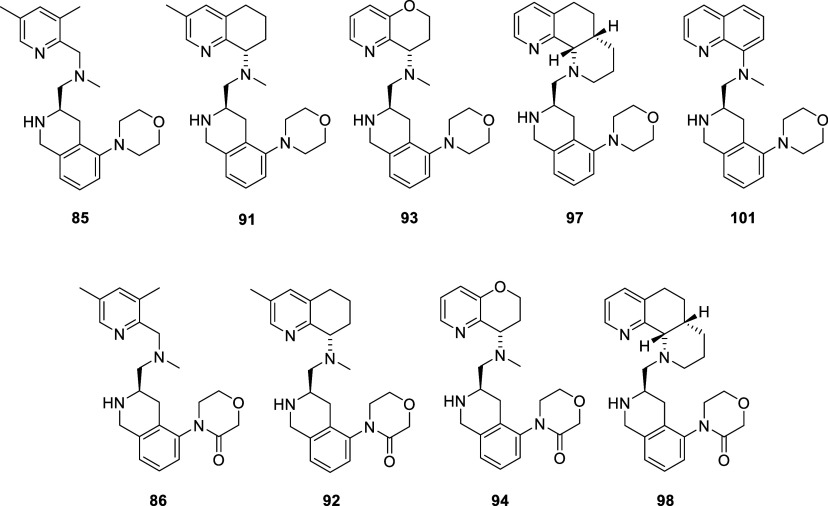
Top Piece Variations

			CYP450 IC_50_ (μM)		Liver Microsomes %rem. @ 10 min.	
Compd. No.	CXCR4 Ca^2+^ Flux IC_50_ (nM)	CXCR4 cAMP IC_50_ (nM)	3A4	2D6	H	M
**85**	28^a^	412 ± 284^b^	12.2 ± 0.4^b^	13.0 ± 4.8	43.2	54.5
**86**	1000^a^	7110 ± 2000^b^	17.4 ± 1.6^b^	>20^b^	81.4	73.5
**91**	98^a^	27.8 ± 25.2^b^	13.1 ± 1.0^b^	7.2 ± 1.2^b^	41	35
**92**	23^a^	125 ± 96^b^	14.0 + 9.0^b^	28.0 + 2.0^b^	86	80
**93**	26^a^	83.8 ± 42.4^b^	4.6 ± 0.1^b^	10.4 ± 4.2^b^	73.5	80.9
**94**	650^a^	1600 ± 970^b^	>20^b^	>20^b^	84.7	87.2
**97**	50^a^	313 ± 135^b^	8.7 ± 0.4^b^	11.0 ± 3.5^b^	35.3	51.4
**98**	2700^a^	327 ± 223^b^	>20^b^	>20^b^	61.5	69.3
**101**	180^a^	5590 ± 2850^b^	3.4 ± 1.4^b^	4.3 ± 0.25^b^	61.4	71.3

a Single run in duplicate.

b Double run in duplicate.

### Modeling Studies to Explore Mechanisms of Binding

Molecular modeling of TIQ-15 and the CXCR4 receptor utilizing the two crystal structures with small molecule IT1t and peptide CVX15 (3ODU and 3OE0; Schroedinger Maestro) has been described by us previously.^53^ In the current series, modeling of the key morpholine compounds **28**, **45,** and **81** was performed. The compounds were docked into both the IT1t and CVX15 crystal structures following the methodology described in our previous study, where key residues were identified as important to the binding modes of the tetrahydroquinoline and other portions of this series. In these grids, the glutamic acid residue E288 was used as a constraint based on the earlier mutational data performed on TIQ-15, since this was the most critical residue in the binding studies. The mutational data showed that when the E288 residue was switched with alanine (A288_MUT_), the functional response to TIQ-15 was completely reversed, and the IC_50_ went from 83 nM to >10,000 nM. In the case of the IT1t grid, two secondary residue constraints were introduced based on our earlier generated poses of TIQ-15. The aspartic acid D97 and arginine R188 were utilized along with the E288 residue in two separate grids: 3ODU-E288-D97 and 3ODU-E288-R188. As for the CVX15 grid (3OE0), the secondary residues were the aspartic acid residue D171 and the arginine R188, utilizing two separate grids: 3OE0-E288-D171 and 3OE0-E288-R188. In total, these four grids were used to analyze binding poses of the three morpholine compounds (**28**, **45,** and **81**). The test of this main interaction, involving a key salt bridge between the THIQ ring nitrogen and the E288 glutamic acid residue, was made in this analysis. Finally, the secondary residues were picked based on the crystal grids themselves, where a secondary or alternative salt bridge may form (D97, D171), or on earlier modeling of TIQ-15 focusing on the unique residue R188, where an interaction was observed in the THQ portion of the molecule. Here, the Schrodinger Maestro suite was utilized using GLIDE in standard precision mode and scoring the top 10 poses. Then, these 10 poses were subjected to optimization using the PRIME function to calculate the binding energy (MMGBSA ΔG_bind_ values) to rank the poses. Although using these scoring functions between noncongeneric series can be inaccurate, this analysis was insightful in providing cross-comparisons between grids. In these cases, the congeneric resemblance between **28**, **45,** and **81** is high and therefore provides the a priori scientific basis for this analysis. We then compiled many of the significant poses and evaluated the best fits. We found there was a common set of poses between the two grids that differed only slightly in positioning and interactions within the receptor.

The poses showed common themes between the grids regarding the conserved portion of the molecules, with slight differences (Figure 4A–B). The key residue interactions are: (i) a pi-cation interaction with the top THQ portion and the R188 residue; (ii) a salt bridge between the THIQ ring nitrogen and the E288 residue; and (iii) a pi-stacking interaction with either the H113, H203, or W94 residues and the phenyl portion of the THIQ ring. An additional weak ionic interaction exists between the residue E262 and the *n*-butyl amine of **28**. A less important interaction is seen between the morpholine ring oxygen atoms of **45** and **81** with the D97 residue, which is not observed for **28**.

**Figure 4:**
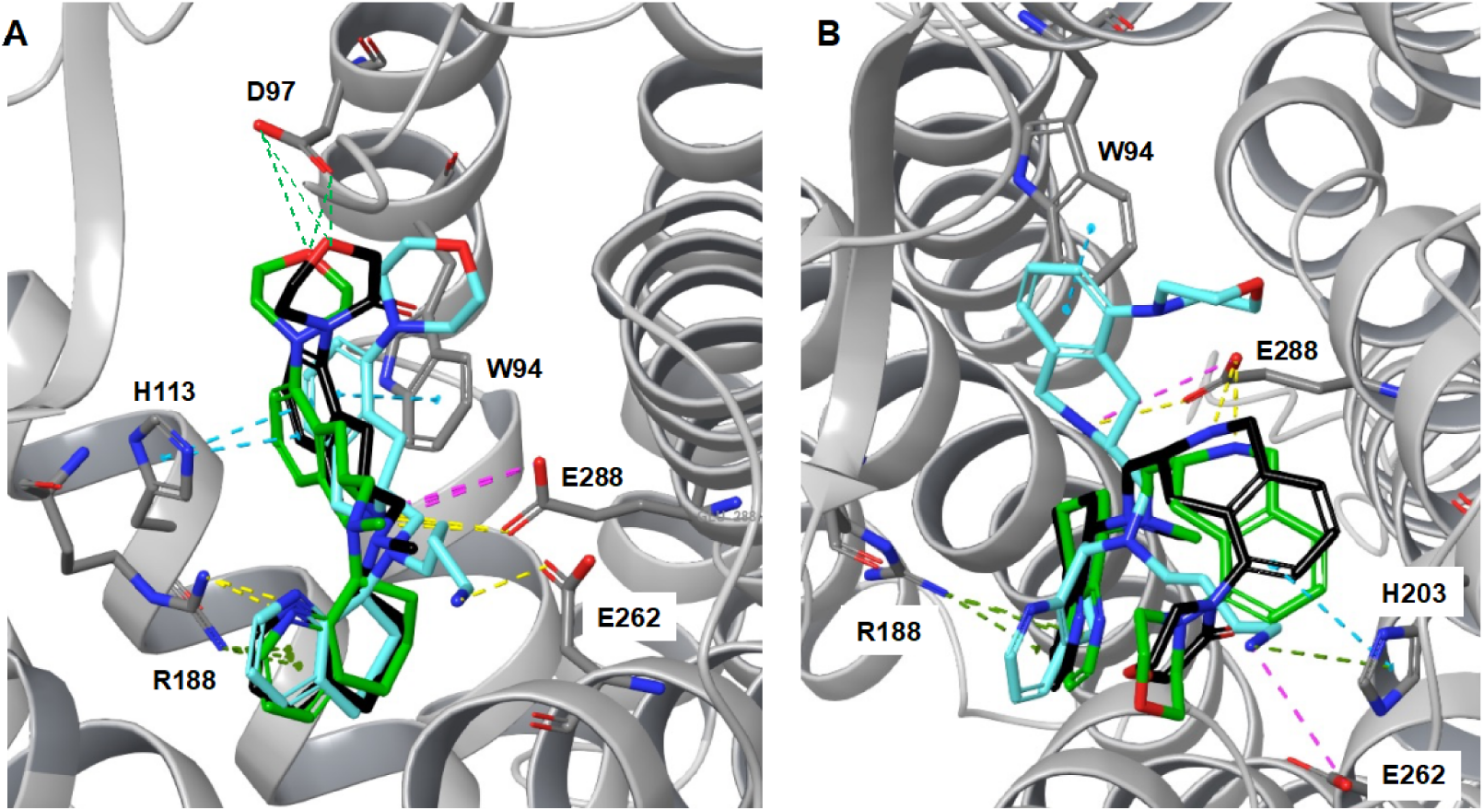
Binding poses for **45** (*green*), **28** (*aqua*) and **81** (*black*) in the CXCR4:IT1t (A, 3ODU) and CXCR4:CVX15 (B, 3OE0) grids.

### Advanced Lead Profiling in ADME and PK Assays

After much medicinal chemistry optimizations described in the Schemes 1 through 5, we identified the best compounds as **28**, **42, 45**, **75,** and **81** for advanced profiling. This was based on a combination of criteria that the combination of CXCR4 potency, CYP inhibition, and metabolic stability, which were equal to or better performing when compared to AMD11070. To differentiate these leads further, we tested the compounds in a number of ADMET evaluations, including permeability and human Ether-à-go-go-Related Gene (hERG) assays (Table 5). First, metabolic half-lives were determined, showing a range of 8–95 min in humans and 5–60 min in mouse species. There was a general trend indicating that the *N*-methyl derivatives (**45**, **75,** and **81**) showed shorter half-lives than the amine-based side chain analogs (**28**, **42**). This could originate from these compounds having lower calculated LogD (cLogD) values, which reflect lower lipophilicity. All these compounds showed better permeability than the predecessor compounds (P_C_ > 100 nm/s; 11070, TIQ-15). The hERG data obtained (either hERG binding or function) showed mixed results. In some cases, the data were better than 11070 (**42**,**75**,**81**) while others (**28**,**45**) indicated potential for QT prolongation, similar to 11070.

**Table 5:**
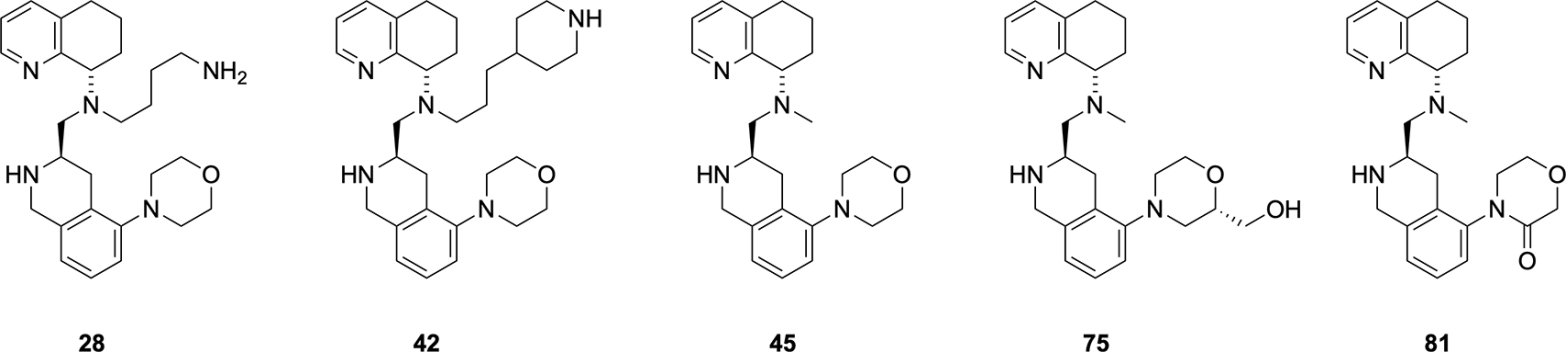
ADMET Assay Results for the Best Leads and Literature Compounds

			Liver Microsomal Stability *T*_1/2_ (min.)					hERG Therapeutic Index (TI)
Comp. No.	CXCR4cAMP IC_50_ (nM)	cLog D^a^ pH_7.4_	HLM	MLM	PAMPA P_*C*_ (nm/s)	hERG ^3^H-Astemizole Binding IC_50_ (nM)	hERG QPatch Functional IC_50_ (nM)	hERG IC_50_/cAMP IC_50_
**11070**	43.9	0.11	19	1.3	86	5,150	6,390	117^b^/146^c^
**TIQ-15**	41.0	–0.84^d^	67	28	0	8,920	-	218^b^
**28**	45.5	–0.99	95	60	228	2,160	-	48^b^
**42**	137	–0.11	20	>30	101	-	18,800	137^c^
**45**	8.25	1.7	7.6	5.4	936	7,640	-	926^b^
**75**	39.3	1.02	21	9.0	138	-	13,100	333^c^
**81**	48.3	0.76	54	38	197	-	77,100	1596^c^

a Calculated using ACD laboratories.

b TI calculated using hERG binding IC_50_.

c TI calculated using hERG functional IC_50_.

d Experimental value for TIQ-15 is −1.0.

Primarily based on the overall properties and good permeability observed for the lead compounds, we decided to determine the pharmacokinetic properties of these compounds in mice (Figure 6, Table 6) We knew that AMD11070 had oral bioavailability in several species, including rats and dogs, but had not seen any mouse results. So, we decided to use it in our studies as a reference compound. First, there was a stark difference in bioavailability between the five compounds in that the morpholine compounds with a methyl side chain (**45**,**75**,**81**) stood out as having better exposure when dosed orally compared to the two amine-based side chains (**28**, **42**) and 11070 (%F = 12–15 vs 0.5–3). When comparing the intravenous dosing studies, the six compounds had differences in clearance and volume of distribution, indicating alternative orders of preference. Given the mouse liver microsomal half-life preferences, the in vivo clearance values show some correlation. However, when comparing the *N*-methyl derivatives, **75** is very similar to **45**, whereas **81** has lower clearance and volume of distribution despite having very similar structures. This was unexpected, as **81** has the highest metabolic stability in the mouse microsomal assay. Additionally, compound **45** has the longest plasma half-life (7.4 h) and C_8h_ levels despite having the lowest half-life in vitro.

**Table 6:** Calculated PK Properties of Selected Morpholine Compounds from Plots in Figure 5

Compound	*C*_max_ (ng/mL)	C_8h_ (ng/mL)	AUC_0–8h_ (h*ng/mL)	Cl (L/h/kg)	V (L/kg)	*T*_1/2_ (h)	F (%)
**11070**	15.7	6.73	42.1	7.22	32.2	5.7	3.0
**28**	3.10	3.90	37.3	1.31	4.48	25	0.49
**42**	6.37	3.11	87.3	2.05	6.03	n.d.	1.8
**45**	84.5	82.0	378	3.74	16.9	7.4	14
**75**	111	7.52	343	4.36	9.07	6.2	15
**81**	409	18.2	1070	1.11	2.33	2.3	12

Figure 5 Further analysis of the PK results was performed by comparing the physicochemical features, mainly the calculated log D values and molecular weight, in a relationship known as the golden triangle developed for evaluating compounds with preferable PK parameters.^54^ Here, log D values were calculated at pH 7.4 for two different software suites and averaged to weight differences in learning sets between the two programs (Table 5). These values were then plotted versus molecular weights and constrained under the defined area of the golden triangle range (Figure 6, designated by the golden triangle backdrop). We found four compounds (**2** (11070), **45**,**75**,**81**) fall inside the triangle, and three (**5** (TIQ-15), **28**, **42**) do not. For the four compounds that are inside, all have positive cLog D_pH7.4_ values determined, while the other three are negative. While TIQ-15 has low permeability as measured by the PAMPA assay (Pc < 10 nm/s), the other six have permeability measured near or above the threshold (Pc > 100 nm/s), which is determined to be the value that corresponds with adequate oral bioavailability (%*F* > 10). The outliers that performed poorly in the PK studies (**28**, **42**) both had higher metabolic stability and adequate permeability, but much lower %F (<2). While the other compounds (**45**, **75**, **81**) with better %F (>10) also had adequate permeability, two of these (**45**, **75**) had lower in vitro metabolic stability (*T*_1/2_ < 10 min.) than the other four, proving a more complex relationship in molecular properties. Here, the golden triangle analysis provided some insight. The two exceptions (**28**, **42**) were rightly predicted to have lower bioavailability (Figure 6A) which seemed to correlate with %F (Figure 6B). Furthermore, the three compounds with better %F values (**45**, **75**, **81**) were rightly predicted to provide adequate exposure (Figure 6A) by falling inside the triangle, and this also correlated with the observed %F (Figure 6B). Therefore, this evaluation can confirm and even predict compounds in this series with better oral bioavailability.

**Figure 5:**
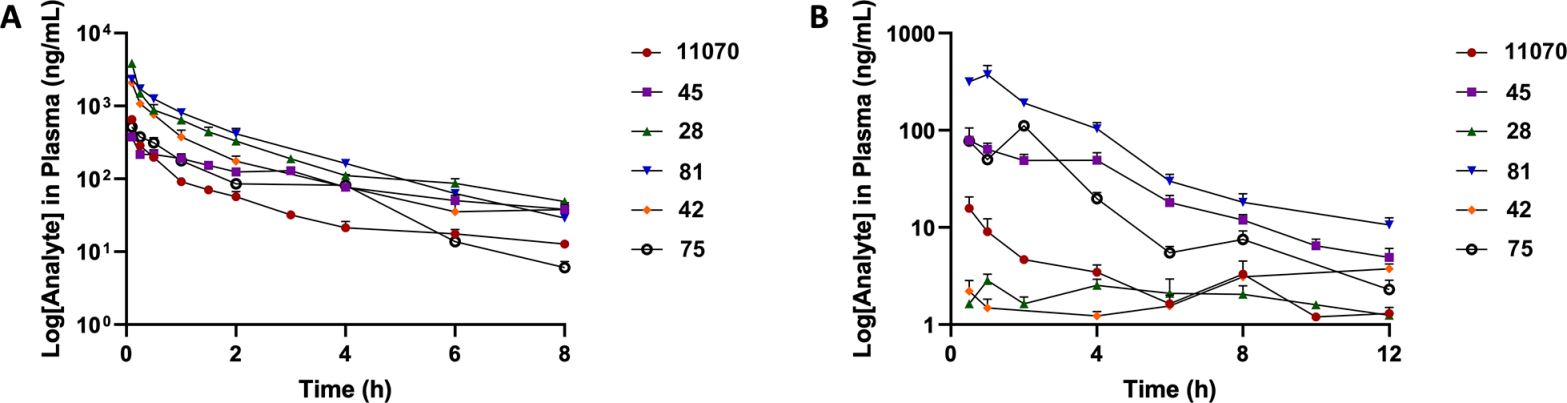
PK plots for compounds in Table 6. (A) Intravenous (IV) dose plots for 3 mg/kg dosing; (B) oral (PO) dose plots for 10 mg/kg dosing.

**Figure 6:**
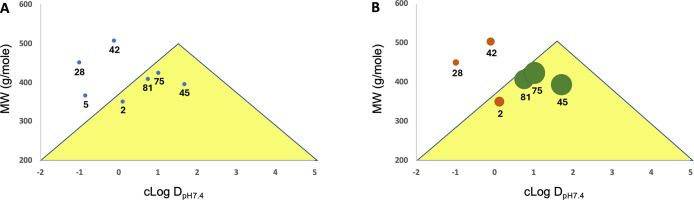
Golden Triangle Plots for compounds in Table 6. (A) Plot of MW versus cLogD_pH7.4_, (B) weighted with %F circles.

## Conclusions

We have presented efforts in studying the medicinal chemistry and pharmacological properties of THIQ-based CXCR4 antagonists, with a main emphasis on altering substituents on the phenyl ring. Although the morpholine ring was discovered serendipitously, the SAR described herein reflects a lead optimization effort. In one direction, changing substituents on the tetrahydroisoquinoline ring of TIQ-15 led to the 2-morpholine substituent and compounds **28** and **42**. Truncating the basic amine side chain was an effort to expand the SAR of **28,** but also an extension of SAR from our earlier 2-piperazine series (compound **6**). The unexpected finding was that the *N*-methyl compound with only one basic nitrogen (**45**) would have similar CXCR4 activity to the compounds with the basic nitrogen side chains (**28**, **42**). Also unexpected was that the SAR of the butyl amine side chain provided very few advances, and that compounds **28** and **42** gave poor oral exposure in a mouse PK experiment despite having good permeability. The removal of these basic centers, along with SAR expansion on compound **45,** gave improved results in the PK studies, which was partially explained in the golden triangle diagram owing to a large calculated log D differential.

Within the study of the two subseries, N-butylamine and *N*-methyl-based compounds with a morpholine ring at the 2-position on the THIQ ring, most (>50%) retained CXCR4 potency in a 9–50 nM range and within 2- to 4-fold of the parent compound TIQ-15. Furthermore, optimization of the compound properties in a series of assays to improve drug properties provided a set of lead compounds with improved properties. This includes diminishing CYP 2D6 inhibition and increasing permeability 10–100-fold over the values observed for TIQ-15. The narrow SAR regarding changes in the morpholine and THQ rings resulted in a lack of improvement in intrinsic CXCR4 activity, CYP 2D6 inhibition, or metabolic stability. Despite this challenge, the best lead compounds were identified, where the *N*-methyl derivatives (**45**, **75**, **81**) provided the best overall properties and can serve as lead compounds for in vivo studies in various models of CXCR4-linked disease. These studies are the goal of future efforts.

## Experimental

### General

All solvents and reagents were purchased from commercial suppliers and used without further purification. Analytical thin-layer chromatography was carried out on silica precoated glass plates Merck KGaA (silica gel 60 F_254_, 0.25 mm thickness) and visualized with UV light at 254 nm and/or with phosphomolybdic acid, or iodine. Automated flash chromatography was performed on a Teledyne ISCO CombiFlash R_f_ 200 system with RediSep R_f_ prepacked silica cartridges (60 Å, 40–63 μm particle size). Concentration refers to rotary evaporation under reduced pressure.

^1^H and ^13^C NMR spectra were recorded on Varian INOVA or VNMR spectrometer operating at 400 or 500 MHz at ambient temperature with CDCl_3_ or MeOH-*d*_4_ as solvents. Data for ^1^H NMR were recorded as follows: δ chemical shift (ppm), multiplicity (s, singlet; d, doublet; dd = doublet of doublet; t, triplet; q, quartet; m, multiplet; br, broad; etc.), coupling constant (Hz), integration. Chemical shifts are reported in parts per million relative to internal reference CDCl_3_ (^1^H NMR: δ 7.26; ^13^C NMR: δ 77.16), MeOH-*d*_4_ (^1^H NMR, δ 4.87, 3.31; ^13^C NMR, δ 49.00), and TMS (^1^H NMR: δ 0.00). Liquid chromatography/mass spectrometry (LCMS) data was obtained to verify molecular mass and analyze purity of products. The specifications of the LCMS instrument are the following: Agilent 1200 HPLC coupled to a 6120 quadrupole mass spectrometer (ESI-API), UV detection at 254 and 210 nm, Agilent Zorbax XDB-18 C_18_ column (50 mm × 4.6 mm, 3.5 μm), gradient mobile phase consisting of MeOH/water/0.1% formic acid buffer, and a flow rate of 1.00 mL/min. The chemical purity of all final compounds was determined by LCMS and confirmed to be ≥95%. High-resolution mass spectra (HRMS) were acquired on a VG 70-S Nier Johnson or JEOL mass spectrometer.

A statement that study samples from mice in the PK studies were obtained by the authority of the institutional board that licensed the use of such material was added to the Supporting Information.

### CXCR4 Calcium Flux Assay

Exemplary compounds were tested for their ability to induce or inhibit calcium flux in CCRF-CEM cells. The experimental procedure and results are provided below. The exemplified biological assays were carried out with all compounds. Human T-lymphoblast cells (CCRF-CEM), expressing endogenous CXCR4 and mAChR, were grown in a suspension culture and plated in clear-bottom 384-well microplates (Greiner bio-one, Cat# 789146) in assay buffer [Hank’s Buffered Saline Solution (Gibco, Cat# 14025–092) supplemented with 20 mM (4-(2-hydroxyethyl)-1-piperazine ethanesulfonic acid (HEPES) (Gibco, Cat# 15630–080) and 0.1% fatty acid-free bovine serum albumin (BSA) (Sigma, Cat# A9205)] at 40,000 cells per well. The cells were loaded with an equal volume of calcium indicator dye (AAT Bioquest Inc., Cat# 34601) for 30 min at 37 °C. The cells were then equilibrated to room temperature for 15 min before the assay. Test compounds were solubilized and serially diluted in DMSO and then transferred to 384-well plates (Matrix, Cat# 4307). The serially diluted compounds were further diluted to their working concentrations with the same assay buffer to 0.5% DMSO. They were added to the cells by the autosampler in the FDSS6000 (Hamamatsu) at final concentrations ranging from 25,000 to 0.423 nM. The activity of the compounds to induce calcium flux was monitored by FDSS in the “agonist mode” for 90 s. For “antagonist mode” assessment, the cells were subsequently incubated for 25 min at room temperature. CXCL12 (R&D System, Cat# 350-NS/CF) or acetylcholine was then added at final concentrations of 5 nM and 2000 nM, respectively, to stimulate the cells. Inhibition of CXCL12- and acetylcholine-induced calcium flux was monitored by FDSS6000 for 90 s. Activation data over a range of concentrations for each test compound was plotted as percent activation of the test compound (100% = maximum response triggered by a saturating concentration of CXCL12, i.e., 160 nM). After correcting for background, the EC_50_ values were determined. EC_50_ is defined as the concentration of the test compound that produces 50% of the maximal response and was quantified using the four-parameter logistic equation to fit the data. The inhibition data for the test compound over a range of concentrations was plotted as percent inhibition of the test compound compared to an internal control compound. IC_50_ is defined as the concentration of the test compound that inhibits 50% of the maximal response and was quantified using the four-parameter logistic equation to fit the data. None of the compounds tested demonstrated agonist activity in the calcium flux assay. All compounds demonstrated EC_50_ values >30 μM. In contrast, the compounds demonstrated a range of potencies in inhibiting CXCL12-induced calcium flux.

### PAMPA Assay

Compounds and controls were utilized as 10 mM stocks in 100% DMSO. The compounds were diluted 1:100 in pH 7.4 or pH 5.5 donor well buffer (pION Cat# 110151), providing a 100 μM assay solution in 1% DMSO. The compounds diluted in the donor well buffer were transferred to a Whatman Unifilter plate and filtered prior to dispensing 200 μL into the donor well of the assay plate (pION Cat# 110163). The PAMPA membrane was formed by pipetting 4 μL of the lipid solution (pION Cat# 110169) onto the filter plate (VWR Cat# 13503). The membrane was then covered with 200 μL of acceptor well buffer at pH 7.4 (pION, Cat# 110139). The PAMPA assay plate (donor side and acceptor side) was combined and allowed to incubate at room temperature for 4 h. The plate was disassembled, and the spectrophotometer plates (VWR, Cat# 655801) were filled (150 μL/well). The donor, acceptor, reference, and blank plates were read in the SpectraMax UV plate reader. Data was captured by the pION software, which analyzed the spectra and generated the PC values.

### CYP Inhibition Assays

The CYP450 (2D6) inhibition assay utilizes enzymes from insect cells expressing the human recombinant CYP450 (2D6) enzyme and the fluorogenic probe (AMMC, 3-[2-(N,N-diethyl-*N*-methylamino)­ethyl]-7-methoxy-4-methylcoumarin), which produces the fluorescent metabolite; both reagents were obtained from Thermo Fisher Scientific/Discovery Labware (Woburn, MA). The assay was performed in a 536-well microplate with a total volume of 5 μL. Automated liquid handling equipment (Thermo Multidrop Combi, LabCyte ECHO 550) was used in every step of compound preparation and for the addition of reagents. Each compound was tested in duplicate at seven concentrations ranging from 1 nM to 20 μM; the final concentration of DMSO in reactions was 0.2%. Positive controls were included in each experiment/run. Test compounds (10 nL/well) were first preincubated at 37 °C for 30 min with 2.5 μL of a prewarmed 2-fold-concentrated mixture of the AMMC fluorogenic substrate (3 μM) and 12.5 nM rCYP2D6 enzyme in 100 mM potassium phosphate assay buffer, pH 7.4. At the end of preincubation, the reactions were initiated by the addition of 2.5 μL of prewarmed 2-fold-concentrated NADPH-regenerating system (16.2 nM NADP) in the same assay buffer. The assay plates were then incubated at 37 °C for 45 min. Immediately after completing the incubation, the reactions were terminated by the addition of 3 μL of quench buffer (80% acetonitrile and 20% 0.5 M TRIS-base). The fluorescence intensity was measured using the Envision fluorescence plate reader (PerkinElmer) at excitation and emission wavelengths of 405 and 460 nm, respectively, using a 430 nm cutoff filter. The endpoint fluorescence readout was normalized to the fluorescence intensity of the reaction performed in the absence of the test substance (totals, 0% inhibition) and the mixture of reaction components in the presence of “Inhibitor Cocktail” (background, 100% inhibition). The IC_50_ value for each compound was derived from the fitted 20-point curve using a four-parameter logistic regression model.

### Mouse Pharmacokinetic Experiments

The study was conducted at Sai Life Sciences Limited, Hyderabad, India, in accordance with the Study Protocol SAIDMPK/PK-21–12–1208. All procedures of the present study were in accordance with the guidelines provided by the Committee for the Purpose of Control and Supervision of Experiments on Animals (CPCSEA), as published in The Gazette of India, December 15, 1998. Prior approval of the Institutional Animal Ethics Committee (IAEC) was obtained before the initiation of the study. The study was conducted as non-GLP; however, all appropriate documentation was maintained in a study file.

### Procedure A

#### General Procedure for Boc Deprotection

To a 20 mL scintillation vial equipped with a Teflon-coated magnetic stir bar was charged Boc-protected substrate (1 equiv) and DCM (0.13 M). TFA (36 equiv) was added dropwise, and the resulting mixture was stirred at room temperature overnight. Upon completion of the reaction, as judged by LCMS analysis, the mixture was diluted with DCM, cooled in an ice bath, and quenched by the addition of 1 M NaOH until pH > 12. The biphasic mixture was transferred to a separatory funnel. The aqueous layer was separated and extracted with DCM (3 times). The combined organic extract was dried over anhydrous sodium sulfate and concentrated under reduced pressure to a crude material, which was purified by a CombiFlash system using a gradient of solvent A (DCM) to solvent B (9:1:0.2 DCM/MeOH/NH_3_ solution, 7N in MeOH, or 8:2:0.6 DCM/MeOH/NH_3_ solution, 7N in MeOH) as eluent on a silica gel column to afford the final product.

### Procedure B

#### General Procedure for Buchwald–Hartwig Coupling

To an oven-dried Biotage 5–10 mL microwave vial equipped with a magnetic stir bar was charged with bromo substituted starting material (1 equiv), Pd_2_(dba)_3_ (0.05 equiv., 5 mol %), *rac*-BINAP (0.15 equiv., 15 mol %), cesium carbonate (1.4 equiv), and amine (if solid) (1.2 equiv). The vial was sealed with a Teflon-lined septum and purged with argon for 5 min. Degassed toluene (0.2 M) and amine (if liquid) (1.2 equiv.), were added successively via a syringe, and the vessel was degassed with argon for another 15 min. The resulting mixture was heated at 120 °C for 24 h. Upon completion of the reaction, as judged by TLC and LCMS, the mixture was allowed to cool to room temperature, filtered through a Celite pad, and concentrated to a crude material, which was purified by a CombiFlash system using a gradient of solvent A (DCM) to solvent B (MeOH or 9:1:0.2 DCM/MeOH/NH_3_ solution, 7N in MeOH) as eluent on a silica column to afford the Boc-protected product.

### Procedure C

#### General Procedure for Reductive Amination

A 20 mL scintillation vial equipped with a magnetic stir bar was charged with amine 17 (1.0 equiv), sodium triacetoxyborohydride (STAB, 1.8 equiv), and 1,2-dichloroethane (1,2-DCE) (0.1 M). After the solution was stirred for 5 min, aldehyde or ketone (1.2–3.0 equiv) was added in one portion. The resulting mixture was stirred at room temperature for 24–72 h. Additional equivalents of aldehyde/ketone might be added to drive the reaction to completion. Upon completion of the reaction, as judged by TLC and LC-MS, the mixture was quenched with 1 N NaOH. The biphasic mixture was transferred to a separatory funnel. The aqueous layer was separated and extracted with DCM three times. The combined organic extract was dried over anhydrous sodium sulfate and concentrated under reduced pressure to yield a crude material, which was purified by a CombiFlash system using a gradient of solvent A (DCM) to solvent B (MeOH or 9:1:0.2 DCM/MeOH/NH_3_ solution, 7N in MeOH) as eluent on a silica column to afford the Boc-protected product.

**Synthesis of *tert*-butyl*(R)*-5-chloro-3-formyl-3,4-dihydroisoquinoline-2­(1** *H* **)-carboxylate (11a)** was done using the same method as for *tert*-butyl 7-chloro-3-formyl-3,4-dihydroisoquinoline-2­(1*H*)-carboxylate (**18c**): steps 1–7 and starting with the corresponding chiral amino acid.

##### *(R)*-2-(*tert*-Butoxycarbonyl)-5-chloro-1,2,3,4-tetrahydroisoquinoline-3-carboxylic acid (**9a**)

^1^H NMR (400 MHz, CDCl_3_): δ 11.62 (s, 1H), 7.27–7.24 (m, 1H), 7.02 (dd, *J =* 13.7, 7.6 Hz, 2H), 5.22 (dd, *J =* 6.3, 2.9 Hz, 0.5H), 4.87 (t, *J =* 5.3 Hz, 0.5H), 4.71 (t, *J =* 16.6 Hz, 1H), 4.45 (t, *J =* 16.8 Hz, 1H), 3.47–2.98 (m, 2H), 1.51 (s, 9H); HRMS calculated for C_15_H_19_ClNO_4_ 312.10021; found 312.10003 [M + H].

##### *tert*-Butyl*(R)*-5-chloro-3-(hydroxymethyl)-3,4-dihydroisoquinoline-2­(1*H*)-carboxylate (**10a**)

^1^H NMR (400 MHz, CDCl_3_): δ 7.25–7.19 (m, 1H), 7.10 (t, *J =* 7.8 Hz, 1H), 7.02–6.96 (m, 1H), 4.75 (d, *J =* 16.9 Hz, 1H), 4.55 (s, 1H), 4.26 (d, *J =* 17.0 Hz, 1H), 3.48 (d, *J =* 6.8 Hz, 2H), 3.02–2.84 (m, 3H), 1.47 (d, *J =* 1.2 Hz, 9H); HRMS calculated for C_15_H_21_ClNO_3_ 298.12108; found 298.12077 [M + H].

##### *tert*-Butyl*(R)*-5-chloro-3-formyl-3,4-dihydroisoquinoline-2­(1H)-carboxylate (**11a**)

^1^H NMR (400 MHz, CDCl_3_): δ 9.51 (d, *J =* 10.4 Hz, 1H), 7.24 (d, *J =* 8.0 Hz, 1H), 7.13 (t, *J =* 8.0 Hz, 1H), 7.05–6.95 (m, 1H), 4.78–4.53 (m, 3H), 3.51–2.96 (m, 2H), 1.52–1.48 (m, 9H); HRMS calculated for C_15_H_19_ClNO_3_ 296.10525; found 296.10502 [M + H].

**Synthesis of *tert*-butyl*(R)*-6-chloro-3-formyl-3,4-dihydroisoquinoline-2­(1*H*)-carboxylate (11b)** was done according to the following procedures.

***(R)*-2-(*tert*-Butoxycarbonyl)-6-chloro-1,2,3,4-tetrahydroisoquinoline-3-carboxylic acid (9b)** was synthesized according to known procedures.^55^

##### *tert*-Butyl*(R)*-6-chloro-3-(hydroxymethyl)-3,4-dihydroisoquinoline-2­(1*H*)-carboxylate (**10b**)

This was synthesized following a similar sequence to that described for *(R)*-*tert*-butyl 7-chloro-3-(hydroxymethyl)-3,4-dihydroisoquinoline-2­(1*H*)-carboxylate (**18c**).

^1^H NMR (400 MHz, CDCl_3_): δ 7.20–6.98 (m, 3H), 4.75–4.37 (m, 2H), 4.25 (d, *J =* 16.9 Hz, 1H), 3.67–3.20 (m, 2H), 3.08–2.75 (m, 3H), 1.47 (s, 9H); HRMS calculated for C_15_H_21_ClNO_3_ 298.12088; found 298.12068 [M + H].

##### *tert*-Butyl*(R)*-6-chloro-3-formyl-3,4-dihydroisoquinoline-2­(1*H*)-carboxylate (**11b**)

The compound was synthesized following a similar sequence described for *(R)*-*tert*-butyl 7-chloro-3-formyl-3,4-dihydroisoquinoline-2­(1*H*)-carboxylate (**18c**).

^1^H NMR (400 MHz, CDCl_3_): δ 9.48 (d, *J =* 15.8 Hz, 1H), 7.15 (d, *J =* 8.3 Hz, 2H), 7.08–6.98 (m, 1H), 4.54–4.45 (m, 2H), 3.11–3.01 (m, 3H), 1.57–1.50 (m, 9H); HRMS calculated for C_15_H_19_ClNO_3_ 296.10532; found 296.10521 [M + H].

**Synthesis of *tert*-butyl (*R*)-2-formyl-1,2-dihydrobenzo­[f]­isoquinoline-3­(4*H*)-carboxylate (11c)** was done from **9c** by the following steps.

##### *(R)*-3-(*tert*-Butoxycarbonyl)-1,2,3,4-tetrahydrobenzo­[f]­isoquinoline-2-carboxylic acid (**9c**)

Step 1.

The suspension of *(R)*-2-amino-3-(naphthalen-1-yl)­propanoic acid **8c** (2.157 g, 10.02 mmol) in HBr (7.94 mL, 70.1 mmol) was heated to 40 °C, and HCHO (1.492 mL, 20.04 mmol) was added. Th mixture was then heated to 80–85 °C. It was stirred for 18 h at 80–85 °C. Then, it was cooled to room temperature and stirred at room temperature overnight to complete the precipitation. The suspension was diluted with toluene and concentrated in vacuo until half of the water was removed. The precipitate was filtered off and dried in vacuo. The crude material was used as it is for the next step.

Step 2.

The *(R)*-1,2,3,4-tetrahydrobenzo­[f]­isoquinoline-2-carboxylic acid hydrobromide **9c** (2.73 g, 8.86 mmol) was suspended in dioxane and 1N NaOH (35.4 mL, 35.4 mmol)­was added. It became a clear solution. Then, di-*tert*-butyl dicarbonate (3.09 mL, 13.29 mmol) was added. A few minutes later it became a suspension again. The reaction mixture was stirred at room temperature overnight. The solvent was evaporated, and 100 mL of ethyl acetate (EA) was added. The pH was adjusted to 2.0 with HCl. The phases were separated, and the aqueous phase was extracted with 50 mL of DCM 4 times. The combined organic layer was dried over anhydrous MgSO_4_ then filtered off and evaporated.

^1^H NMR (400 MHz, CDCl_3_): δ 12.89 (s, 1H), 7.90 (t, *J =* 7.9 Hz, 1H), 7.80–7.74 (m, 1H), 7.63 (dd, *J =* 8.5, 5.2 Hz, 1H), 7.51 (td, *J =* 7.6, 5.3 Hz, 1H), 7.44 (td, *J =* 7.5, 2.7 Hz, 1H), 7.12 (dd, *J =* 8.5, 5.9 Hz, 1H), 5.40–5.34 (m, 0.6H), 5.09 (dd, *J =* 6.7, 2.8 Hz, 0.4H), 4.87–4.73 64 (m, 1H), 4.57 (dd, *J =* 25.4, 17.2 Hz, 1H), 3.90–3.70 (m, 1H), 3.27 (ddd, *J =* 24.0, 16.3, 6.8 Hz, 1H), 1.51 (s, 5H), 1.43 (s, 4H); ^13^C NMR (101 MHz, CDCl_3_) δ 177.26, 155.96, 132.51, 131.82, 130.36, 129.69, 128.71, 127.31, 126.73, 125.77, 124.92, 124.65, 122.90, 81.25, 53.29, 51.75, 44.90, 31.83, 28.63, 26.69.

***(R)*-*tert*-Butyl 3-(hydroxymethyl)-3,4-dihydroisoquinoline-2­(1*H*)-carboxylate (10c)** was synthesized following a similar sequence described for *(R)*-*tert*-butyl 7-chloro-3-(hydroxymethyl)-3,4-dihydroisoquinoline-2­(1*H*)-carboxylate (**18a**).

^1^H NMR (400 MHz, CDCl_3_): δ 7.98–7.90 (m, 1H), 7.82 (dd, *J =* 8.0, 1.5 Hz, 1H), 7.68 (d, *J =* 8.5 Hz, 1H), 7.50 (dddd, *J =* 23.9, 8.1, 6.9, 1.3 Hz, 2H), 7.19 (d, *J =* 8.5 Hz, 1H), 4.83 (d, *J =* 41.7 Hz, 2H), 4.44 (d, *J =* 17.2 Hz, 1H), 3.34–3.13 (m, 2H), 1.60 (s, 3H), 1.51 (s, 9H); ^13^C NMR (101 MHz, CDCl_3_): δ 156.34, 132.63, 132.32, 129.84, 128.81, 127.56, 126.80, 126.58, 125.67, 124.73, 122.95, 80.63, 68.19, 62.72, 50.69, 43.86, 28.73, 25.62.

##### *tert*-Butyl*(R)-*2-formyl-1,2-dihydrobenzo­[f]­isoquinoline-3­(4*H*)-carboxylate (**11c**)

(R)*-tert*-butyl-3-(hydroxymethyl)-3,4-dihydroisoquinoline-2­(1*H*)-carboxylate (0.57 g, 1.8188 mmol) was dissolved in 2.5 mL DCM and cooled to 0 °C. TEA (1.014 mL, 7.2753 mmol) was added to the reaction mixture at 0 °C and stirred for 10 min. Then, pyridine sulfur trioxide (0.8685 g, 5.4565 mmol) in 2.5 mL of DMSO was added dropwise and stirred at 0 °C for 1 h. The reaction was quenched with saturated NH_4_Cl solution (50 mL), followed by the addition of 20 mL of brine and was stirred for 30 min. The aqueous phase was extracted with DCM 2 times. The combined organic layer was evaporated, and the residue was dissolved in EA, washed with brine 3 times, dried over MgSO_4_, filtered off, and evaporated. This was used as is in the next step.

##### (S)–5,6,7,8-Tetrahydroquinolin-8-amine (**12**)

(T) −5,6,7,8-Tetrahydroquinolin-8-amine was prepared according to reference.^56^

##### *tert*-Butyl*(R)*-5-chloro-3-(((*(S)*-5,6,7,8-tetrahydroquinolin-8-yl)­amino)­methyl)-3,4-dihydroisoquinoline-2­(1*H*)-carboxylate (**13a**)

Procedure C was used, starting with *tert*-butyl *(R)*-5-chloro-3-formyl-3,4-dihydroisoquinoline-2­(1*H*)-carboxylate (**11a**) and *(S)*-5,6,7,8-tetrahydroquinolin-8-amine (**12**). The crude material was used for the next step without further purification.

##### *tert-*Butyl*(R)*-6-chloro-3-(((*(S)*-5,6,7,8-tetrahydroquinolin-8-yl)­amino)­methyl)-3,4-dihydroisoquinoline-2­(1*H*)-carboxylate (**13b**)

Procedure C was used, starting with *tert*-butyl-*(R)*-6-chloro-3-formyl-3,4-dihydroisoquinoline-2­(1*H*)-carboxylate (**11b**) and *(S)*-5,6,7,8-tetrahydroquinolin-8-amine (**12**). The crude material was used for the next step without further purification.

^1^H NMR (400 MHz, CDCl_3_): δ 8.34 (s, 1H), 7.34 (d, *J =* 7.7 Hz, 1H), 7.14–7.11 (m, 2H), 7.03–6.98 (m, 2H), 4.75–4.53 (m, 2H), 4.21 (d, *J =* 17.1 Hz, 1H), 3.69 (d, *J =* 0.4 Hz, 1H), 3.06–2.76 (m, 3H), 2.76–2.45 (m, 4H), 1.74–1.57 (m, 12H), 1.49 (d, *J =* 6.0 Hz, 9H).

##### *tert*-Butyl*(R)*-2-(((*(S)*-5,6,7,8-tetrahydroquinolin-8yl)­amino)­methyl)-1,2-dihydrobenzo­[f]­isoquinoline-3­(4*H*)-carboxylate (**13c**)

Procedure C was used, starting with *(R)*-*tert*-butyl-2-formyl-1,2-dihydrobenzo­[f]­isoquinoline-3­(4*H*)-carboxylate (**11c**) and *(S)*-5,6,7,8-tetrahydroquinolin-8-amine (**12**). It was purified with column chromatography, starting with DCM and increasing the polarity with DCM:MeOH:NH_3_(in MeOH solution) (90:10:2), to give a 75% yield of yellowish foam.

^1^H NMR (400 MHz, CDCl_3_): δ 8.33 (dd, *J =* 4.8, 1.7 Hz, 1H), 7.97 (d, *J =* 8.4 Hz, 1H), 7.80 (d, *J =* 8.0 Hz, 1H), 7.67 (d, *J =* 8.5 Hz, 1H), 7.48 (dddd, *J =* 22.9, 8.0, 6.8, 1.3 Hz, 2H), 7.31 (d, *J =* 7.2 Hz, 1H), 7.18 (d, *J =* 8.6 Hz, 1H), 7.02 (dd, *J =* 7.7, 4.7 Hz, 1H), 5.13–4.69 (m, 2H), 4.49–4.31 (m, 1H), 3.85–3.41 (m, 3H), 3.16 (dd, *J =* 16.6, 6.2 Hz, 1H), 2.85 (dd, *J =* 11.7, 7.1 Hz, 1H), 2.77–2.61 (m, 2H), 1.93–1.80 (m, 2H), 1.52 (s, 9H); ^13^C NMR (101 MHz, CDCl_3_): δ 157.50, 155.40, 147.00, 137.04, 132.59, 132.55, 128.74, 126.60, 126.41, 125.53, 124.81, 124.74, 123.09, 122.96, 122.05, 80.26, 57.91, 53.71, 50.16, 48.75, 28.94, 28.74, 27.37, 26.97, 19.62; LC-MS (ESI-API, 254 nm) 75–95% MeOH in H_2_O (0.1% HCO_2_H), 3 min, 1.00 mL/min, C18 (Agilent Zorbax XDB-18, 50 mm × 4.6 mm, 3.5 μm), *m*/*z* = 444.2 (M + 1), t = 0.460 min, purity: > 95%; HRMS calculated for C_28_H_34_O_2_N_3_ 444.26455; found 444.26442 [M + H].

***tert*-Butyl (*tert*-butoxycarbonyl)­(4-oxobutyl)­carbamate (14)** was synthesized according to published procedures.^57^

**N^1^-((*(R)*-5-Chloro-1,2,3,4-tetrahydroisoquinolin-3-yl)­methyl)-N^1^-(*(S)*-5,6,7,8-tetrahydroquinolin-8-yl)­butane-1,4-diamine (15)** was synthesized by the following steps.

Step 1.

##### *tert*-Butyl*(R)*-3-(((4-(bis­(*tert*-butoxycarbonyl)­amino)­butyl)­(*(S)*-5,6,7,8-tetrahydroquinolin-8-yl)­amino)­methyl)-5-chloro-3,4-dihydroisoquinoline-2­(1*H*)-carboxylate

Procedure C was used, starting with *tert*-butyl-*(R)*-5-chloro-3-(((*(S)*-5,6,7,8-tetrahydroquinolin-8-yl)­amino)­methyl)-3,4-dihydroisoquinoline-2­(1*H*)-carboxylate (**13a**) and *tert*-butyl (*tert*-butoxycarbonyl)­(4-oxobutyl)­carbamate (**14**). The crude material was purified by the CombiFlash system starting with DCM and increasing the polarity with a DCM/MeOH/NH_3_ (7N in MeOH) solution (90:10:2), to afford the title compound in 26% yield as a yellow foam.

^1^H NMR (400 MHz, CDCl_3_): δ 8.42 (d, *J =* 13.3 Hz, 1H), 7.37 (d, *J =* 7.7 Hz, 1H), 7.09 (dd, *J =* 8.0, 2.2 Hz, 1H), 6.94–6.79 (m, 3H), 5.45–4.93 (m, 1H), 4.53 (d, *J =* 16.8 Hz, 1H), 4.29 (s, 1H), 4.08–3.85 (m, 1H), 3.77 (d, *J =* 32.4 Hz, 1H), 3.65–3.37 (m, 7H), 3.37–3.04 (m, 1H), 3.05–2.69 (m, 1H), 2.69–2.39 (m, 2H), 2.39–2.20 (m, 1H), 2.10–1.62 (m, 3H), 1.60–1.46 (m, 3H), 1.41 (s, 18H), 1.38 (s, 9H).

Step 2.

The step 1 intermediate was used with Procedure A to give **15** as a white foam in a 63% yield.

^1^H NMR (400 MHz, CDCl_3_): δ 8.51 (d, *J =* 4.7 Hz, 1H), 7.36 (d, *J =* 7.7 Hz, 1H), 7.19 (s, 1H), 7.12–7.00 (m, 2H), 6.95 (d, *J =* 7.7 Hz, 1H), 4.19–3.98 (m, 2H), 3.88–3.53 (m, 4H), 3.13–2.99 (m, 2H), 2.91–2.58 (m, 7H), 2.46 (dd, *J =* 13.3, 10.1 Hz, 1H), 2.29–1.87 (m, 4H), 1.79–1.48 (m, 5H); ^13^C NMR (101 MHz, CDCl_3_): δ 158.1, 146.5, 137.9, 136.5, 134.2, 133.7, 132.5, 126.4, 126.2, 124.7, 121.4, 61.4, 57.9, 54.2, 52.1, 48.5, 41.3, 31.8, 30.1, 29.2, 27.7, 26.9, 21.8; HRMS calculated for C_23_H_32_ClN_4_ 399.23155; found 399.23072 [M + H].

##### N^1^-((*(R)*-6-Chloro-1,2,3,4-tetrahydroisoquinolin-3-yl)­methyl)-N^1^-(*(S)*-5,6,7,8-tetrahydroquinolin-8-yl)­butane-1,4-diamine (**16**)

Compound **16** was synthesized according to the following steps.

Step 1.

##### *tert*-Butyl*(R)*-3-(((4-(bis­(*tert*-butoxycarbonyl)­amino)­butyl)­(*(S)*-5,6,7,8-tetrahydroquinolin-8-yl)­amino)­methyl)-6-chloro-3,4-dihydroisoquinoline-2­(1*H*)-carboxylate

Procedure C was used, starting with *(R)*-*tert*-butyl 6-chloro-3-(((*(S)*-5,6,7,8-tetrahydroquinolin-8-yl)­amino)­methyl)-3,4-dihydroisoquinoline-2­(1*H*)-carboxylate (**13b**) and *tert*-butyl (*tert*-butoxycarbonyl)­(4-oxobutyl)­carbamate (**14**). The crude material was used for the next step without further purification.

Step 2.

The Step 1 intermediate was used with the same procedure and resulted in a slightly yellow gum (46% yield).

^1^H NMR (400 MHz, CDCl_3_): δ 8.42 (dd, *J =* 4.6, 1.4 Hz, 1H), 7.30 (dd, *J =* 7.6, 1.4 Hz, 1H), 7.04–7.00 (m, 3H), 6.91 (d, *J =* 8.0 Hz, 1H), 4.05 (dd, *J =* 9.9, 6.3 Hz, 1H), 3.99 (d, *J =* 15.4 Hz, 1H), 3.80 (d, *J =* 15.1 Hz, 1H), 3.01 (dd, *J =* 7.4, 5.5 Hz, 1H), 2.96 (dd, *J =* 13.2, 3.0 Hz, 1H), 2.77–2.63 (m, 5H), 2.62–2.51 (m, 2H), 2.37 (d, *J =* 11.2 Hz, 1H), 2.32 (dd, *J =* 13.2, 10.4 Hz, 1H), 2.16 (bs, 3NH), 2.08–2.02 (m, 2H), 1.99–1.66 (m, 3H), 1.57–1.38 (m,4H); ^13^C NMR (100 MHz, CDCl_3_): δ = 158.6, 146.7, 136.7, 136.6, 134.2, 134.0, 131.3, 128.8, 127.8, 125.7, 121.5, 61.3, 57.7, 54.3, 52.0, 48.3, 42.1, 33.7, 31.4, 29.4, 29.0, 27.3, 22.0; LC/MS 75–95% MeOH in H_2_O over 3 min, r_*t*_ = 0.748 at 254 nM, MS (+) 399.2, MS­(+)/2 200.2; HRMS calculated for C_23_H_32_N_4_Cl 399.23100; found 399.23070 [M + H].

**N^1^-((*(R)*-1,2,3,4-Tetrahydrobenzo­[f]­isoquinolin-2-yl)­methyl)-N^1^-(*(S)*-5,6,7,8-tetrahydroquinolin-8-yl)­butane-1,4-diamine (17)** was synthesized according to the following steps.

Step 1.

##### *tert*-Butyl*(R)*-2-(((4-(bis­(*tert*-butoxycarbonyl)­amino)­butyl)­(*(S)*-5,6,7,8-tetrahydroquinolin-8-yl)­amino)­methyl)-1,4-dihydrobenzo­[f]­isoquinoline-3­(2*H*)-carboxylate

Procedure C was used, starting with *(R)*-*tert*-butyl-2-(((*(S)*-5,6,7,8-tetrahydroquinolin-8-yl)­amino)­methyl)-1,2-dihydrobenzo­[f]­isoquinoline-3­(4*H*)-carboxylate (**13c**) and (*tert*-butoxycarbonyl)­(4-oxobutyl)­carbamate (**14**). It was used without purification for the next step.

Step 2. The Step 1 intermediate was used with the same procedure, providing an off-white foam (75% yield).

^1^H NMR (400 MHz, CDCl_3_): δ 8.46 (dd, *J =* 4.8, 1.7 Hz, 1H), 7.83 (d, *J =* 8.3 Hz, 1H), 7.74 (d, *J =* 8.3 Hz,1H), 7.58 (d, *J =* 8.4 Hz, 1H), 7.47–7.35 (m, 2H), 7.30 (d, *J =* 7.7 Hz, 1H), 7.11 (d, *J =* 8.4 Hz, 1H), 7.03 (dd, *J =* 7.7, 4.8 Hz, 1H), 4.20–3.97 (m, 3H), 3.19–2.93 (m, 3H), 2.87–2.41 (m, 9H), 2.15–2.02 (m, 1H), 2.02–1.84 (m, 2H), 1.78–1.63 (m, 1H), 1.62–1.39 (m, 4H); ^13^C NMR (101 MHz, CDCl_3_) δ 158.91, 146.97, 136.78, 134.21, 133.12, 132.54, 132.36, 129.71, 128.58, 126.07, 125.49, 125.10, 122.86, 121.67, 61.59, 58.34, 54.66, 52.39, 49.48, 42.25, 31.48, 30.83, 29.68, 29.13, 27.46, 22.23; LC-MS (ESI-API, 254 nm) 75% MeOH in H_2_O (0.1% HCO_2_H), 3 min, 1.00 mL/min, C18 (Agilent Zorbax XDB-18, 50 mm × 4.6 mm, 3.5 μm), *m*/*z* = 415.2 (M + 1), t = 0.887 min, purity: **≥** 95%; HRMS calculated for C_27_H_35_N_4_ 415.28529; found 415.28562 [M + H].

##### Synthesis of *tert*-Butyl *(R)*-6-chloro-3-formyl-3,4-dihydroisoquinoline-2­(1*H*)-carboxylate (**18c**)

Step 1. **Methyl 2-amino-3-(4-chlorophenyl)­propanoate.**

A suspension of 2-amino-3-(4-chlorophenyl)­propanoic acid (2.50 g, 12.52 mmol) in anhydrous MeOH (20 mL) was cooled in an ice–water bath and carefully treated with thionyl chloride (1 mL, 13.71 mmol). After stirring for 10 min, the cooling bath was removed, and the mixture was allowed to warm to room temperature. A reflux condenser was attached, and the slurry was warmed to 55 °C. After stirring overnight, the mixture was cooled to room temperature, concentrated, and dried under reduced pressure to give the title compound as a white solid (3.15 g, 12.59 mmol, quantitative yield).

^1^H NMR (400 MHz, (CD_3_)_2_SO): δ 8.87 (s, 3H), 7.53–7.11 (m, 4H), 4.23 (dd, *J =* 7.3, 5.6 Hz, 1H), 3.66 (s, 3H), 3.35–3.00 (m, 2H); HRMS calculated for C_10_H_13_ClNO_2_ 214.06348; found 214.06291 [M + H].

Step 2. **Methyl 3-(4-chlorophenyl)-2-((ethoxycarbonyl)­amino)­propanoate.**

A stirred mixture of methyl 2-amino-3-(4-chlorophenyl)­propanoate HCl salt (3.13 g, 12.51 mmol) in DCM (42 mL) was cooled in an ice–water bath and carefully treated with pyridine (2.23 mL, 27.6 mmol) and ethyl chloroformate (1.27 mL, 13.28 mmol). After stirring for 1 h, the solution was diluted with EA and water, and the layers were separated. The aqueous phase was re-extracted with EA (2 × 50 mL), and the combined organic phase was washed with saturated aqueous sodium chloride, dried over Na_2_SO_4_, and concentrated under reduced pressure to give the title compound as a white solid (3.60 g, 12.60 mmol, quantitative yield).

^1^H NMR (400 MHz, CDCl_3_): δ 7.28–7.08 (m, 2H), 7.08–6.89 (m, 2H), 5.11 (s, 1H), 4.55 (q, *J =* 6.5 Hz, 1H), 4.03 (qd, *J =* 7.1, 1.5 Hz, 2H), 3.65 (d, *J =* 1.5 Hz, 3H), 3.00 (qd, *J =* 14.0, 5.9 Hz, 2H), 1.30–1.00 (m, 3H); HRMS calculated for C_13_H_17_ClNO_4_ 286.0846; found 286.08401 [M + H].

Step 3. **2-Ethyl-3-methyl-7-chloro-3,4-dihydroisoquinoline-2,3­(1** ***H*** **)-dicarboxylate.**

A mixture of methyl 3-(4-chlorophenyl)-2-((ethoxycarbonyl)­amino)­propanoate (6.51 g, 22.78 mmol) in AcOH (22 mL) and H_2_SO_4_ (7.5 mL) was treated with paraformaldehyde (0.72 g, 23.98 mmol). After stirring at room temperature overnight, the mixture was added to ice, diluted with water, and extracted with EA (3 × 50 mL). The combined organic phase was washed with saturated aqueous sodium chloride, dried over MgSO_4_, and concentrated. Purification by silica gel chromatography (25–50% EA in hexanes) gave the title compound as a clear, viscous oil (4.5231 g, 15.19 mmol, 66.7% yield).

^1^H NMR (400 MHz, CDCl_3_): δ 7.24–7.16 (m, 0.25H), 7.11–6.92 (m, 2.61H), 5.11 (dd, *J =* 6.2, 2.9 Hz, 0.51H), 4.89 (dd, *J =* 6.0, 3.8 Hz, 0.36H), 4.68 (dd, *J =* 16.7, 5.5 Hz, 1H), 4.54–4.36 (m, 1H), 4.29–4.06 (m, 2H), 3.56 (d, *J =* 0.8 Hz, 3H), 3.27–2.89 (m, 2H), 1.22 (dt, *J =* 29.7, 7.1 Hz, 3H); HRMS calculated for C_14_H_17_ClNO_4_ 298.08461; found 298.08405 [M + H].

Step 4. **7-Chloro-1,2,3,4-tetrahydroisoquinoline-3-carboxylic acid.**

A mixture of 2-ethyl-3-methyl-7-chloro-3,4-dihydroisoquinoline-2,3­(1*H*)-dicarboxylate (2 g, 6.72 mmol) and LiOH-H_2_O (1.691 g, 40.3 mmol) in water (11 mL) was heated for 1 h at 180 °C using microwave irradiation. The mixture was cooled and acidified to pH 5. The white solid was filtered, dried in vacuo, and used in the next step without further purification. HRMS calculated for C_10_H_11_ClNO_2_ 212.04783; found 212.04732 [M + H].

Step 5. **2-(** ***tert*** **-Butoxycarbonyl)-7-chloro-1,2,3,4-tetrahydroisoquinoline-3-carboxylic acid (18a).**

A solution of 7-chloro-1,2,3,4-tetrahydroisoquinoline-3-carboxylic acid (**8d**) (1 g, 4.72 mmol) in 1,4-dioxane (10 mL) and 1 M aqueous NaOH (14.17 mL, 14.17 mmol) was cooled in an ice–water bath and treated with a second solution of di-*tert*-butyl dicarbonate (1.547 g, 7.09 mmol) in 1,4-dioxane (5 mL). After 15 min, the cooling bath was removed, and the stirring was continued overnight. The resulting mixture was diluted with water and washed with EA. The aqueous phase was adjusted to pH ∼3 with 1 M HCl and extracted with EA (2 × 50 mL). The combined organic phase was washed with saturated aqueous sodium chloride, dried over MgSO_4_, and concentrated under reduced pressure. The residue was purified by silica gel chromatography (25–75% EA in hexanes) to give the title compound as a white solid (1.2914 g, 4.14 mmol, 88% yield).

^1^H NMR (400 MHz, CDCl_3_): δ 11.62 (s, 1H), 7.21–6.94 (m, 3H), 5.13 (dd, *J =* 6.3, 2.9 Hz, 0.5H), 4.77 (t, *J =* 5.3 Hz, 0.5H), 4.63 (d, *J =* 16.6 Hz, 1H), 4.43 (t, *J =* 16.8 Hz, 1H), 3.37–2.83 (m, 2H), 1.50 (s, 9H); HRMS calculated for C_15_H_19_ClNO_4_ 312.10026; found 312.10005 [M + H].

Step 6. ***tert*** **-Butyl 7-chloro-3-(hydroxymethyl)-3,4-dihydroisoquinoline-2­(1** *H* **)-carboxylate (18b).**

A solution of 2-(*tert*-butoxycarbonyl)-7-chloro-1,2,3,4-tetrahydroisoquinoline-3-carboxylic acid (**18a**) (1 g, 3.21 mmol) in anhydrous tetrahydrofuran (16 mL) was cooled in an ice bath and carefully treated with a solution of borane in dimethyl sulfide (1.187 mL, 11.87 mmol). The resulting mixture was stirred at 0 °C for 1.5 h and then at room temperature overnight. The mixture was then cooled in an ice–water bath, quenched slowly by the dropwise addition of water until most gas evolution ceased, diluted with additional water, and extracted with EA (2 × 25 mL). The combined organic phase was washed with saturated aqueous NaHCO_3_, and saturated aqueous sodium chloride, dried over Na_2_SO_4_, and concentrated under reduced pressure. Purification by silica gel chromatography (10–20% EA in methylene chloride) gave the title compound as a clear gum (1.1236 g, 3.77 mmol, quantitative yield).

^1^H NMR (400 MHz, CDCl_3_): δ 7.19–6.98 (m, 3H), 4.82–4.37 (m, 2H), 4.23 (d, *J =* 16.9 Hz, 1H), 3.67–3.24 (m, 2H), 3.10–2.87 (m, 2H), 2.78 (dd, *J =* 16.2, 3.0 Hz, 1H), 1.47 (s, 9H); HRMS calculated for C_15_H_21_ClNO_3_ 298.12100; found 298.12073 [M + H].

Step 7. ***tert*** **-Butyl 7-chloro-3-formyl-3,4-dihydroisoquinoline-2­(1** *H* **)-carboxylate (18c).**

A stirred solution of *tert*-butyl 7-chloro-3-(hydroxymethyl)-3,4-dihydroisoquinoline-2­(1*H*)-carboxylate (**18b**) (1.06 g, 3.56 mmol) in methylene chloride (35.6 mL) was cooled in an ice–water bath and treated dropwise with a solution of Dess–Martin periodinane (1.812 g, 4.27 mmol) in methylene chloride over several minutes. The cooling bath was removed, and the resulting mixture was stirred at room temperature overnight. Once complete, the mixture was recooled in an ice–water bath, quenched with a 1:1 mixture of saturated aqueous Na_2_S_2_O_3_ and saturated aqueous NaHCO_3_, and stirred for 10 min at room temperature. The layers were separated, and the aqueous phase was reextracted with methylene chloride. The combined organic phase was concentrated under reduced pressure. Purification by silica gel chromatography (10–30% EA in hexanes) gave the title compound as a clear gum (0.6702 g, 2.266 mmol, 64% yield).

^1^H NMR (400 MHz, CDCl_3_): δ 9.45 (d, *J =* 10.4 Hz, 1H), 7.20–6.86 (m, 3H), 4.98–4.16 (m, 3H), 3.48–2.71 (m, 2H), 1.66–1.30 (m, 9H); HRMS calculated for C_15_H_19_ClNO_3_ 296.10535; found 296.10512 [M + H].

##### *tert*-Butyl 5-bromo-3-formyl-3,4-dihydroisoquinoline-2­(1*H*)-carboxylate (**19b**)

Compounds **19a**–**b** and **20a**–**b**, were prepared according to known procedures.^50^

##### 2-(*tert*-Butyl)-3-methyl-*(R)*-5-morpholino-3,4-dihydroisoquinoline-2,3­(1*H*)-dicarboxylate (**20c**)

Procedure B was used, starting with 2-(*tert*-butyl)-3-methyl-*(R)*-5-bromo-3,4-dihydroisoquinoline-2,3­(1*H*)-dicarboxylate (**19a**).^50^ The residue was then subjected to column chromatography using a hexanes/EA gradient to give a white foam (64% yield).

^1^H NMR (400 MHz, CDCl_3_): δ 1.42 (s, 5H), 1.49 (s, 4H), 2.85 (m, 5H), 3.1 (dd, 0.5 H, *J =* 6 Hz, *J =* 16 Hz), 3.22 (dd, 0.5H, *J =* 6 Hz, *J =* 16 Hz), 3.5 (dd, 0.5H, *J =* 3 Hz, *J =* 16 Hz), 3.57 (s, 1.5H), 3.62 (s, 1.5H), 3.83 (m, 4H), 4.43 (dd, 1H, *J =* 16 Hz, *J =* 34 Hz), 4.67 (dd, 1H, *J =* 16 Hz, *J =* 34 Hz), 4.64 (d, 0.5H, *J =* 11 Hz), 5.04 (dd, 0.5H, *J =* 4 Hz, *J =* 6 Hz), 6.84 (d, 0.5H, *J =* 7 Hz), 6.91 (t, 1.5H, *J =* 8 Hz), 7.15 (t, 0.5H, *J =* 8 Hz), 7.162 (t, 0.5H, *J =* 8 Hz).

##### *tert*-Butyl*(R)*-3-formyl-5-morpholino-3,4-dihydroisoquinoline-2­(1*H*)-carboxylate (**20d**)

A solution of 0.444 g of 2-(*tert*-butyl)-3-methyl-*(R)*-5-morpholino-3,4-dihydroisoquinoline-2,3­(1*H*)-dicarboxylate (**20c**) in 10 mL of anhydrous toluene was cooled to −78 °C, and then 3 mL of a 1 M diisobutyl aluminum hydride/toluene solution was added slowly over a 30-min period. The reaction was stirred for an additional 3 h at −78 °C. Then, an additional 3 mL of the DIBAL-H solution was added, and the reaction was stirred for an additional 30 min. The reaction was then quenched by the addition of 12 mL of EA, 6 mL of acetone, and 6 mL of MeOH. The reaction was then warmed to room temperature, and 50 mL of NH_4_Cl (aq.) solution was added, followed by stirring overnight. The reaction was then extracted with additional EA, and the organic layers were separated and dried over anhydrous Na_2_SO_4_. Filtration and solvent removal gave a yellow viscous residue, which was subjected to column chromatography (ISCO, 1 2g column, hexanes: EA gradient) to provide 0.315 g (77% yield) of a clear viscous oil.

^1^H NMR (400 MHz, CDCl_3_): δ 9.43 (d, 1H, J = 8 Hz), 7.18 (q, 1H, J = 7 Hz), 6.93 (t, 1H, J = 12 Hz), 6.85 (m, 1H), 4.76 (t, 0.5H, J = 6 Hz), 4.64 (t, 1H, J = 15 Hz), 4.50 (d, 1H, J = 16 Hz), 4.39 (t, 0.5H, J = 6 Hz), 3.85 (m, 4H), 3.47 (dd, 0.5H, J = 6, 16 Hz), 3.26 (dd, 0.5H, J = 6, 16 Hz), 2.97 (m, 1H), 2.90 (m, 2H), 2.85 (m, 2H), 1.51 (s, 5H), 1.43 (s, 4H).

##### *tert*-Butyl 5-bromo-3-(((*(S)*-5,6,7,8-tetrahydroquinolin-8-yl)­amino)­methyl)-3,4-dihydroisoquinoline-2­(1*H*)-carboxylate (**21a**)

Procedure C was used, starting with *tert*-butyl 5-bromo-3-formyl-3,4-dihydroisoquinoline-2­(1*H*)-carboxylate (**19b**) and *(S)*-5,6,7,8-tetrahydroquinolin-8-amine (**12**). The product was purified with column chromatography, starting with DCM and increasing the polarity with DCM:MeOH:NH_4_OH (90:10:1) in 56% yield as a white foam.

^1^H NMR (400 MHz, CDCl_3_): δ 8.38–8.30 (m, 1H), 7.42 (dd, *J =* 6.2, 3.0 Hz, 1H), 7.33 (d, *J =* 7.6 Hz, 1H), 7.06–7.02 (m, 3H), 4.74 (d, *J =* 41.8 Hz, 1H), 4.24 (s, 1H), 3.75–3.60 (m, 1H), 3.13 (d, *J =* 17.7 Hz, 1H), 2.91–2.58 (m, 5H), 2.01–1.83 (m, 3H), 1.76–1.58 (m, 3H), 1.48 (s, 9H).

##### *tert*-Butyl 7-chloro-3-(((*(S)*-5,6,7,8-tetrahydroquinolin-8-yl)­amino)­methyl)-3,4-dihydroisoquinoline-2­(1*H*)-carboxylate (**21b**)

Procedure C was used, starting with *(R)*-*tert*-butyl 7-chloro-3-formyl-3,4-dihydroisoquinoline-2­(1*H*)-carboxylate (**18c**) and *(S)*-5,6,7,8-tetrahydroquinolin-8-amine (**12**). The crude material, which was purified by the CombiFlash system, starting with DCM and increasing the polarity with DCM/MeOH/NH_3_ (7N in MeOH) (90:10:2) to afford the title compound as a yellow amorphous solid in quantitative yield.

^1^H NMR (400 MHz, CDCl_3_): δ 8.45–8.25 (m, 1H), 7.42–7.30 (m, 1H), 7.20–6.98 (m, 4H), 4.65 (s, 2H), 4.22 (d, *J =* 16.8 Hz, 1H), 3.80–3.57 (m, 1H), 3.12–2.50 (m, 7H), 2.09–1.89 (m, 2H), 1.46 (d, *J =* 27.3 Hz, 11H).

##### *tert-*Butyl 5-(4-(*tert*-butoxycarbonyl)­piperazin-1-yl)-3-(((*(S)*-5,6,7,8-tetrahydroquinolin-8-yl)­amino)­methyl)-3,4-dihydroisoquinoline-2­(1*H*)-carboxylate (**21c**)

Procedure C was used, starting with *tert*-butyl 5-(4-(*tert*-butoxycarbonyl)­piperazin-1-yl)-3-formyl-3,4-dihydroisoquinoline-2­(1*H*)-carboxylate (**20b**)^50^ and *(S)*-5,6,7,8-tetrahydroquinolin-8-amine (**12**). The crude material was purified by using the CombiFlash system starting with DCM and increasing the polarity with MeOH to 10% MeOH/DCM, to afford the title compound in 61% yield as a yellow amorphous solid.

^1^H NMR (400 MHz, CDCl_3_): δ 8.36–8.29 (m, 1H), 7.35 (d, *J =* 7.5 Hz, 1H), 7.16 (t, *J =* 7.8 Hz, 1H), 7.09–7.01 (m, 1H), 6.93–6.79 (m, 2H), 4.67 (d, *J =* 16.8 Hz, 2H), 4.32 (d, *J =* 16.5 Hz, 1H), 3.86–3.22 (m, 6H), 2.95 (s, 2H), 2.85–2.61 (m, 6H), 2.59–2.24 (m, 2H), 1.95 (s, 2H), 1.65 (d, *J =* 8.7 Hz, 2H), 1.49 (d, *J =* 8.8 Hz, 18H); HRMS calculated for C_33_H_48_N_5_O_4_ 578.37063; found 578.36923 [M + H].

##### *tert*-Butyl*(R)*-5-morpholino-3-(((*(S)*-5,6,7,8-tetrahydroquinolin-8-yl)­amino)­methyl)-3,4-dihydroisoquinoline-2­(1*H*)-carboxylate (**21d**)

Procedure C was used, starting with *(S)*-5,6,7,8-tetrahydroquinolin-8-amine (**12**) and *tert*-butyl-*(R)*-3-formyl-5-morpholino-3,4-dihydroisoquinoline-2­(1*H*)-carboxylate (**20d**). The crude material was purified with column chromatography, starting with DCM and increasing the polarity with DCM:MeOH:NH_3_ (7N in MeOH) (90:10:2), to give a 79% yield of a yellow foam.

^1^H NMR (500 MHz, CDCl_3_): δ 8.38 (s, 1H), 7.40–7.30 (m, 1H), 7.22–7.13 (m, 1H), 7.10–7.01 (m, 1H), 6.93 (dd, *J =* 7.9, 1.1 Hz, 1H), 6.87 (s, 1H), 4.68 (d, *J =* 16.6 Hz, 1H), 4.48 (s, 1H), 4.32 (d, *J =* 16.5 Hz, 1H), 3.91 (ddd, *J =* 11.0, 6.5, 2.8 Hz, 2H), 3.85–3.69 (m, 2H), 3.47–3.29 (m, 1H), 3.12–2.90 (m, 2H), 2.89–2.61 (m, 6H), 2.61–2.25 (m, 2H), 1.95 (dd, *J =* 15.9, 9.7 Hz, 2H), 1.65 (d, *J =* 6.0 Hz, 2H), 1.51 (s, 9H).

Compounds **22** and **23** were synthesized following these steps:

Step 1.

##### *tert*-Butyl*(R)*-3-(((4-(bis­(*tert*-butoxycarbonyl)­amino)­butyl)­(*(S)*-5,6,7,8-tetrahydroquinolin-8-yl)­amino)­methyl)-5-bromo-3,4-dihydroisoquinoline-2­(1*H*)-carboxylate and *tert*-Butyl*(S)*-3-(((4-(bis­(*tert*-butoxycarbonyl)­amino)­butyl)­(*(S)*-5,6,7,8-tetrahydroquinolin-8-yl)­amino)­methyl)-5-bromo-3,4-dihydroisoquinoline-2­(1*H*)-carboxylate

Procedure C was used, starting with *tert*-butyl 5-bromo-3-(((*(S)*-5,6,7,8-tetrahydroquinolin-8-yl)­amino)­methyl)-3,4-dihydroisoquinoline-2­(1*H*)-carboxylate (**21a**) and *tert*-butyl (*tert*-butoxycarbonyl)­(4-oxobutyl)­carbamate (**14**). The diastereomers were separated by column chromatography, starting with DCM and increasing the polarity with MeOH, slowly to 10% MeOH in DCM.

##### *tert*-Butyl-*(R)*-3-(((4-(bis­(*tert*-butoxycarbonyl)­amino)­butyl)­(*(S)*-5,6,7,8-tetrahydroquinolin-8-yl)­amino)­methyl)-5-bromo-3,4-dihydroisoquinoline-2­(1*H*)-carboxylate (81 mg, White Foam)

^1^H NMR (500 MHz, CDCl_3_): δ 8.35–8.27 (m, 1H), 7.42–7.34 (m, 1H), 7.30–7.21 (m, 1H), 7.03–6.88 (m, 3H), 4.81–4.60 (m, 1H), 4.51–4.39 (m, 1H), 4.08 (t, *J =* 21.7 Hz, 1H), 3.92–3.71 (m, 1H), 3.51 (t, *J =* 7.4 Hz, 2H), 3.40–3.16 (m, 1H), 2.99–2.53 (m, 4H), 2.39 (s, 1H), 2.21–1.84 (m, 3H), 1.80–1.71 (m, 1H), 1.70–1.58 (m, 6H), 1.52 (s, 9H), 1.49 (s, 18H).

##### *tert*-Butyl-*(S)*-3-(((4-(bis­(*tert*-butoxycarbonyl)­amino)­butyl)­(*(S)*-5,6,7,8-tetrahydroquinolin-8-yl)­amino)­methyl)-5-bromo-3,4-dihydroisoquinoline-2­(1*H*)-carboxylate (52 mg of an Off-White Foam)

^1^H NMR (400 MHz, CDCl_3_): δ 8.29 (s, 1H), 7.37 (s, 1H), 7.24 (d, *J =* 1.5 Hz, 3H), 6.96 (d, *J =* 8.5 Hz, 2H), 5.28 (d, *J =* 1.6 Hz, 1H), 4.69 (d, *J =* 18.9 Hz, 1H), 4.42–3.81 (m, 1H), 3.67–3.57 (m, 1H), 3.51 (s, 1H), 3.28 (d, *J =* 34.1 Hz, 1H), 3.02–2.40 (m, 2H), 2.36 (td, *J =* 7.4, 1.6 Hz, 1H), 1.92–1.77 (m, 1H), 1.48 (m, 6H), 1.47 (s, 9H), 1.45 (s, 18H), 1.45–1.38 (m, 5H).

Step 2.

##### N^1^-((*(R)*-5-Bromo-1,2,3,4-tetrahydroisoquinolin-3-yl)­methyl)-N^1^-(*(S)*-5,6,7,8-tetrahydroquinolin-8-yl)­butane-1,4-diamine (**22**)

Procedure A was used, starting with *tert*-butyl *(R)*-3-(((4-(bis­(*tert*-butoxycarbonyl)­amino)­butyl)­(*(S)*-5,6,7,8-tetrahydroquinolin-8-yl)­amino)­methyl)-5-bromo-3,4-dihydroisoquinoline-2­(1*H*)-carboxylate to give an 83% yield of a white foam.

^1^H NMR (600 MHz, CDCl_3_): δ 8.53 (d, *J =* 7.2 Hz, 1H), 7.33 (m, 2H), 7.08 (q, *J =* 4.2, 7.2 Hz, 1H), 6.92 (m, 3H), 4.05 (q, *J =* 6.6, 9 Hz, 2H), 3.54 (d, 25 Hz, 1H), 3.04 (m, 2H), 2.87 (m, 1H), 2.77 (m, 1H), 2.65 (m, 6H), 2.25 (q, *J =* 11, 15.6 Hz, 1H), 2.15 (m, 1H), 2.02 (m, 1H), 1.76 (m, 3H), 1.54 (m, 2H); ^13^C NMR (150 MHz, CDCl_3_): δ 157.21, 146.79, 137.40, 137.34, 133.89, 133.36, 130.22, 127.17, 125.43, 125.38, 122.11, 62.52, 58.77, 54.12, 52.40, 47.47, 39.59, 34.06, 29.32, 26.91, 26.58, 23.59, 21.88; HRMS calculated for C_23_H_33_BrN_4_ 443.1811; found 443.1807 [M + H].

##### N^1^-((*(S)*-5-Bromo-1,2,3,4-tetrahydroisoquinolin-3-yl)­methyl)-N^1^-(*(S)*-5,6,7,8-tetrahydroquinolin-8-yl)­butane-1,4-diamine (**23**)

Procedure A was used, starting with *tert*-butyl *(S)*-3-(((4-(bis­(*tert*-butoxycarbonyl)­amino)­butyl)­(*(S)*-5,6,7,8-tetrahydroquinolin-8-yl)­amino)­methyl)-5-bromo-3,4-dihydroisoquinoline-2­(1*H*)-carboxylate to give a 52% yield of a white foam.

^1^H NMR (600 MHz, CDCl_3_): δ 8.53 (d, *J =* 4.8 Hz, 1H), 7.38 (d, *J =* 7.5 Hz, 1H), 7.49 (d, *J =* 6.8 Hz, 1H), 7.10 (dd, *J =* 2.8, 7.6 Hz, 1H), 6.98 (m, 2H), 4.60 (bs, 6H), 4.08 (d, *J =* 16 Hz, 1H), 4.06 (m, 1H), 4.00 (d, *J =* 16 Hz), 3.54 (d, 25 Hz, 1H), 3.04 (m, 2H), 2.87 (m, 1H), 2.77 (m, 1H), 2.65 (m, 6H), 2.25 (q, *J =* 11, 15.6 Hz, 1H), 2.15 (m, 1H), 2.02 (m, 1H), 1.76 (m, 3H), 1.54 (m, 2H); ^13^C NMR (150 MHz, CDCl_3_): δ 157.21, 146.79, 137.40, 137.34, 133.89, 133.36, 130.22, 127.17, 125.43, 125.38, 122.11, 62.52, 58.77, 54.12, 52.40, 47.47, 39.59, 34.06, 29.32, 26.91, 26.58, 23.59, 21.88; HRMS calculated for C_23_H_33_BrN_4_ 443.1811; found 443.1805 [M + H].

Compounds **24** and **25** were synthesized by the following steps.

Step 1.

##### *tert*-Butyl*(R)*-3-(((4-(bis­(*tert*-butoxycarbonyl)­amino)­butyl)­(*(S)*-5,6,7,8-tetrahydroquinolin-8-yl)­amino)­methyl)-6-chloro-3,4-dihydroisoquinoline-2­(1*H*)-carboxylate and *tert*-butyl*(S)*-3-(((4-(bis­(*tert*-butoxycarbonyl)­amino)­butyl)­(*(S)*-5,6,7,8-tetrahydroquinolin-8-yl)­amino)­methyl)-6-chloro-3,4-dihydroisoquinoline-2­(1*H*)-carboxylate

Procedure C was used, starting with *tert*-butyl *(R)*-7-chloro-3-(((*(S)*-5,6,7,8-tetrahydroquinolin-8-yl)­amino)­methyl)-3,4-dihydroisoquinoline-2­(1*H*)-carboxylate (**21b**) and *tert*-butyl-(*tert*-butoxycarbonyl)­(4-oxobutyl)­carbamate (**14**). The diastereomers were separated by column chromatography, starting with DCM and increasing the polarity with DCM/MeOH/NH_3_ (7N in MeOH) solution (90:10:2).

##### *tert*-Butyl *(R)*-3-(((4-(bis­(*tert*-butoxycarbonyl)­amino)­butyl)­(*(S)*-5,6,7,8-tetrahydroquinolin-8-yl)­amino)­methyl)-6-chloro-3,4-dihydroisoquinoline-2­(1*H*)-carboxylate (76% Yield of a Yellow Foam)

^1^H NMR (400 MHz, CDCl_3_): δ 8.24 (d, *J =* 13.3 Hz, 1H), 7.17 (d, *J =* 7.7 Hz, 1H), 6.96 (dd, *J =* 8.0, 2.2 Hz, 1H), 6.94–6.79 (m, 3H), 5.45–4.93 (m, 1H), 4.53 (d, *J =* 16.8 Hz, 1H), 4.08–3.85 (m, 1H), 3.77 (d, *J =* 32.4 Hz, 1H), 3.65–3.37 (m, 7H), 3.37–3.04 (m, 1H), 3.05–2.69 (m, 1H), 2.69–2.39 (m, 2H), 2.39–2.20 (m, 1H), 2.10–1.62 (m, 3H), 1.60–1.46 (m, 3H), 1.41 (s, 18H), 1.38 (s, 9H).

##### *tert*-Butyl *(S)*-3-(((4-(bis­(*tert*-butoxycarbonyl)­amino)­butyl)­(*(S)*-5,6,7,8-tetrahydroquinolin-8-yl)­amino)­methyl)-6-chloro-3,4-dihydroisoquinoline-2­(1*H*)-carboxylate (35% Yield, Yellow Foam)

^1^H NMR (400 MHz, CDCl_3_): δ 8.30 (dd, *J =* 4.7, 1.7 Hz, 1H), 7.25–7.16 (m, 2H), 7.08–6.95 (m, 3H), 4.56 (d, *J =* 16.5 Hz, 2H), 4.34–3.81 (m, 3H), 3.75–3.38 (m, 3H), 3.18–3.00 (m, 1H), 3.00–2.84 (m, 1H), 2.84–2.67 (m, 2H), 2.63–2.33 (m, 3H), 2.17–1.70 (m, 2H), 1.47 (d, *J =* 3.7 Hz, 18H), 1.43 (s, 9H), 1.39–1.26 (m, 5H).

Step 2.

##### N^1^-((*(R)*-7-Chloro-1,2,3,4-tetrahydroisoquinolin-3-yl)­methyl)-N^1^-(*(S)*-5,6,7,8-tetrahydroquinolin-8-yl)­butane-1,4-diamine (**24**)

Procedure A was use,d starting with *tert*-butyl *(R)*-3-(((4-(bis­(*tert*-butoxycarbonyl)­amino)­butyl)­(*(S)*-5,6,7,8-tetrahydroquinolin-8-yl)­amino)­methyl)-6-chloro-3,4-dihydroisoquinoline-2­(1*H*)-carboxylate to afford the title compound in 45% yield as a light-yellow foam.

^1^H NMR (400 MHz, CDCl_3_): δ 8.33 (d, *J =* 4.7 Hz, 1H), 7.19 (d, *J =* 7.7 Hz, 1H), 7.09–6.66 (m, 4H), 3.98–3.82 (m, 2H), 3.65 (d, *J =* 15.5 Hz, 1H), 3.38 (s, 2H), 2.84 (dt, *J =* 12.9, 4.8 Hz, 2H), 2.65–2.38 (m, 7H), 2.29–2.16 (m, 2H), 1.94 (t, *J =* 6.1 Hz, 1H), 1.86–1.67 (m, 3H), 1.57 (dq, *J =* 11.1, 5.7, 5.2 Hz, 1H), 1.48–1.32 (m, 4H); ^13^C NMR (101 MHz, CDCl_3_): δ 158.2, 146.5, 137.3, 136.4, 133.7, 132.9, 130.6, 130.1, 125.9, 125.8, 121.3, 61.2, 57.7, 54.0, 51.9, 48.1, 41.4, 33.0, 30.3, 29.2, 27.9, 26.9, 21.8; HRMS calculated for C_23_H_32_ClN_4_ 399.23155; found 399.23061 [M + H].

##### N^1^-((*(R)*-7-Chloro-1,2,3,4-tetrahydroisoquinolin-3-yl)­methyl)-N^1^-(*(S)*-5,6,7,8-tetrahydroquinolin-8-yl)­butane-1,4-diamine (**25**)

Procedure A was used, starting with *tert*-butyl-*(S)*-3-(((4-(bis­(*tert*-butoxycarbonyl)­amino)­butyl)­(*(S)*-5,6,7,8-tetrahydroquinolin-8-yl)­amino)­methyl)-6-chloro-3,4-dihydroisoquinoline-2­(1*H*)-carboxylate to afford the title compound with a 70.5% yield of an off-white foam.

^1^H NMR (400 MHz, CDCl_3_): δ 8.38 (dd, *J =* 4.7, 1.7 Hz, 1H), 7.19 (dd, *J =* 7.7, 1.7 Hz, 1H), 6.95–6.82 (m, 2H), 6.83–6.74 (m, 2H), 3.96–3.88 (m, 1H), 3.84 (s, 7H), 2.77–2.69 (m, 1H), 2.69–2.53 (m, 4H), 2.53–2.41 (m, 1H), 2.41–2.26 (m, 2H), 2.19 (dd, *J =* 16.2, 10.8 Hz, 1H), 2.06–1.93 (m, 1H), 1.93–1.78 (m, 1H), 1.74–1.58 (m, 1H), 1.58–1.46 (m, 1H), 1.44–1.27 (m, 4H); ^13^C NMR (101 MHz, CDCl_3_): δ 156.61, 146.84, 137.41, 136.52, 134.07, 132.92, 130.56, 129.63, 125.81, 125.61, 121.57, 61.24, 57.09, 51.88, 51.75, 48.38, 40.94, 31.59, 29.91, 29.04, 26.13, 23.47, 21.24; HRMS calculated for C_23_H_32_ClN_4_ 399.23155; found 399.23061 [M + H].

Compounds **26** and **27** were synthesized by the following steps.

Step 1.

##### *tert*-Butyl*(R)*-3-(((4-(bis­(*tert*-butoxycarbonyl)­amino)­butyl)­(*(S)*-5,6,7,8-tetrahydroquinolin-8-yl)­amino)­methyl)-5-(4-(*tert*-butoxycarbonyl)­piperazin-1-yl)-3,4-dihydroisoquinoline-2­(1*H*)-carboxylate and *tert*-butyl*(S)*-3-(((4-(bis­(*tert*-butoxycarbonyl)­amino)­butyl)­(*(S)*-5,6,7,8-tetrahydroquinolin-8-yl)­amino)­methyl)-5-(4-(*tert*-butoxycarbonyl)­piperazin-1-yl)-3,4-dihydroisoquinoline-2­(1*H*)-carboxylate

Procedure C was used, starting with *tert*-butyl 5-(4-(*tert*-butoxycarbonyl)­piperazin-1-yl)-3-(((*(S)*-5,6,7,8-tetrahydroquinolin-8-yl)­amino)­methyl)-3,4-dihydroisoquinoline-2­(1*H*)-carboxylate (**21c**) and (*tert*-butoxycarbonyl)­(4-oxobutyl)­carbamate (**14**). The diastereomers were separated by column chromatography, starting with DCM and increasing the polarity with MeOH slowly to 10% MeOH in DCM.

(*S,R*)-Diastereomer as a yellow gel (96% yield). LC-MS (ESI-API, 254 nm), 75–95% MeOH in H_2_O (0.1% HCO_2_H), 3 min, 1.00 mL/min, C18 (Agilent Zorbax XDB-18, 50 mm × 4.6 mm, 3.5 μm), *m*/*z* = 849.5 (M + H), t = 0.814 min; (*S,S*)-diastereomer as a yellow gel (40.3% yield). LC-MS (ESI-API, 254 nm), 75–95% MeOH in H_2_O (0.1% HCO_2_H), 3 min, 1.00 mL/min, C18 (Agilent Zorbax XDB-18, 50 mm × 4.6 mm, 3.5 μm), *m*/*z* = 849.2 (M + H), t = 0.823 min.

##### N^1^-((*(R)*-5-(Piperazin-1-yl)-1,2,3,4-tetrahydroisoquinolin-3-yl)­methyl)-N^1^-(*(S)*-5,6,7,8-tetrahydroquinolin-8-yl)­butane-1,4-diamine (**26**)

Procedure A was used, starting with *tert*-butyl *(R)*-3-(((4-(bis­(*tert*-butoxycarbonyl)­amino)­butyl)­(*(S)*-5,6,7,8-tetrahydroquinolin-8-yl)­amino)­methyl)-5-(4-(*tert*-butoxycarbonyl)­piperazin-1-yl)-3,4-dihydroisoquinoline-2­(1*H*)-carboxylate to give a 47% yield of a white foam.

^1^H NMR (400 MHz, CD_3_OD): δ 8.36 (dd, *J =* 4.6, 1.7 Hz, 1H), 7.50–7.35 (m, 1H), 7.13 (dd, *J =* 7.7, 4.7 Hz, 1H), 7.09–6.98 (m, 1H), 6.86 (t, *J =* 6.8 Hz, 1H), 6.78–6.67 (m, 1H), 4.05–3.94 (m, 2H), 3.71 (d, *J =* 15.5 Hz, 1H), 3.27 (s, 3H), 3.04–2.47 (m, 19H), 2.20–2.04 (m, 2H), 2.00–1.84 (m, 2H), 1.67 (t, *J =* 11.7 Hz, 1H), 1.58–1.40 (m, 4H); ^13^C NMR (101 MHz, CD_3_OD): δ 158.9, 153.0, 147.6, 138.9, 136.4, 136.2, 130.4, 127.7, 123.4, 123.0, 118.5, 64.3, 59.3, 54.6, 54.1, 53.6, 52.9, 50.9, 49.9, 46.9, 41.2, 30.3, 30.3, 28.5, 27.7, 26.3, 22.9; HRMS calculated for C_27_H_41_N_6_ 449.33927; found 449.33961 [M + H].

##### N^1^-((*(S)*-5-(Piperazin-1-yl)-1,2,3,4-tetrahydroisoquinolin-3-yl)­methyl)-N^1^-(*(S)*-5,6,7,8-tetrahydroquinolin-8-yl)­butane-1,4-diamine (**27**)

Procedure A was used, starting with *tert*-butyl *(S)*-3-(((4-(bis­(*tert*-butoxycarbonyl)­amino)­butyl)­(*(S)*-5,6,7,8-tetrahydroquinolin-8-yl)­amino)­methyl)-5-(4-(*tert*-butoxycarbonyl)­piperazin-1-yl)-3,4-dihydroisoquinoline-2­(1*H*)-carboxylate to give a 71% yield of a white foam.

^1^H NMR (400 MHz, CDCl_3_): δ 8.56 (d, *J =* 4.7 Hz, 1H), 7.35 (d, *J =* 3.6 Hz, 1H), 7.21–7.01 (m, 2H), 6.85 (dd, *J =* 29.9, 7.7 Hz, 2H), 4.11 (d, *J =* 19.8 Hz, 3H), 3.09–2.57 (m, 20H), 2.26–2.11 (m, 2H), 2.04 (tt, *J =* 9.1, 4.5 Hz, 1H), 1.93–1.81 (m, 1H), 1.81–1.35 (m, 6H); ^13^C NMR (101 MHz, CDCl_3_): δ 157.8, 151.7, 147.2, 136.7, 136.6, 134.2, 130.0, 126.0, 121.7, 121.7, 116.6, 61.4, 58.3, 53.1, 52.6, 52.2, 48.8, 46.6, 41.8, 31.1, 30.1, 29.4, 26.7, 24.2, 21.6; HRMS calculated for C_27_H_41_N_6_ 449.33927; found 449.33852 [M + H].

Compound **28** was synthesized according to the following steps.

Step 1.

##### *tert*-Butyl*(R)*-3-(((4-(bis­(*tert*-butoxycarbonyl)­amino)­butyl)­(*(S)*-5,6,7,8-tetrahydroquinolin-8-yl)­amino)­methyl)-5-morpholino-3,4-dihydroisoquinoline-2­(1*H*)-carboxylate

Procedure C was used, starting with *tert*-butyl *(R)*-5-morpholino-3-(((*(S)*-5,6,7,8-tetrahydroquinolin-8-yl)­amino)­methyl)-3,4-dihydroisoquinoline-2­(1*H*)-carboxylate (**21d**) and (*tert*-butoxycarbonyl)­(4-oxobutyl)­carbamate (**14**) to get a 93% yield of a white foam.

^1^H NMR (400 MHz, CDCl_3_): δ 8.28 (br s, 1H), 7.26 (d, 1H, J = 6 Hz), 7.10 (t, 1H, J = 8 Hz), 6.95 (dd, 1H, J = 5, 8 Hz), 6.84 (d, 1H, J = 8 Hz), 6.76 (d, 1H, J = 7 Hz), 4.65 (m, 2H), 4.36 (br s, 1H), 4.10 (d, 1H, J = 17 Hz), 3.90 (m, 2H), 3.82 (t, 2H, J = 6 Hz), 3.60 (m, 1H), 3.44 (m, 2H), 3.05 (m, 2H), 2.73 (m, 6H), 2.62 (m, 2H), 2.46 (heptet, 1H, J = 4 Hz), 2.25 (s, 1H), 2.12 (s, 1H), 1.92 (s, 1H), 1.51–1.76 (m, 5H), 1.48 (s, 9H), 1.45 (s, 18H); MS: 750.5 *m*/*z* (M+H)^+^.

Step 2.

##### N^1^-((*(R)*-5-Morpholino-1,2,3,4-tetrahydroisoquinolin-3-yl)­methyl)-N^1^-(*(S)*-5,6,7,8-tetrahydroquinolin-8-yl)­butane-1,4-diamine (**28**)

Procedure A was used, starting with *tert*-butyl *(R)*-3-(((4-(Bis­(*tert*-butoxycarbonyl)­amino)­butyl)­(*(S)*-5,6,7,8-tetrahydroquinolin-8-yl)­amino)­methyl)-5-morpholino-3,4-dihydroisoquinoline-2­(1*H*)-carboxylate to afford 53% of a white foam.

^1^H NMR (400 MHz, CDCl_3_): δ 8.45 (dd, 1H, J = 1, 4 Hz), 7.34 (d, 1H, J = 8 Hz), 7.10 (t, 1H, J = 8 Hz), 7.05 (dd, 1H, J = 5, 8 Hz), 6.86 (d, 1H, J = 8 Hz), 6.78 (d, 1H, J = 8 Hz), 4.13 (d, 1H, J = 16 Hz), 4.06 (dd, 1H, J = 6, 10 Hz), 3.88 (d, 1H, J = 16 Hz), 3.78 (qq, 4H, J = 3, 11 Hz), 3.06 (dd, 1H, J = 2, 13 Hz), 2.97 (m, 2H), 2.86 (m, 1H), 2.65–2.79 (m, 7H), 2.46–2.61 (m, 4H), 2.16 (dd, 1H, J = 10, 16 Hz), 2.10 (m, 1H), 1.98 (m, 1H), 1.87 (m, 1H), 1.71 (m, 1H), 1.51 (m, 2H), 1.39 (m, 1H); ^13^C NMR (101 MHz, CDCl_3_): δ 158.41, 151.04, 146.67, 136.76, 136.13, 133.99, 129.74, 126.23, 122.02, 121.58, 116.83, 74.76, 67.43, 62.04, 58.43, 54.17, 52.38, 52.19, 48.34, 41.60, 29.90, 29.40, 27.93, 27.24, 21.96.

LC-MS: 99% @ 0.53 min for 450.2 *m*/*z* (M+H)^+^; HRMS: calculated for C_27_H_40_ON_5_ 450.32274, found 450.32243 [M + H].

##### *(R)*-9-Morpholino-1,5,10,10a-tetrahydro-3*H*-oxazolo­[3,4-*b*]­isoquinolin-3-one (**30**)

Procedure B was used, starting with (10a*R*)-9-morpholino-1,5,10,10a-tetrahydrooxazolo­[3,4-*b*]­isoquinolin-3-one (**29**) and it was purified with column chromatography using an EA:hexanes solvent system to afford 69% of a yellow foam.

^1^H NMR (600 MHz, CDCl_3_): δ 7.28–7.25 (m, 1H), 7.00 (dd, *J =* 8.0, 1.1 Hz, 1H), 6.95 (dt, *J =* 7.7, 1.1 Hz, 1H), 4.86 (d, *J =* 16.8 Hz, 1H), 4.62 (dd, *J =* 8.6, 7.8 Hz, 1H), 4.42 (dq, *J =* 16.7, 0.8 Hz, 1H), 4.24 (dd, *J =* 8.6, 4.8 Hz, 1H), 3.93–3.80 (m, 5H), 3.41 (dd, *J =* 15.3, 3.7 Hz, 1H), 3.04 (s, 2H), 2.85–2.67 (m, 2H), 2.60–2.38 (m, 1H).

***tert*** **-Butyl*(R)*-3-formyl-5-morpholino-3,4-dihydroisoquinoline-2­(1*H*)-carboxylate (32)** was synthesized according to the following steps.

Step 1.

##### *(R)*-(5-Morpholino-1,2,3,4-tetrahydroisoquinolin-3-yl)­methanol

(10a*R*)-9-morpholino-1,5,10,10a-tetrahydrooxazolo­[3,4-*b*]­isoquinolin-3-one (**29**) (1.20 g, 4.37 mmol) was suspended in MeOH (25 mL), and 6 M NaOH (7.29 mL, 43.74 mmol) was added to the reaction mixture. The reaction was heated to 100 °C (oil bath temp) for 2 h. TLC and LCMS indicated that all starting material was consumed. The solvent was evaporated, and some water was added . The mixture was stirred at room temperature overnight. Then, the extra water was decanted from the flask, and the process was continued to the next step.

Step 2.

##### *tert*-Butyl*(R)*-3-(hydroxymethyl)-5-morpholino-3,4-dihydroisoquinoline-2­(1*H*)-carboxylate

To the solution of (3*R*)-5-morpholino-1,2,3,4-tetrahydroisoquinolin-3-yl]­methanol (1.08 g, 4.35 mmol) in THF (10 mL), sodium carbonate (1.38 g, 13.05 mmol) and di-*tert*-butyl dicarbonate (1.51 mL, 6.52 mmol) were added and stirred at room temperature overnight. The reaction mixture was diluted with EA and extracted with water 2 times. The organic phase was dried over anhydrous MgSO_4_, filtered off, and evaporated. The residue was purified with column chromatography with EA:hexanes (1:1) to afford 83% of the desired compound in 2 steps as a yellowish foam.

^1^H NMR (400 MHz, CDCl_3_): δ 7.21 (t, *J =* 7.8 Hz, 1H), 7.00–6.94 (m, 1H), 6.91 (d, *J =* 7.5 Hz, 1H), 4.66 (d, *J =* 16.0 Hz, 1H), 4.32 (d, *J =* 16.2 Hz, 1H), 3.87 (qdd, *J =* 11.0, 7.7, 3.0 Hz, 4H), 3.49 (s, 2H), 2.99 (s, 2H), 2.91–2.75 (m, 2H), 1.64 (dq, *J =* 6.7, 3.5, 2.9 Hz, 4H), 1.53 (s, 9H).

Step 3.

##### *tert*-Butyl*(R)*-3-formyl-5-morpholino-3,4-dihydroisoquinoline-2­(1*H*)-carboxylate (**32**)

*tert*-Butyl (3*R*)-3-(hydroxymethyl)-5-morpholino-3,4-dihydro-1*H*-isoquinoline-2-carboxylate (1.26 g, 3.62 mmol) was dissolved in DCM (10 mL) and cooled down to 0 °C with an ice bath, and TEA (2.52 mL, 18.08 mmol) was added to the solution. A solution of pyridine sulfur trioxide (2.30 g, 14.46 mmol) in DMSO (10 mL) was added dropwise to the reaction mixture. The reaction was stirred at 0 °C for 3 h. Then, a sodium bicarbonate solution was added to the reaction mixture, and diluted with EA, and stirred for 30 min. The aqueous phase was extracted with EA. The combined organic layer was dried over anhydrous MgSO_4_, filtered off, and the filtrate was evaporated. It was used as is for the next step.

##### *tert*-Butyl*(R)*-5-(4,4-Ddfluoropiperidin-1-yl)-3-formyl-3,4-dihydroisoquinoline-2­(1*H*)-carboxylate (**33**)

Synthesized by the following steps.

Step 1.

##### *(R)*-9-(4,4-Difluoropiperidin-1-yl)-1,5,10,10a-tetrahydro-3*H*-oxazolo­[3,4-*b*]­isoquinolin-3-one (**31**)

A 20 mL μW tube equipped with a stir bar was charged with 3.00 g of the bromide (11.2 mmol, 1 equiv), 26.1 mg of RuPhos (0.0559 mmol, 0.005 equiv), 1.29 g of NaOtBu (13.4 mmol, 1.2 equiv), and 46.8 mg of RuPhos Pd G3 (0.0559 mmol, 0.005 equiv), and the system was set under an Ar atmosphere. Then, 11.1 mL of THF (degassed by bubbling through Ar for 1 h) and 1.51 mL of the amine (13.4 mmol, 1.2 equiv) were added. After stirring at 85 °C for 4 h, CH_2_Cl_2_ and water were added, and the product was extracted with CH_2_Cl_2_ (3x) and dried over Na_2_SO_4_. After the organics were concentrated, the crude product (3.65 g) was crystallized from toluene, affording 1.75 g of the product as yellowish crystals. The filtrate was concentrated, dissolved in CH_2_Cl_2_ and precipitated out by the addition of ether, affording a second fraction of the product (0.45 g). The rest was purified on a column (40 g) using 0 to 20% EA in CH_2_Cl_2_ as the eluent affording 0.342 g of the product.

^1^H NMR (400 MHz, CDCl_3_): δ 7.23 (t, *J =* 7.9 Hz, 1H), 6.99 (d, *J =* 7.9 Hz, 1H), 6.93 (d, *J =* 7.3 Hz, 1H), 4.84 (A of AB, *J*_*AB*_ = 16.9 Hz, 1H), 4.61 (A of ABX, *J*_*AB*_ = 8.7 Hz, *J*_*AX*_ = 7.8 Hz, 1H), 4.39 (B of AB, *J*_*AB*_ = 16.9 Hz, 1H), 4.22 (B of ABX, *J*_*AB*_ = 8.7 Hz, *J*_*BX*_ = 4.7 Hz, 1H), 3.87 (ddt, *J =* 11.6, 8.1, 4.2 Hz, 1H), 3.34 (A of ABX, *J*_*AB*_ = 15.4 Hz, *J*_*AX*_ = 3.7 Hz, 1H), 3.12–3.03 (m, 2H), 2.88 (br s, 2H), 2.49 (B of ABX, *J*_*AB*_ = 15.4 Hz, *J*_*BX*_ = 10.9 Hz, 1H), 2.23–2.01 (m, 4H); LC-MS (ESI-API, 254 nm) 75–95% MeOH in H_2_O (0.1% HCO_2_H), 3 min, 1.00 mL/min, C18 (Agilent Zorbax XDB-18, 50 mm × 4.6 mm, 3.5 μm), *m*/*z* = 639.2 (2 M + Na), 331.0 (M + Na), 309.2 (M + H), t = 1.011 min.

Step 2.

##### *(R)*-(5-(4,4-Difluoropiperidin-1-yl)-1,2,3,4-tetrahydroisoquinolin-3-Yyl)­methanol

A 100 mL round-bottom flask equipped with a reflux condenser and a magnetic stir bar was charged with 2.54 g of the oxazolidinone (8.24 mmol, 1 equiv) and 20 mL of MeOH. Then, 13.7 mL of 6N NaOH solution (82.4 mmol, 10 equiv) was added, and the reaction mixture was refluxed (oil bath at 100 °C, keeps slight reflux). After heating for 3 h, the reaction mixture was allowed to cool to rt, and the product was crystallized out. 40 mL of water was added, and the suspension was stirred for 30 min, then cooled in an ice bath. The solids were filtered, washed with water (soluble in alcohols), and dried, affording 2.13 g of the product as a white solid.

^1^H NMR (400 MHz, CD_3_OD): δ 7.11 (t, *J =* 7.7 Hz, 1H), 6.95 (d, *J =* 7.9 Hz, 1H), 6.82 (d, *J =* 7.6 Hz, 1H), 4.05 (A of AB, *J*_*AB*_ = 15.9 Hz, 1H), 4.00 (B of AB, *J*_*AB*_ = 16.1 Hz, 1H), 3.72 (A of ABX, *J*_*AB*_ = 10.9 Hz, *J*_*AX*_ = 4.7 Hz, 1H), 3.59 (B of ABX, *J*_*AB*_ = 10.9 Hz, *J*_*BX*_ = 7.1 Hz, 1H), 3.11–3.00 (m, 2H), 2.97 (A of ABX, *J*_*AB*_ = 16.6 Hz, *J*_*AX*_ = 3.8 Hz, 1H), 2.92–2.76 (m, 3H), 2.36 (B of ABX, *J*_*AB*_ = 16.6 Hz, *J*_*BX*_ = 10.7 Hz, 1H), 2.19–1.98 (m, 4H), OH and NH to OD and ND exchange observed; ^13^C NMR (100 MHz, CD_3_OD): δ 152.36, 137.05, 130.51, 127.47, 123.13, 123.10 (t, *J =* 240.8 Hz), 118.62, 66.49, 56.51, 50.33 (t, *J =* 5.7 Hz), 48.89, 35.84 (t, *J =* 22.8 Hz), 28.24; LC-MS (ESI-API, 254 nm) 75–95% MeOH in H_2_O (0.1% HCO_2_H), 3 min, 1.00 mL/min, C18 (Agilent Zorbax XDB-18, 50 mm × 4.6 mm, 3.5 μm), *m*/*z* = 305.2 (M + Na), 283.2 (M + H), t = 0.550 min.

Step 3.

##### *tert*-Butyl*(R)*-5-(4,4-difluoropiperidin-1-yl)-3-(hydroxymethyl)-3,4-dihydroisoquinoline-2­(1*H*)-carboxylate

A 100 L round-bottom flask equipped with a stir bar and rubber septum was charged with 2.10 g of the amine (7.44 mmol, 1 equiv) and 25 mL of THF. Then, 1.58 g of Na_2_CO_3_ (14.9 mmol, 2 equiv) dissolved in 12 mL of water, followed by 1.79 g of di-*tert*-butyl dicarbonate (8.18 mmol, 1.1 equiv), were added, and the reaction mixture was stirred at rt for 4 h. The reaction mixture was quenched by the addition of water, extracted with CH_2_Cl_2_ (3x), and dried over Na_2_SO_4_. The organics were concentrated, and the crude product was purified on a silica gel column (40 g) using 0 to 40% EA in hexanes as the eluent, affording 2.77 g of the product as a white foam.

^1^H NMR (400 MHz, CDCl_3_): δ 7.16 (t, *J =* 7.8 Hz, 1H), 6.95 (dd, *J =* 8.0, 1.1 Hz, 1H), 6.87 (d, *J =* 7.6 Hz, 1H), 4.76–4.59 (m, 1H), 4.52–4.35 (m, 1H), 4.29 (d, *J =* 16.4 Hz, 1H), 3.54–3.32 (m, 2H), 3.15–2.86 (m, 5H), 2.81 (dd, *J =* 15.9, 6.0 Hz, 1H), 2.34 (br s, 1H), 2.23–2.02 (m, 4H), 1.49 (s, 9H); ^13^C NMR (100 MHz, CDCl_3_): δ 156.37, 150.67, 134.70, 127.94, 126.69, 121.67, 121.67 (t, *J =* 241.8 Hz), 117.64, 80.32, 64.10, 62.93 (other conformer), 51.93, 49.19 (t, *J =* 5.4 Hz), 44.32, 43.69 (other conformer), 34.63 (t, *J =* 22.8 Hz), 28.39, 25.28.

Step 4.

##### *tert*-Butyl*(R)*-5-(4,4-difluoropiperidin-1-yl)-3-formyl-3,4-dihydroisoquinoline-2­(1*H*)-carboxylate (**33**)

A 100 mL round-bottom flask equipped with a magnetic stir bar and septum was charged with 2.68 g of *tert*-butyl *(R)*-5-(4,4-difluoropiperidin-1-yl)-3-(hydroxymethyl)-3,4-dihydroisoquinoline-2­(1*H*)-carboxylate (7.01 mmol, 1 equiv), 5.18 mL of NEt_3_ (37.1 mmol, 5.3 equiv), and 27 mL of CH_2_Cl_2_. After the reaction mixture was cooled to 0 °C, 4.46 g of SO_3_*Py (28.0 mmol, 4 equiv) dissolved in 9 mL of DMSO (dissolved in another flask under Ar) was added dropwise, and the reaction mixture was stirred at 0 °C for 2 h. Then the reaction mixture was quenched by the addition of sat. NaHCO_3_ solution, extracted with CH_2_Cl_2_ (3x), washed with water, and dried over Na_2_SO_4_. After the organics were concentrated, the aldehyde was used in the next step without further purification.

##### *tert*-Butyl*(R)*-5-morpholino-3-(((*(S)*-5,6,7,8-tetrahydroquinolin-8-yl)­amino)­methyl)-3,4-dihydroisoquinoline-2­(1*H*)-carboxylate (**34**)

Procedure C was used, starting with **32** and **12,** to afford a 73% yield of an off-white foam.

^1^H NMR (400 MHz, CDCl_3_): δ 8.38 (s, 1H), 7.36 (d, *J =* 7.5 Hz, 1H), 7.21–7.14 (m, 1H), 7.06 (s, 1H), 6.95–6.90 (m, 1H), 6.87 (s, 1H), 4.74–4.59 (m, 1H), 4.49 (s, 1H), 4.38–4.26 (m, 1H), 3.91 (ddd, *J =* 11.0, 6.4, 2.8 Hz, 2H), 3.81 (ddd, *J =* 11.1, 6.3, 2.7 Hz, 2H), 3.73 (s, 1H), 3.43–3.32 (m, 1H), 3.10–2.98 (m, 2H), 2.87–2.62 (m, 7H), 2.39 (d, *J =* 14.6 Hz, 1H), 2.01–1.88 (m, 2H), 1.70–1.60 (m, 2H), 1.51 (s, 9H); MS: 479.2 *m*/*z* (M+H)^+^.

##### *tert*-Butyl*(R)*-5-(4,4-difluoropiperidin-1-yl)-3-(((*(S)*-5,6,7,8-tetrahydroquinolin-8-yl)­amino)­methyl)-3,4-dihydroisoquinoline-2­(1*H*)-carboxylate (**35**)

Procedure C was used, starting with *(S)*-5,6,7,8-tetrahydroquinolin-8-amine *tert*-butyl *(R)*-5-(4,4-difluoropiperidin-1-yl)-3-formyl-3,4-dihydroisoquinoline-2­(1*H*)-carboxylate (**33**). The crude product was purified with column chromatography with 0–50% EA in hexanes as the eluent, affording an 84% yield of a yellowish thick oil.

^1^H NMR (400 MHz, CDCl_3_): δ 8.35 (s, 1H), 7.33 (s, 1H), 7.15 (t, *J =* 7.7 Hz, 1H), 7.05 (s, 1H), 6.92 (d, *J =* 8.0 Hz, 1H), 6.86 (s, 1H), 4.64 (A of AB, *J*_*AB*_ = 16.3 Hz, 1H), 4.51 (s, 1H), 4.33 (B of AB, *J*_*AB*_ = 16.4 Hz, 1H), 3.75 (s, 1H), 3.36 (d, *J =* 15.7 Hz, 1H), 3.17–3.04 (m, 2H), 2.91 (s, 2H), 2.83–2.65 (m, 4H), 2.58 (s, 1H), 2.43–1.85 (m, 7H), 1.70–1.59 (m, 2H), 1.51 (s, 9H); ^13^C NMR (100 MHz, CDCl_3_) δ: 157.40 (br s), 154.97, 150.79 (br s), 146.69, 136.69, 134.82 (br s), 132.12, 127.91 (br s), 126.51 (br s), 121.89 (t, *J =* 241.7 Hz), 121.74 (br s), 121.49 (br s), 117.22, 79.70 (br s), 56.42 (other rotamer), 56.22, 49.60, 49.14, 48.63 (other rotamer), 47.69, 47.51 (other rotamer), 44.08 (other rotamer), 43.74, 34.53 (t, *J =* 22.7 Hz), 28.72, 28.45, 28.34, 26.02 (br s), 19.20.

**4-(1,3-Dioxoisoindolin-2-yl)­butanal (36)** was prepared according to known procedures.^46^

##### *tert*-Butyl*(R)*-3-(((4-(1,3-dioxoisoindolin-2-yl)­butyl)­(*(S)*-5,6,7,8-tetrahydroquinolin-8-yl)­amino)­methyl)-5-morpholino-3,4-dihydroisoquinoline-2­(1*H*)-carboxylate (**37**)

Procedure C was used, starting with **34** and **36** with adding titanium­(IV) isopropoxide. It was purified with column chromatography, starting with DCM and increasing the polarity with DCM:MeOH:NH_3_ (in MeOH) (90:10:2), to afford a 96% yield of a colorless foam.

^1^H NMR (500 MHz, CDCl_3_): δ 8.36–8.24 (m, 1H), 7.83–7.81 (m, 2H), 7.70 (ddd, *J =* 5.4, 2.8, 1.1 Hz, 2H), 7.24 (dd, *J =* 7.8, 1.7 Hz, 1H), 7.08 (t, *J =* 7.8 Hz, 1H), 6.94 (dd, *J =* 7.7, 4.5 Hz, 1H), 6.84 (dd, *J =* 8.0, 1.1 Hz, 1H), 6.76 (t, *J =* 7.7 Hz, 1H), 4.65 (d, *J =* 17.1 Hz, 1H), 4.22–4.07 (m, 1H), 4.00–3.87 (m, 2H), 3.87–3.77 (m, 2H), 3.77–3.65 (m, 1H), 3.59 (p, *J =* 7.5 Hz, 3H), 3.49–3.24 (m, 1H), 2.83–2.55 (m, 6H), 2.50 (d, *J =* 7.8 Hz, 1H), 2.38–2.21 (m, 1H), 2.12 (s, 1H), 1.93 (s, 1H), 1.80–1.53 (m, 3H), 1.48 (d, *J =* 8.1 Hz, 9H), 1.39–1.10 (m, 4H).

##### *tert*-Butyl*(R)*-5-(4,4-difluoropiperidin-1-yl)-3-(((4-(1,3-dioxoisoindolin-2-yl)­butyl)­(*(S)*-5,6,7,8-tetrahydroquinolin-8-yl)­amino)­methyl)-3,4-dihydroisoquinoline-2­(1*H*)-carboxylate (**38**)

Procedure C was used, starting with **35** and **36**. The crude product was purified on a silica gel column using 0 to 30% EA in hexanes as the eluent, affording an 85% yield as a white foam.

^1^H NMR (400 MHz, CDCl_3_): δ 8.28 (d, *J =* 4.2 Hz, 1H), 7.81 (dd, *J =* 5.4, 3.0 Hz, 2H), 7.69 (dd, *J =* 5.5, 3.0 Hz, 2H), 7.24 (d, *J =* 7.5 Hz, 1H), 7.06 (t, *J =* 7.8 Hz, 1H), 6.95 (dd, *J =* 7.8, 4.5 Hz, 1H), 6.85 (dd, *J =* 8.0, 1.1 Hz, 1H), 6.79–6.72 (m, 1H), 4.69 (br s, 0.4H), 4.67 (A of AB, *J*_*AB*_ = 17.1 Hz, 1H), 4.40 (d, *J =* 6.7 Hz, 0.6H), 4.12 (t, *J =* 14.5 Hz, 1H), 3.82 (s, 0.4H), 3.65 (t, *J =* 7.5 Hz, 0.6H), 3.61–3.51 (m, 2H), 3.41 (d, *J =* 15.9 Hz, 0.6H), 3.27 (d, *J =* 16.1 Hz, 0.4H), 3.12 (s, 2H), 2.99–2.42 (m, 9H), 2.33–1.87 (m, 7H), 1.80–1.50 (m, 2H), 1.49 (s, 9H), 1.40–1.20 (m, 3H); LC-MS (ESI-API, 254 nm) 75–95% MeOH in H_2_O (0.1% HCO_2_H), 3 min, 1.00 mL/min, C18 (Agilent Zorbax XDB-18, 50 mm × 4.6 mm, 3.5 μm), *m*/*z* = 714.3 (M + H), t = 0.795 min.

**N^1^-((*(R)*-5-(4,4-Difluoropiperidin-1-yl)-1,2,3,4-tetrahydroisoquinolin-3-yl)­methyl)-N^1^-(*(S)*-5,6,7,8-tetrahydroquinolin-8-yl)­butane-1,4-diamine (39)** was synthesized with the following steps.

Step 1.

##### *tert*-Butyl*(R)*-3-(((4-aminobutyl)­(*(S)*-5,6,7,8-tetrahydroquinolin-8-yl)­amino)­methyl)-5-(4,4-difluoropiperidin-1-yl)-3,4-dihydroisoquinoline-2­(1*H*)-carboxylate

A 20 mL vial equipped with a magnetic stir bar was charged with 223 mg of *tert*-butyl *(R)*-5-(4,4-difluoropiperidin-1-yl)-3-(((4-(1,3-dioxoisoindolin-2-yl)­butyl)­(*(S)*-5,6,7,8-tetrahydroquinolin-8-yl)­amino)­methyl)-3,4-dihydroisoquinoline-2­(1*H*)-carboxylate (**38**) (0.312 mmol, 1 equiv) and 0.407 mL of a 24% hydrazine solution in water (3.12 mmol, 10 equiv) dissolved in 3.1 mL of MeOH. The reaction was not done after 12 h, so more 0.814 mL of the 24% hydrazine solution in water (6.24 mmol, 20 equiv) was added. After stirring at rt for 12 h, the reaction mixture was quenched by the addition of 2 M NaOH, extracted with CH_2_Cl_2_ (3x) and dried over Na_2_SO_4_. The organics were concentrated, and the crude product (173 mg) was used in the next step without purification.

Step 2:

The step 1 intermediate was used in Procedure A to afford a 63% yield of white foam.

^1^H NMR (400 MHz, CDCl_3_): δ 8.42 (dd, *J =* 4.8, 1.7 Hz, 1H), 7.30 (dd, *J =* 7.7, 1.7 Hz, 1H), 7.05 (t, *J =* 7.7 Hz, 1H), 7.02 (dd, *J =* 7.7, 4.6 Hz, 1H), 6.83 (d, *J =* 7.8 Hz, 1H), 6.76 (d, *J =* 7.6 Hz, 1H), 4.06 (A of AB, *J*_*AB*_ = 15.1 Hz, 1H), 4.07–4.00 (m, 1H), 3.85 (d, *J =* 15.2 Hz, 1H), 3.09–2.94 (m, 4H), 2.88–2.48 (m, 13H), 2.39 (dd, *J =* 13.2, 10.5 Hz, 1H), 2.17–1.81 (m, 7H), 1.74–1.62 (m, 1H), 1.57–1.35 (m, 4H); ^13^C NMR (151 MHz, CDCl_3_): δ 158.54, 150.59, 146.55, 136.68, 136.40, 133.75, 129.86, 125.88, 122.14, 121.79 (t, *J =* 241.5 Hz), 121.28, 117.00, 61.33, 57.98, 54.24, 52.10, 48.78, 48.75 (dd, *J =* 12.0, 6.6 Hz), 41.76, 34.69 (t, *J =* 22.6 Hz), 30.90, 29.79, 29.29, 28.84, 27.11, 21.89; LC-MS (ESI-API, 254 nm) 50–95% MeOH in H_2_O (0.1% HCO_2_H), 3 min, 1.00 mL/min, C18 (Agilent Zorbax XDB-18, 50 mm × 4.6 mm, 3.5 μm), *m*/*z* = 484.2 (M + H), 242.6 (M/2 + H), t = 0.537 min.

**1-(4-(((*(R)*-5-Morpholino-1,2,3,4-tetrahydroisoquinolin-3-yl)­methyl)­(*(S)*-5,6,7,8-tetrahydroquinolin-8-yl)­amino)­butyl)­urea (40)** was synthesized in the following steps.

Step 1.

##### *tert*-Butyl*(R)*-3-(((4-aminobutyl)­(*(S)*-5,6,7,8-tetrahydroquinolin-8-yl)­amino)­methyl)-5-morpholino-3,4-dihydroisoquinoline-2­(1*H*)-carboxylate

*tert*-Butyl *(R)*-3-(((4-(1,3-dioxoisoindolin-2-yl)­butyl)­(*(S)*-5,6,7,8-tetrahydroquinolin-8-yl)­amino)­methyl)-5-morpholino-3,4-dihydroisoquinoline-2­(1*H*)-carboxylate (**37**) was dissolved in 10 mL MeOH, and 0.66 g (13.24 mmol) of hydrazine hydrate was added at room temperature and stirred overnight. The solvent was evaporated. Then, the residue was dissolved in water and extracted with DCM three times. The combined organic layers were dried over anhydrous MgSO_4_, filtered off, and evaporated. It was used for the next step without further purification, yielding 0.67 g (92.1% yield).

^1^H NMR (400 MHz, CDCl_3_): δ 8.30 (s, 1H), 7.27 (d, *J =* 7.7 Hz, 1H), 7.11 (t, *J =* 7.8 Hz, 1H), 6.96 (t, *J =* 6.4 Hz, 1H), 6.88–6.81 (m, 1H), 6.78 (d, *J =* 10.0 Hz, 1H), 4.67 (t, *J =* 16.5 Hz, 2H), 4.39 (s, 1H), 4.13 (d, *J =* 17.0 Hz, 1H), 3.87 (d, *J =* 30.1 Hz, 6H), 3.65 (s, 1H), 3.48–3.21 (m, 1H), 3.06 (s, 2H), 2.98–2.52 (m, 10H), 2.45 (d, *J =* 11.9 Hz, 1H), 2.31–2.05 (m, 2H), 1.93 (s, 1H), 1.80–1.50 (m, 3H), 1.48 (s, 9H), 1.44–1.10 (m, 1H).

Step 2.

##### *tert*-butyl*(R)*-5-Morpholino-3-(((*(S)*-5,6,7,8-tetrahydroquinolin-8-yl)­(4-ureidobutyl)­amino)­methyl)-3,4-dihydroisoquinoline-2­(1*H*)-carboxylate

0.68 g (1.236 mmol) *tert*-butyl *(R)*-3-(((4-aminobutyl)­(*(S)*-5,6,7,8-tetrahydroquinolin-8-yl)­amino)­methyl)-5-morpholino-3,4-dihydroisoquinoline-2­(1*H*)-carboxylate, 0.474 mL (2.7212 mmol) N,N-diisopropylethylamine, and 0.2009 mL (1.4843 mmol) of trimethylsilylisocyanate were dissolved in 10 mL of THF and stirred at room temperature overnight. The reaction was poured into water, and the aqueous phase was extracted with DCM. The combined organic layers were dried over anhydrous MgSO_4_, filtered off, and evaporated. It was purified with column chromatography, starting with DCM and increasing the polarity with MeOH to 10%MeOH in DCM, to give 0.53 g (72.3% yield) of a colorless oil.

^1^H NMR (400 MHz, CDCl_3_): δ 8.26 (d, *J =* 4.8 Hz, 1H), 7.24 (s, 1H), 7.11 (t, *J =* 7.8 Hz, 1H), 6.93 (dd, *J =* 7.6, 4.7 Hz, 1H), 6.88–6.83 (m, 1H), 6.77 (d, *J =* 7.7 Hz, 1H), 5.99 (s, 1H), 4.90–4.46 (m, 4H), 4.45–4.32 (m, 1H), 4.25 (d, *J =* 17.1 Hz, 1H), 4.16–3.76 (m, 5H), 3.59–3.31 (m, 3H), 3.31–2.87 (m, 4H), 2.87–2.45 (m, 3H), 2.38–2.05 (m, 3H), 2.05–1.55 (m, 4H), 1.46–1.50 (m, 12H), 1.02 (s, 1H).

Step 3.

The product from step 2 was used in Procedure A to afford a 77% yield of a colorless foam.

^1^H NMR (500 MHz, CDCl_3_): δ 8.58–8.34 (m, 1H), 7.39–7.29 (m, 1H), 7.15–7.04 (m, 2H), 6.87 (d, *J =* 7.8 Hz, 1H), 6.73 (dd, *J =* 7.6, 1.1 Hz, 1H), 6.37 (s, 1H), 4.77 (s, 2H), 4.10–3.90 (m, 2H), 3.90–3.72 (m, 4H), 3.47 (d, *J =* 15.4 Hz, 1H), 3.42–3.37 (m, 1H), 3.34–3.11 (m, 2H), 3.06 (dd, *J =* 13.4, 3.0 Hz, 1H), 3.01–2.88 (m, 2H), 2.86–2.75 (m, 2H), 2.71–2.61 (m, 5H), 2.51 (dd, *J =* 13.3, 10.5 Hz, 1H), 2.37 (s, 1H), 2.25–2.11 (m, 1H), 2.11–1.89 (m, 2H), 1.76–1.66 (m, 2H), 1.61 (s, 4H); ^13^C NMR (126 MHz, CDCl_3_): δ 159.39, 158.37, 151.04, 146.73, 137.04, 133.98, 129.41, 126.36, 122.06, 121.82, 117.05, 67.42, 61.57, 56.96, 56.89, 53.86, 52.24, 51.82, 47.92, 47.88, 40.33, 29.86, 29.53, 26.49, 26.43, 25.20, 22.03; LC-MS (ESI-API, 254 nm) 75–95% MeOH in H_2_O (0.1% HCO_2_H), 3 min, 1.00 mL/min, C18 (Agilent Zorbax XDB-18, 50 mm × 4.6 mm, 3.5 μm), *m*/*z* = 493.2 (M + 1), t = 0.590 min, purity: ≥ 95%; HRMS calculated for C_28_H_41_O_2_N_6_ 493.32855; found 498.32870 [M + H].

##### *tert*-Butyl 4-(3-oxopropyl)­piperidine-1-carboxylate (**41**)

4-(3-Hydroxy-propyl)-piperidine-1-carboxylicacid *tert*-butylester (0.28 g, 1.15 mmol) was dissolved in 5 mL of DCM, and Dess–Martin reagent (0.732 g, 1.73 mmol) was added to the reaction mixture and stirred overnight. Thirty mL of saturated sodium thiosulfate and 30 mL of saturated sodium bicarbonate, and some DCM, were added and left for stirring for 30 min until the phases were clear. The aqueous phase was extracted with DCM two times, and the combined organic layers were dried over anhydrous MgSO_4_, filtered off, and evaporated. The residue was used as is without further purification.

***(S)*-N-((*(R)*-5-Morpholino-1,2,3,4-tetrahydroisoquinolin-3-yl)­methyl)-N-(3-(piperidin-4-yl)­propyl)-5,6,7,8-tetrahydroquinolin-8-amine (42)** was synthesized with the following steps.

Step 1.

##### *tert*-Butyl*(R)*-3-(((3-(1-(*tert*-butoxycarbonyl)­piperidin-4-yl)­propyl)­(*(S)*-5,6,7,8-tetrahydroquino-lin-8-yl)­amino)­methyl)-5-morpholino-3,4-dihydroisoquinoline-2­(1H)-carboxylate

It was synthesized with Procedure C, starting with **34** and **41,** and adding titanium­(IV) isopropoxide. It was purified with column chromatography using DCM-DCM:MeOH:NH_3_ (7N in MeOH) (90:10:2) to afford a 25% yield of a colorless oil.

^1^H NMR (400 MHz, CDCl_3_): δ 8.30 (s, 1H), 7.32–7.25 (m, 1H), 7.11 (t, *J =* 7.7 Hz, 1H), 6.97 (d, *J =* 6.5 Hz, 1H), 6.90–6.80 (m, 1H), 6.78 (d, *J =* 10.8 Hz, 1H), 4.67 (t, *J =* 16.9 Hz, 2H), 4.54–3.74 (m, 8H), 3.64 (s, 1H), 3.47–3.22 (m, 1H), 3.06 (s, 2H), 2.83–2.49 (m, 8H), 2.43 (ddd, *J =* 13.5, 8.8, 4.8 Hz, 1H), 2.26–1.99 (m, 2H), 1.93 (s, 1H), 1.57 (s, 11H), 1.48 (s, 9H), 1.43 (s, 9H).

Step 2.

Procedure A was used, staring with the Step 1 intermediate. It was purified with column chromatography using DCM-DCM:MeOH:NH_3_ (7N in MeOH) (9:1:0.2) to give (61% yield) a colorless foam.

^1^H NMR (500 MHz, CDCl_3_): δ 8.43 (dd, *J =* 4.8, 1.7 Hz, 1H), 7.3**5–7.30** (m, 1H), 7.10 (t, *J =* 7.7 Hz, 1H), 7.06–7.01 (m, 1H), 6.86 (dd, *J =* 7.9, 1.2 Hz, 1H), 6.79 (dd, *J =* 7.6, 1.1 Hz, 1H), 4.13 (d, *J =* 15.2 Hz, 1H), 4.06 (dd, *J =* 10.2, 6.1 Hz, 1H), 4.00–3.69 (m, 7H), 3.18–2.89 (m, 7H), 2.89–2.81 (m, 1H), 2.81–2.72 (m, 1H), 2.72–2.38 (m, 8H), 2.18 (dd, *J =* 16.2, 10.8 Hz, 1H), 2.12–2.02 (m, 1H), 1.98 (m, 1H), 1.89 (m, 1H), 1.80–1.65 (m, 3H), 1.65–1.43 (m, 1H), 1.33–1.03 (m, 5H); ^**13**^C NMR (126 MHz, CDCl_3_) δ 158.87, 151.10, 146.66, 136.56, 136.29, 133.94, 129.95, 126.20, 122.10, 121.42, 116.86, 67.46, 61.75, 57.93, 54.45, 52.60, 52.21, 48.55, 45.85, 35.53, 34.43, 32.18, 32.10, 29.84, 29.41, 26.91, 22.00; LC-MS (ESI-API, 254 nm) 75–95% MeOH in H_2_O (0.1% HCO_2_H), 3 min, 1.00 mL/min, C18 (Agilent Zorbax XDB-18, 50 mm × 4.6 mm, 3.5 μm), *m*/*z* = 504.4 (M + 1), t = 0.0,438 min, purity: **≥** 95%; HRMS calculated for C_31_H_45_N_5_O 504.36969, found 504.36921 [M + H].

**4-((4,4-Difluorocyclohexyl)­(methyl)­amino)­butanal (43)** was synthesized in the following steps.

Step 1.

A 250 mL round-bottom flask equipped with a magnetic stir bar and rubber septum was charged with 5.00 g of the amine (31.0 mmol, 1 equiv), 4.16 g of 4,4-difluorocyclohexan-1-one (31.0 mmol, 1 equiv), and 120 mL of DCE. Then, 7.89 g of STAB (37.2 mmol, 1.2 equiv) was added. After stirring at rt for 2 h, the reaction mixture was quenched by the addition of sat. NaHCO_3_ solution, extracted with DCM (3x), and dried over Na_2_SO_4_. The organics were concentrated, and the crude product was used in the next step without purification.

^1^H NMR (400 MHz, CDCl_3_): δ 4.49 (t, *J =* 5.6 Hz, 1H), 3.64 (dq, *J =* 9.4, 7.1 Hz, 2H), 3.49 (dq, *J =* 9.4, 7.1 Hz, 2H), 2.62 (t, *J =* 7.2 Hz, 2H), 2.65–2.52 (m, 1H), 2.16–2.02 (m, 3H), 1.91–1.61 (m, 6H), 1.59–1.41 (m, 4H), 1.20 (t, *J =* 7.1 Hz, 6H).

Step 2.

A 250 mL round-bottom flask equipped with a magnetic stir bar and rubber septum was charged with 7.79 g of the amine (27.9 mmol, 1 equiv), 1.67 g of paraformaldehyde (55.8 mmol, 2 equiv), and 110 mL of DCE. Then, 11.8 g of STAB (55.8 mmol, 2 equiv) was added. After stirring at rt for 12 h, no product was observed by LCMS. Then, 1.6 mL of AcOH (27.9 mmol, 1 equiv) was added, and stirring was continued for 12 h. Some product was formed, but the reaction did not go to completion even if more paraformaldehyde and STAB were added. Then, 2.50 mL of 37% formalin (33.5 mmol, 1.2 equiv) was added, and the reaction was done in 1 h. The reaction mixture was quenched by the addition of sat. NaHCO_3_ and solution, extracted with DCM (3x), and dried over Na_2_SO_4_. The organics were concentrated, and the crude product was purified on a silica gel column (120 g) using 0 to 10% MeOH in EA as the eluent, affording 3.21 g of the product (minor impurity) as a brown liquid and 3.97 g of the product as a brown liquid (88% combined yield).

^1^H NMR (400 MHz, CDCl_3_): δ 4.50 (t, *J =* 5.6 Hz, 1H), 3.64 (dq, *J =* 9.4, 7.0 Hz, 2H), 3.49 (dq, *J =* 9.4, 7.1 Hz, 2H), 2.51–2.40 (m, 1H), 2.42 (t, *J =* 7.4 Hz, 2H), 2.24 (s, 3H), 2.19–2.06 (m, 2H), 1.81–1.46 (m, 10H), 1.20 (t, *J =* 7.1 Hz, 6H).

Step 3.

A 100 mL round-bottom flask equipped with a stir bar, reflux condenser, and septum was charged with 1.50 g of the acetal (5.11 mmol, 1 equiv), 1.96 mL of 6 M HCl (11.8 mmol, 2.3 equiv), and 13.0 mL of acetone. After stirring at 80 °C for 3 h, the reaction mixture was concentrated, quenched by the addition of sat. NaHCO_3_ solution, extracted with diethyl ether (3x), and dried over Na_2_SO_4_. The crude material (476 mg) was dissolved in hexanes, filtered, and the organics were removed by distillation under vacuum, affording 452 mg (40% yield) of the product as a yellow oil.

^1^H NMR (400 MHz, CDCl_3_): δ 9.72 (td, *J =* 1.9, 0.7 Hz, 1H), 2.49–2.40 (m, 5H), 2.20 (s, 3H), 2.17–2.07 (m, 2H), 1.85–1.51 (m, 8H).

**N^1^-(4,4-Difluorocyclohexyl)-N^1^-methyl-N^4^-((*(R)*-5-morpholino-1,2,3,4-tetrahydroisoquinolin-3-yl)­methyl)-N^4^-(*(S)*-5,6,7,8-tetrahydroquinolin-8-yl)­butane-1,4-diamine (44)** was synthesized in the following steps.

Step 1.

##### *tert*-Butyl*(R)*-3-(((4-((4,4-difluorocyclohexyl)­(methyl)­amino)­butyl)­(*(S)*-5,6,7,8-tetrahydroquino-lin-8-yl)­amino)­methyl)-5-morpholino-3,4-dihydroisoquinoline-2­(1*H*)-carboxylate

Procedure C was used, starting with **34** and **43**. Crude material was purified with column chromatography, starting with DCM and increasing the polarity with DCM:MeOH:NH_3_ (7N in MeOH) (90:10:2), to give a 26% yield of a yellowish foam.

^1^H NMR (400 MHz, CDCl_3_): δ 8.29 (s, 1H), 7.26 (t, *J =* 6.5 Hz, 1H), 7.11 (t, *J =* 7.8 Hz, 1H), 6.97 (d, *J =* 7.0 Hz, 1H), 6.84 (d, *J =* 7.9 Hz, 1H), 6.77 (d, *J =* 11.6 Hz, 1H), 4.66 (t, *J =* 15.8 Hz, 2H), 4.38 (s, 1H), 4.12 (d, *J =* 17.0 Hz, 1H), 3.86 (d, *J =* 28.9 Hz, 5H), 3.35 (dd, *J =* 41.2, 15.8 Hz, 1H), 3.06 (s, 2H), 2.65 (dd, *J =* 35.6, 18.6 Hz, 6H), 2.44 (s, 1H), 2.29 (s, 1H), 2.23–2.15 (m, 3H), 2.11 (s, 3H), 1.94 (s, 1H), 1.66 (d, *J =* 55.3 Hz, 12H), 1.48 (s, 9H), 1.22 (d, *J =* 56.3 Hz, 3H).

Step 2.

Procedure A was used, starting with step 1 intermediate, to afford a 67% yield of an off-white foam.

^1^H NMR (500 MHz, CDCl_3_): δ 8.42 (d, *J =* 4.8, 1.7 Hz, 1H), 7.34 (d, *J =* 7.7, 1.8, 0.9 Hz, 1H), 7.11 (t, *J =* 7.8 Hz, 1H), 7.05 (dd, *J =* 7.6, 4.7, 0.6 Hz, 1H), 6.87 (d, *J =* 7.9, 1.2 Hz, 1H), 6.79 (d, *J =* 7.6, 1.1 Hz, 1H), 4.16 (d, *J =* 15.3 Hz, 1H), 4.10 (dd, *J =* 10.2, 6.3 Hz, 1H), 3.96 (d, *J =* 15.3 Hz, 1H), 3.87–3.67 (m, 6H), 3.11 (dd, *J =* 13.5, 2.9 Hz, 1H), 3.06–2.93 (m, 4H), 2.88 (dd, *J =* 16.2, 3.2 Hz, 1H), 2.83–2.63 (m, 5H), 2.63–2.53 (m, 2H), 2.52–2.42 (m, 1H), 2.37 (t, *J =* 6.6 Hz, 2H), 2.35–2.22 (m, 1H), 2.18–2.03 (m, 3H), 2.04–1.84 (m, 3H), 1.83–1.62 (m, 7H), 1.62–1.53 (m, 2H), 1.53–1.32 (m, 1H); ^13^C NMR (126 MHz, CDCl_3_): δ 158.80, 151.08, 146.59, 136.77, 134.02, 129.60, 126.37, 123.17 (t, *J*: 96 Hz), 121.99, 121.53, 116.96, 77.33, 77.08, 76.83, 67.43, 61.96, 60.35, 57.51, 53.77, 52.72, 52.19, 37.84, 32.85­(d, *J*:10 Hz), 32.66 (d, *J*:10 Hz), 29.38, 28.97, 27.75, 25.56, 24.08 (d, *J*:3.5 Hz), 21.97; HRMS calculated for C_27_H_38_N_4_O 582.39646; found 582.39659 [M + H].

***(S)*-*N*-Methyl-*N*-((*(R)*-5-morpholino-1,2,3,4-tetrahydroisoquinolin-3-yl)­methyl)-5,6,7,8-tetrahydroquinolin-8-amine (45)** was synthesized through the following steps.

Step 1.

##### *tert*-Butyl*(R)*-3-((methyl­(*(S)*-5,6,7,8-tetrahydroquinolin-8-yl)­amino)­methyl)-5-morpholino-3,4-dihydroisoquinoline-2­(1*H*)-carboxylate

Procedure C was used, starting with **34** and HCHO, to afford a 73% yield of a white foam.

^1^H NMR (400 MHz, CDCl_3_): δ 8.24 (s, 1H), 7.30 (d, 1H, J = 8 Hz), 7.08 (t, 1H, J = 8 Hz), 6.98 (t, 1H, J = 5 Hz), 6.90 (d, 1H, J = 7 Hz), 6.83 (d, 1H, J = 8 Hz), 4.59 (m, 1H), 4.55 (d, 1H, J = 17 Hz), 4.40 (m, 1H), 3.88 (m, 3H), 3.82 (m, 2H), 3.55 (s, 1H), 3.23 (d, 1H, J = 16 Hz), 3.01 (m, 2H), 2.79 (q, 2H, J = 7 Hz), 2.76 (m, 2H), 2.64 (m, 2H), 2.35 (m, 1H), 2.25 (s, 3H), 1.95 (m, 1H), 1.87 (m, 2H), 1.48 (s, 9H); MS: 493.3 *m*/*z* (M+H)^+^.

Step 2.

Procedure A was used, starting with *tert*-butyl *(R)*-3-((methyl­(*(S)*-5,6,7,8-tetrahydroquinolin-8-yl)­amino)­methyl)-5-morpholino-3,4-dihydroisoquinoline-2­(1*H*)-carboxylate to afford a 63% yield of a white foam.

^1^H NMR (400 MHz, CDCl_3_): δ 8.44 (dd, 1H, J = 1, 4 Hz), 7.34 (d, 1H, J = 7 Hz), 7.10 (t, 1H, J = 8 Hz), 7.05 (dd, 1H, J = 5, 8 Hz), 6.86 (d, 1H, J = 8 Hz), 6.77 (d, 1H, J = 8 Hz), 4.09 (d, 1H, J = 16 Hz), 3.97 (m, 2H), 3.79 (m, 4H), 2.98 (m, 2H), 2.83 (m, 4H), 2.69 (m, 4H), 2.59 (m, 1H), 2.47 (s, 3H), 2.22 (m, 1H), 1.98 (m, 3H), 1.71 (m, 1H).

^13^C NMR (101 MHz, CDCl_3_): δ 158.01, 151.07, 146.78, 136.73, 136.05, 133.88, 129.83, 126.17, 121.98, 121.57, 116.78, 74.79, 67.45, 64.47, 59.81, 52.21, 51.66, 48.34, 40.89, 29.90, 29.21, 21.28; LC-MS: 100% @ 0.449 min for 393.2 *m*/*z* (M+H)^+^; HRMS: calculated for C_24_H_33_ON_4_ 393.26489; found 393.26445 [M + H].

***(S)*-N-Ethyl-N-((*(R)*-5-morpholino-1,2,3,4-tetrahydroisoquinolin-3-yl)­methyl)-5,6,7,8-tetrahydroquinolin-8-amine (46)** was synthesized through the following steps.

Step 1.

##### *tert*-Butyl*(R)*-3-((ethyl­(*(S)*-5,6,7,8-tetrahydroquinolin-8-yl)­amino)­methyl)-5-morpholino-3,4-dihydroisoquinoline-2­(1H)-carboxylate

Procedure C was used, starting with **34** and acetaldehyde. The crude material was purified with column chromatography, starting with DCM and increasing the polarity with DCM:MeOH:NH_3_ (7N in MeOH) (90:10:2), which gave the product in 86% yield as a yellowish foam.

^1^H NMR (400 MHz, CDCl_3_): δ 8.31 (d, 1H), 7.29 (d, *J =* 7.7 Hz, 1H), 7.11 (t, *J =* 7.8 Hz, 1H), 6.99 (q, *J =* 8.9 Hz, 1H), 6.85 (d, 1H), 6.76 (d, 1H), 4.09 (d, 1H), 3.96–3.79 (m, 4H), 3.45–3.29 (m, 1H), 3.06 (q, *J =* 8.7, 6.2 Hz, 2H), 2.77–2.69 (m, 2H), 2.69–2.55 (m, 4H), 2.46–2.32 (m, 1H), 1.49 (s, 9H), 1.41–1.35 (m, 1H), 1.31–1.22 (m, 2H), 1.23–1.15 (m, 3H), 0.93 (t, 3H).

Step 2.

Procedure A was used, starting with *tert*-butyl *(R)*-3-((ethyl­(*(S)*-5,6,7,8-tetrahydroquinolin-8-yl)­amino)­methyl)-5-morpholino-3,4-dihydroisoquinoline-2­(1*H*)-carboxylate to afford a 48% yield of an off-white foam.

^1^H NMR (500 MHz, CDCl_3_): δ 8.44 (d, *J =* 4.9, 1.7 Hz, 1H), 7.35 (d, *J =* 7.7 Hz, 1H), 7.13 (t, *J =* 7.7 Hz, 1H), 7.07 (q, *J =* 7.7, 4.7 Hz, 1H), 6.89 (d, *J =* 7.8 Hz, 1H), 6.81 (d, *J =* 7.6 Hz, 1H), 4.13–4.09 (m, 1H), 3.87–3.71 (m, 4H), 3.13 (d, *J =* 13.4 Hz, 1H), 3.04–2.97 (m, 2H), 2.90 (d, *J =* 16.9 Hz, 1H), 2.83–2.75 (m, 1H), 2.75–2.64 (m, 5H), 2.11–2.04 (m, 1H), 2.04–1.95 (m, 2H), 1.95–1.87 (m, 1H), 1.08 (t, *J =* 7.0 Hz, 3H); ^13^C NMR (101 MHz, CDCl_3_): δ 158.76, 151.06, 146.57, 136.86, 134.01, 129.42, 126.41, 121.97, 121.57, 117.02, 67.42, 61.90, 56.74, 52.66, 52.17, 29.33, 28.41, 21.82, 15.12; HRMS: calculated for C_24_H_34_ON_4_ 407.27326; found 407.27454 [M + H].

***(S)*** **-N-((*(R)*-5-Morpholino-1,2,3,4-tetrahydroisoquinolin-3-yl)­methyl)-N-propyl-5,6,7,8-tetrahydroquinolin-8-amine (47)** was synthesized in the following steps.

Step 1.

##### *tert*-Butyl*(R)*-5-morpholino-3-((propyl­(*(S)*-5,6,7,8-tetrahydroquinolin-8-yl)­amino)­methyl)-3,4-dihydroisoquinoline-2­(1*H*)-carboxylate

Procedure C was used, starting with **34** and propionaldehyde. Crude material was purified with column chromatography, starting with DCM and increasing the polarity with DCM:MeOH:NH_3_ (7N in MeOH) (90:10:2), to give a 67% yield of a yellowish foam.

^1^H NMR (400 MHz, CDCl_3_): δ 8.30 (s, 1H), 7.27 (d, *J =* 7.8 Hz, 1H), 7.10 (t, *J =* 7.8 Hz, 1H), 7.01–6.68 (m, 3H), 4.67 (d, *J =* 15.4 Hz, 1H), 4.13 (d, *J =* 17.0 Hz, 1H), 3.87 (d, *J =* 30.9 Hz, 5H), 3.48–3.22 (m, 1H), 3.18–2.54 (m, 11H), 2.43 (d, *J =* 14.0 Hz, 1H), 2.16 (s, 2H), 1.93 (s, 1H), 1.74 (d, *J =* 12.2 Hz, 1H), 1.48 (s, 11H), 0.77 (d, *J =* 7.0 Hz, 3H).

Step 2.

Procedure A was used, starting with *tert*-butyl *(R)*-5-morpholino-3-((propyl­(*(S)*-5,6,7,8-tetrahydroquinolin-8-yl)­amino)­methyl)-3,4-dihydroisoquinoline-2­(1*H*)-carboxylate to afford a 68% yield of a white foam.

^1^H NMR (500 MHz, CDCl_3_): δ 8.44 (d, *J =* 4.7 Hz, 1H), 7.31 (d, *J =* 7.7 Hz, 1H), 7.09 (t, *J =* 7.7 Hz, 1H), 7.03 (dd, *J =* 7.7, 4.7 Hz, 1H), 6.85 (d, *J =* 7.8 Hz, 1H), 6.78 (d, *J =* 7.6 Hz, 1H), 4.13–4.03 (m, 2H), 3.94 (d, *J =* 15.2 Hz, 1H), 3.85–3.78 (m, 2H), 3.78–3.71 (m, 2H), 3.11–3.02 (m, 2H), 3.02–2.95 (m, 2H), 2.84 (dd, *J =* 16.2, 3.1 Hz, 1H), 2.77 (ddd, *J =* 16.3, 11.0, 4.9 Hz, 1H), 2.71–2.61 (m, 4H), 2.54 (dt, *J =* 12.8, 7.7 Hz, 1H), 2.38 (t, *J =* 13.1, 10.5 Hz, 1H), 2.19–2.03 (m, 2H), 2.02–1.94 (m, 1H), 1.96–1.85 (m, 1H), 1.76–1.64 (m, 1H), 1.52 (ddq, *J =* 21.1, 13.9, 7.2 Hz, 2H), 0.88 (t, *J =* 7.3 Hz, 3H); ^13^C NMR (126 MHz, CDCl_3_): δ 159.04, 151.08, 146.68, 136.85, 136.45, 133.91, 130.19, 126.07, 122.10, 121.32, 116.67, 77.37, 77.12, 76.86, 67.48, 61.47, 57.95, 56.59, 52.37, 52.18, 48.93, 30.21, 29.79, 29.46, 23.03, 22.04, 11.86; HRMS calculated for C_27_H_38_N_4_O 421.29485; found 421.29542 [M + H].

***(S)*-N-Butyl-N-((*(R)*-5-morpholino-1,2,3,4-tetrahydroisoquinolin-3-yl)­methyl)-5,6,7,8-tetrahydroquinolin-8-amine (48)** was synthesized by the following steps.

Step 1.

##### *tert*-Butyl*(R)*-3-((butyl­(*(S)*-5,6,7,8-tetrahydroquinolin-8-yl)­amino)­methyl)-5-morpholino-3,4-dihydroisoquinoline-2­(1*H*)-carboxylate

Procedure C was used, starting with **34** and butyraldehyde. Crude material was purified with column chromatography, starting with DCM and increasing the polarity with DCM:MeOH:NH_3_ (7N in MeOH) (90:10:2), to give a 50% yield of a yellowish foam.

^1^H NMR (400 MHz, CDCl_3_): δ 8.30 (s, 1H), 7.27 (d, *J =* 7.6 Hz, 1H), 7.10 (t, *J =* 7.8 Hz, 1H), 6.96 (dd, *J =* 7.6, 4.7 Hz, 1H), 6.84 (d, *J =* 7.9 Hz, 1H), 6.79–6.74 (m, 1H), 4.67–4.64 (m, 1H), 4.38 (s, 1H), 4.11 (d, *J =* 17.2 Hz, 1H), 3.98–3.74 (m, 4H), 3.68–3.63 (m, 1H), 3.46–3.20 (m, 1H), 3.12–3.00 (m, 2H), 2.78–2.69 (m, 5H), 2.67–2.56 (m, 2H), 2.53–2.34 (m, 1H), 2.30–2.18 (m, 1H), 2.13–2.09 (m, 1H), 1.95–1.90 (m, 1H), 1.78–1.72 (m, 2H), 1.48 (s, 9H), 1.41–1.28 (m, 1H), 0.98–0.87 (m, 3H), 0.81 (t, *J =* 7.1 Hz, 3H).

Step 2.

Procedure A was used, starting with *tert*-butyl *(R)*-3-((butyl­(*(S)*-5,6,7,8-tetrahydroquinolin-8-yl)­amino)­methyl)-5-morpholino-3,4-dihydroisoquinoline-2­(1*H*)-carboxylate to afford a 52% yield of an off-white foam.

^1^H NMR (500 MHz, CDCl_3_): δ 8.38 (d, *J =* 4.7, 1.7 Hz, 1H), 7.42 (d, 1H), 7.22 (t, *J =* 7.8 Hz, 1H), 7.12 (ddd, *J =* 7.7, 4.8, 0.8 Hz, 1H), 6.98 (dd, *J =* 8.1, 1.1 Hz, 1H), 6.87 (dd, *J =* 7.7, 1.1 Hz, 1H), 4.58 (d, *J =* 15.7 Hz, 1H), 4.25 (d, *J =* 15.7 Hz, 1H), 4.14 (dd, *J =* 10.6, 6.2 Hz, 1H), 3.90–3.72 (m, 4H), 3.29 (d, *J =* 6.2 Hz, 3H), 3.08 (dd, *J =* 16.9, 3.3 Hz, 1H), 3.05–2.97 (m, 2H), 2.92 (dd, *J =* 16.9, 9.5 Hz, 1H), 2.84–2.71 (m, 4H), 2.64–2.54 (m, 1H), 2.49–2.45 (m, 1H), 2.16–2.08 (m, 1H), 2.02–1.95 (m, 1H), 1.87 (tdd, *J =* 13.0, 10.5, 2.8 Hz, 1H), 1.80–1.69 (m, 1H), 1.40–1.29 (m, 1H), 1.31–1.22 (m, 1H), 1.24 (s, 1H), 1.22–1.10 (m, 2H), 0.76 (t, *J =* 7.3 Hz, 3H); ^13^C NMR (126 MHz, CDCl_3_): δ 158.26, 151.20, 146.14, 137.96, 134.41, 129.69, 127.59, 127.29, 122.27, 121.69, 118.30, 77.29, 77.04, 76.78, 67.32, 64.18, 54.17, 52.23, 43.62, 31.49, 29.11, 25.27, 21.76, 20.23, 13.86; HRMS calculated for C_27_H_38_N_4_O 435.31184; found 435.31121 [M + H].

***(S)*-N-((*(R)*-5-Morpholino-1,2,3,4-tetrahydroisoquinolin-3-yl)­methyl)-N-(4,4,4-trifluorobutyl)-5,6,7,8-tetrahydroquinolin-8-amine (49)** was synthesized by the following steps.

Step 1.

##### *tert*-Butyl*(R)*-5-morpholino-3-(((*(S)*-5,6,7,8-tetrahydroquinolin-8-yl)­(4,4,4-trifluorobutyl)­amino)­methyl)-3,4-dihydroisoquinoline-2­(1*H*)-carboxylate

Procedure C was used, starting with **34** and 4,4,4-trifluorobutanal. The crude product was purified by column chromatography, starting with DCM and increasing the polarity with DCM:MeOH:NH_3_ (7N in MeOH) (90:10:2), to give a 89% yield of a colorless oil.

^1^H NMR (400 MHz, CDCl_3_): δ 8.35 (s, 1H), 7.33–7.27 (m, 1H), 7.15 (t, *J =* 7.8 Hz, 1H), 7.02–6.97 (m, 1H), 6.88 (dd, *J =* 8.0, 1.1 Hz, 1H), 6.83 (d, *J =* 9.4 Hz, 1H), 4.72 (d, *J =* 16.8 Hz, 2H), 4.21 (d, *J =* 17.1 Hz, 1H), 3.89 (d, *J =* 31.7 Hz, 5H), 3.58–3.36 (m, 1H), 3.16–3.00 (m, 2H), 2.85–2.64 (m, 5H), 2.64–2.45 (m, 1H), 2.41 (s, 1H), 2.34–2.14 (m, 1H), 2.14–1.85 (m, 2H), 1.63 (s, 6H), 1.51 (s, 10H).

Step 2.

Procedure A was used, starting with *tert*-butyl *(R)*-5-morpholino-3-(((*(S)*-5,6,7,8-tetrahydroquinolin-8-yl)­(4,4,4-trifluorobutyl)­amino)­methyl)-3,4-dihydroisoquinoline-2­(1*H*)-carboxylate to afford a 77% yield of a yellowish foam.

^1^H NMR (600 MHz, CDCl_3_): δ 8.46 (dd, *J =* 4.8, 1.7 Hz, 1H), 7.36 (dd, *J =* 7.7, 1.7 Hz, 1H), 7.13 (t, *J =* 7.7 Hz, 1H), 7.08 (dd, *J =* 7.7, 4.7 Hz, 1H), 6.90 (d, *J =* 7.8 Hz, 1H), 6.82 (d, *J =* 7.6 Hz, 1H), 4.16 (d, *J =* 15.3 Hz, 1H), 4.08 (dd, *J =* 10.4, 6.2 Hz, 1H), 3.97 (d, *J =* 15.2 Hz, 1H), 3.89–3.82 (m, 2H), 3.82–3.75 (m, 2H), 3.18–3.10 (m, 1H), 3.08 (dd, *J =* 13.4, 3.2 Hz, 1H), 3.04–2.96 (m, 2H), 2.92 (dd, *J =* 16.2, 3.2 Hz, 1H), 2.86–2.64 (m, 6H), 2.57–2.47 (m, 1H), 2.34–2.07 (m, 4H), 2.06–1.97 (m, 1H), 1.97–1.86 (m, 1H), 1.84–1.66 (m, 3H); ^13^C NMR (151 MHz, CDCl_3_): δ 158.43, 151.12, 146.77, 136.62, 136.36, 133.96, 129.84,127.52 (q, J: 276.0 Hz), 126.26, 122.09, 121.58, 116.89, 67.47, 61.70, 58.04, 53.06, 52.63, 52.23, 48.60, 31.46 (q,*J =* 28.5 Hz), 29.97, 29.38, 28.96, 22.24 (d,*J =* 2.2 Hz), 22.07; LC-MS (ESI-API, 254 nm) 75–95% MeOH in H_2_O (0.1% HCO_2_H), 3 min, 1.00 mL/min, C18 (Agilent Zorbax XDB-18, 50 mm × 4.6 mm, 3.5 μm), *m*/*z* = 489.2 (M + 1), t = 0.496 min, purity: **≥**95%; HRMS calculated for C_27_H_36_ON_4_F_3_ 489.28357; found 489.28310 [M + H].

**N^1^-((*(R)*-5-Morpholino-1,2,3,4-tetrahydroisoquinolin-3-yl)­methyl)-N^1^-(*(S)*-5,6,7,8-tetrahydroquinolin-8-yl)­ethane-1,2-diamine (50)** was synthesized through the following steps.

Step 1.

##### *tert*-Butyl*(R)*-3-(((2-((*tert*-butoxycarbonyl)­amino)­ethyl)­(*(S)*-5,6,7,8-tetrahydroquinolin-8-yl)­amino)­methyl)-5-morpholino-3,4-dihydroisoquinoline-2­(1*H*)-carboxylate

Procedure C was used, starting with **34** and *tert*-butyl N-(2-oxoethyl)­carbamate. It was purified with column chromatography, starting with DCM and increasing the polarity with DCM:MeOH:NH_3_ (7N in MeOH) (90:10:2), to give 0.22 g (85% yield) of a yellow foam.

^1^H NMR (400 MHz, CDCl_3_): δ 8.50 (d, *J =* 29.6 Hz, 1H), 7.31 (s, 1H), 7.15 (t, *J =* 7.8 Hz, 1H), 7.00 (d, *J =* 17.7 Hz, 1H), 6.90–6.75 (m, 2H), 6.57 (d, *J =* 7.0 Hz, 1H), 4.78–4.36 (m, 2H), 4.22 (d, *J =* 16.7 Hz, 1H), 4.16–3.98 (m, 1H), 3.97–3.44 (m, 1H), 3.46–3.26 (m, 1H), 3.02 (s, 2H), 2.74 (d, *J =* 32.3 Hz, 6H), 2.37 (d, *J =* 91.9 Hz, 4H), 2.15–1.84 (m, 1H), 1.84–1.55 (m, 5H), 1.51–1.46 (m, 11H), 1.42 (s, 9H).

Step 2.

Procedure A was used, starting with *tert*-butyl-*(R)*-3-(((2-((*tert*-butoxycarbonyl)­amino)­ethyl)­(*(S)*-5,6,7,8-tetrahydroquinolin-8-yl)­amino)­methyl)-5-morpholino-3,4-dihydroisoquinoline-2­(1*H*)-carboxylate, and the product was crystallized using a 5:1 hexanes-DCM mixture to give a 7% yield of an amorphous solid.

^1^H NMR (500 MHz, CDCl_3_): δ 8.52–8.40 (m, 1H), 7.35 (d, *J =* 7.8 Hz, 1H), 7.17–7.03 (m, 2H), 6.88 (d, *J =* 7.9 Hz, 1H), 6.81–6.68 (m, 1H), 4.15 (d, *J =* 16.1 Hz, 1H), 4.00 (dd, *J =* 10.9, 5.8 Hz, 1H), 3.93–3.66 (m, 4H), 3.27 (s, 1H), 3.15–2.86 (m, 5H), 2.86–2.53 (m, 2H), 2.38 (s, 1H), 2.17 (d, *J =* 35.5 Hz, 1H), 2.00 (s, 1H), 1.92–1.77 (m, 1H), 1.77–1.66 (m, 1H), 1.65–1.45 (m, 1H), 1.39–1.17 (m, 5H), 1.17–0.99 (m, 1H), 0.99–0.94 (m, 1H), 0.87–0.80 (m, 2H); ^13^C NMR (126 MHz, CDCl_3_): δ 151.08, 146.77, 137.58, 136.10, 134.18, 128.67, 126.72, 122.23, 121.81, 117.29, 77.24, 67.36, 63.23, 57.15, 52.95, 52.23, 46.44, 38.63, 37.08, 28.98, 28.53, 25.30, 21.85; LC-MS (ESI-API, 254 nm) 75–95% MeOH in H_2_O (0.1% HCO_2_H), 3 min, 1.00 mL/min, C18 (Agilent Zorbax XDB-18, 50 mm × 4.6 mm, 3.5 μm), *m*/*z* = 422.2 (M + 1), t = 0.414 min, purity: **≥** 95%; HRMS calculated for C_25_H_36_ON_5_ 422.29144; found 422.29135 [M + H].

##### *(S)*-N-(4-((*tert*-Butyldimethylsilyl)­oxy)­butyl)-5,6,7,8-tetrahydroquinolin-8-amine (**52**)

Procedure C was used, starting with **12** and **51,** with addition of titanium isopropoxide. The crude product was purified with column chromatography, starting with DCM and increasing the polarity with DCM:MeOH:NH_3_ (7N in MeOH) (90:10:2), to give 0.55 g (35% yield) of a yellowish oil.

^1^H NMR (500 MHz, CDCl_3_): δ 8.37 (dt, *J =* 4.7, 1.7, 0.8 Hz, 1H), 7.35 (d, *J =* 7.7, 1.8, 0.9 Hz, 1H), 7.04 (dd, *J =* 7.7, 4.7, 0.7 Hz, 1H), 3.79–3.73 (m, 1H), 3.67–3.60 (m, 2H), 2.85–2.66 (m, 4H), 2.48 (s, 1H), 2.18–2.07 (m, 1H), 2.05–1.93 (m, 1H), 1.80–1.66 (m, 2H), 1.66–1.54 (m, 4H), 0.88 (s, 9H), 0.03 (s, 6H).

**4-(((*(R)*-5-Morpholino-1,2,3,4-tetrahydroisoquinolin-3-yl)­methyl)­(*(S)*-5,6,7,8-tetrahydroquinolin-8-yl)­amino)­butan-1-ol (53)** was synthesized through the following steps.

Step 1.

##### *tert*-Butyl*(R)*-3-(((4-((*tert*-butyldimethylsilyl)­oxy)­butyl)­(*(S)*-5,6,7,8-tetrahydroquinolin-8-yl)­amino)­methyl)-5-morpholino-3,4-dihydroisoquinoline-2­(1*H*)-carboxylate

Procedure C was used, starting with **52** and **32,** and the crude material was purified with column chromatography, starting with DCM and increasing the polarity with DCM:MeOH:NH_3_ (7N in MeOH) (90:10:2), to afford a 51% yield of a light brown oil.

^1^H NMR (400 MHz, CDCl_3_): δ 8.29 (s, 1H), 7.27 (dd, *J =* 7.6, 1.7 Hz, 1H), 7.10 (t, *J =* 7.8 Hz, 1H), 6.96 (dd, *J =* 7.7, 4.7 Hz, 1H), 6.87–6.82 (m, 1H), 6.77 (d, *J =* 8.7 Hz, 1H), 4.66 (d, *J =* 16.5 Hz, 2H), 4.41–4.28 (m, 1H), 4.10 (d, *J =* 16.9 Hz, 1H), 3.86 (d, *J =* 29.6 Hz, 6H), 3.70–3.23 (m, 5H), 3.06 (s, 2H), 2.92 (d, *J =* 9.9 Hz, 1H), 2.84–2.54 (m, 3H), 2.46 (dt, *J =* 13.1, 6.2 Hz, 1H), 2.18 (d, *J =* 50.4 Hz, 2H), 1.92 (s, 1H), 1.72 (t, *J =* 11.8 Hz, 1H), 1.62 (s, 2H), 1.52–1.42 (m, 12H), 1.42–1.21 (m, 3H), 0.90–0.79 (m, 12H).

Step 2.

Procedure A was used, starting with *tert*-butyl *(R)*-3-(((4-((*tert*-butyldimethylsilyl)­oxy)­butyl)­(*(S)*-5,6,7,8-tetrahydroquinolin-8-yl)­amino)­methyl)-5-morpholino-3,4-dihydroisoquinoline-2­(1*H*)-carboxylate to afford a 25% yield of an off-white foam.

^1^H NMR (500 MHz, CDCl_3_): δ 8.53–8.24 (m, 1H), 7.45 (s, 1H), 7.17 (q, *J =* 7.7 Hz, 2H), 6.94 (d, *J =* 7.9 Hz, 1H), 6.81 (d, *J =* 7.7 Hz, 1H), 4.48 (d, *J =* 15.9 Hz, 1H), 4.36–3.99 (m, 2H), 3.78 (tddd, *J =* 11.0, 8.9, 5.6, 2.4 Hz, 4H), 3.52 (dq, *J =* 25.1, 6.1 Hz, 2H), 3.26 (d, *J =* 11.7 Hz, 3H), 3.14–2.90 (m, 5H), 2.89–2.50 (m, 6H), 2.25–2.08 (m, 1H), 1.99 (d, *J =* 13.0 Hz, 1H), 1.92–1.81 (m, 1H), 1.80–1.66 (m, 1H), 1.49 (d, *J =* 39.0 Hz, 4H); ^13^C NMR (126 MHz, CDCl_3_): δ 157.82, 151.05, 145.95, 138.27, 134.61, 129.41, 127.58, 127.09, 122.54, 121.63, 118.21, 67.27, 63.80, 62.10, 55.21, 53.79, 53.45, 52.25, 51.56, 43.45, 30.27, 28.97, 25.81, 25.25, 21.67; LC-MS (ESI-API, 254 nm) 75–95% MeOH in H_2_O (0.1% HCO_2_H), 3 min, 1.00 mL/min, C18 (Agilent Zorbax XDB-18, 50 mm × 4.6 mm, 3.5 μm), *m*/*z* = 451.2 (M + 1), t = 0.460 min, purity: **≥** 95%; HRMS calculated for C_27_H_39_N_4_O_2_ 451.30675; found 451.30650 [M + H].

***tert*** **-Butyl 4-(3-bromopropyl)­piperazine-1-carboxylate (54)** was synthesized according to known procedures.^51^

**Benzyl 4-(3-bromopropyl)­piperazine-1-carboxylate (55)** was synthesized according to known procedures.^51^

***tert*-Butyl*(S)*-4-(3-((5,6,7,8-tetrahydroquinolin-8-yl)­amino)­propyl)­piperazine-1-carboxylate (56)** was synthesized according to known procedures.^51^

##### Benzyl-*(S)*-4-(3-((5,6,7,8-tetrahydroquinolin-8-yl)­amino)­propyl)­piperazine-1-carboxylate (**57**)

2.07 g (13.968 mmol) **12**, 4.333 g (12.968 mmol) **55,** and 4.102 g (31.745 mmol) N,N-diisopropyl-N-ethylamine were mixed in 40 mL acetonitrile, and stirred overnight. Saturated sodium bicarbonate solution (100 mL) and EA (100 mL) were added to the reaction mixture and stirred for another 30 min. The phases were separated, and the aqueous phase was extracted with EA two times. The combined organic layer was dried over anhydrous MgSO_4_, filtered off, and evaporated. The compound was purified with column chromatography starting with DCM and increasing the polarity with DCM:MeOH:NH_3_ (7N in MeOH) (90:10:2), to yield 5.3 g (28% yield) of a yellow sticky solid.

^1^H NMR (400 MHz, CDCl_3_): δ 8.36 (d, *J =* 4.7 Hz, 1H), 7.41 (d, *J =* 7.8 Hz, 1H), 7.24–7.24 (m, 3H), 7.15–7.08 (m, 1H), 7.01–6.95 (m, 1H), 6.86 (d, *J =* 7.7 Hz, 1H), 4.58 (d, *J =* 15.7 Hz, 1H), 4.41–3.96 (m, 2H), 3.81 (td, *J =* 6.3, 3.1 Hz, 3H), 3.30 (s, 2H), 3.04 (dd, *J =* 29.1, 14.4 Hz, 3H), 2.74 (dd, *J =* 12.3, 6.5 Hz, 3H), 2.52 (d, *J =* 47.0 Hz, 1H), 2.13 (d, *J =* 19.9 Hz, 1H), 2.02–1.81 (m, 2H), 1.24 (s, 1H), 1.15 (q, *J =* 7.2 Hz, 2H), 0.76 (t, *J =* 7.2 Hz, 3H).

***(S)*-N-((*(R)*-5-morpholino-1,2,3,4-tetrahydroisoquinolin-3-yl)­methyl)-N-(3-(piperazin-1-yl)­propyl)-5,6,7,8-tetrahydroquinolin-8-amine (58)** was synthesized in the following steps.

Step 1.

##### *tert*-Butyl*(R)*-3-(((3-(4-(*tert*-butoxycarbonyl)­piperazin-1-yl)­propyl)­(*(S)*-5,6,7,8-tetrahydroquino-lin-8-yl)­amino)­methyl)-5-morpholino-3,4-dihydroisoquinoline-2­(1*H*)-carboxylate

Step 1.

Procedure C was used, starting with **56** and **32**. It was used as is for the next step.

Step 2.

Procedure A was used, starting with *tert*-butyl *(R)*-3-(((3-(4-(*tert*-butoxycarbonyl)­piperazin-1-yl)­propyl)­(*(S)*-5,6,7,8-tetrahydroquinolin-8-yl)­amino)­methyl)-5-morpholino-3,4-dihydroisoquinoline-2­(1*H*)-carboxylate to afford a 53% yield of an off-white foam.

^1^H NMR (500 MHz, CDCl_3_): δ 8.41 (dd, *J =* 4.7, 1.8 Hz, 1H), 7.45–7.40 (m, 1H), 7.26–7.20 (m, 1H), 7.14 (dd, *J =* 7.7, 4.7 Hz, 1H), 6.98 (dd, *J =* 8.0, 1.3 Hz, 1H), 6.88 (dd, *J =* 7.6, 1.2 Hz, 1H), 4.59 (d, *J =* 16.2 Hz, 1H), 4.35–4.07 (m, 3H), 3.96–3.70 (m, 6H), 3.27 (s, 2H), 3.18 (d, *J =* 16.9 Hz, 1H), 3.01 (dd, *J =* 23.1, 16.0 Hz, 5H), 2.85–2.66 (m, 11H), 2.55 (s, 2H), 2.03 (d, *J =* 12.3 Hz, 1H), 1.81 (dt, *J =* 25.5, 13.3 Hz, 4H); LC-MS (ESI-API, 254 nm) 75–95% MeOH in H_2_O (0.1% HCO_2_H), 3 min, 1.00 mL/min, C18 (Agilent Zorbax XDB-18, 50 mm × 4.6 mm, 3.5 μm), *m*/*z* = 505.4 (M + 1), t = 0.597 min, purity: ≥ 95%; HRMS calculated for C_30_H_45_N_6_O (M+H): 505.35766, found: 505.36559

##### Benzyl-*(S)*-4-(3-((*tert*-butoxycarbonyl)­(5,6,7,8-tetrahydroquinolin-8-yl)­amino)­propyl)­pipe-razine-1-carboxylate (**59**)

To a solution of 5.15 g (12.606 mmol) of **57** and 5.5025 g (25.212 mmol) di-*tert*-butyl dicarbonate in 100 mL of 1,4-dioxane, 63 mL (60.33 mmol) of 1 M NaOH solution was added and stirred overnight. The reaction was diluted with EA and extracted with water. The organic layer was extracted with saturated NaHCO_3_ solution, separated, dried over anhydrous MgSO_4_, filtered, and evaporated. Product was purified with column chromatography, starting with DCM and increasing the polarity with DCM:MeOH:NH_3_ (7N in MeOH) (90:10:2), to yield 3.36 g (52% yield) of a yellow oil.

^1^H NMR (500 MHz, CDCl_3_): δ 8.40 (s, 1H), 7.32 (dd, *J =* 13.8, 6.0 Hz, 1H), 7.09–6.98 (m, 1H), 3.38–3.03 (m, 1H), 2.97–2.62 (m, 3H), 2.57–2.35 (m, 9H), 2.29–2.10 (m, 2H), 2.08–1.75 (m, 3H), 1.63 (s, 5H), 1.48 (s, 4H), 1.15 (s, 5H), 1.08 (t, *J =* 7.2 Hz, 3H).

***(S)*** **-N-(3-(4-Ethylpiperazin-1-yl)­propyl)-5,6,7,8-tetrahydroquinolin-8-amine (60)** was synthesized using the following steps.

Step 1.

##### *tert*-Butyl*(S)*-(3-(piperazin-1-yl)­propyl)­(5,6,7,8-tetrahydroquinolin-8-yl)­carbamate

3.36 g (6.61 mmol) of **59** was dissolved in ethanol (100 mL), and palladium hydroxide on carbon (0.927 g) and ammonium formate (1.67 g) were added to the reaction mixture. It was heated to reflux for 2 h. The reaction mixture was cooled to room temperature and filtered over Celite. The filtrate was evaporated and used as is for the next step.

Step 2.

##### *tert*-Butyl*(S)*-(3-(4-ethylpiperazin-1-yl)­propyl)­(5,6,7,8-tetrahydroquinolin-8-yl)­carbamate

Procedure C was used, starting with *tert*-butyl-*(S)*-(3-(piperazin-1-yl)­propyl)­(5,6,7,8-tetrahydroquinolin-8-yl)­carbamate and acetaldehyde. The crude material was purified with column chromatography, starting with DCM and increasing the polarity with DCM:MeOH:NH_3_ (7N in MeOH) (90:10:2), to give 2.45 g (93% yield over 2 steps) of a brownish oil.

^1^H NMR (500 MHz, CDCl_3_): δ 8.40 (s, 1H), 7.32 (dd, *J =* 13.8, 6.0 Hz, 1H), 7.09–6.98 (m, 1H), 3.38–3.03 (m, 1H), 2.97–2.62 (m, 3H), 2.57–2.35 (m, 9H), 2.29–2.10 (m, 2H), 2.08–1.75 (m, 3H), 1.63 (s, 5H), 1.48 (s, 4H), 1.15 (s, 5H), 1.08 (t, *J =* 7.2 Hz, 3H).

Step 3.

##### *(S)*-N-(3-(4-Ethylpiperazin-1-yl)­propyl)-5,6,7,8-tetrahydroquinolin-8-amine (**60**)

Procedure A was used, starting with *tert*-butyl *(S)*-(3-(4-ethylpiperazin-1-yl)­propyl)­(5,6,7,8-tetrahydroquinolin-8-yl)­carbamate and it was used as is for the next step.

***(S)*** **-N-(3-(4-Ethylpiperazin-1-yl)­propyl)-N-((** ***(R)*** **-5-morpholino-1,2,3,4-tetrahydroisoquinolin-3-yl)­methyl)-5,6,7,8-tetrahydroquinolin-8-amine (61)** was synthesized through the following steps.

Step 1.

##### *tert*-Butyl*(R)*-3-(((3-(4-ethylpiperazin-1-yl)­propyl)­(*(S)*-5,6,7,8-tetrahydroquinolin-8-yl)­amino)-methyl)-5-morpholino-3,4-dihydroisoquinoline-2­(1*H*)-carboxylate

Procedure C was used, starting with **60** and **32,** and the addition of titanium­(IV) isopropoxide was used as is, without further purification, for the next step.

Step 2.

Procedure A was used, starting with *tert*-butyl *(R)*-3-(((3-(4-ethylpiperazin-1-yl)­propyl)­(*(S)*-5,6,7,8-tetrahydroquinolin-8-yl)­amino)­methyl)-5-morpholino-3,4-dihydroisoquinoline-2­(1*H*)-carboxylate to afford a 76.4% yield of an off-white foam.

^1^H NMR (500 MHz, CDCl_3_): δ 8.41 (d, *J =* 4.7 Hz, 1H), 7.35 (d, *J =* 7.7 Hz, 1H), 7.12 (t, *J =* 7.8 Hz, 1H), 7.06 (dd, *J =* 7.7, 4.7 Hz, 1H), 6.89 (d, *J =* 7.9 Hz, 1H), 6.80 (d, *J =* 7.6 Hz, 1H), 4.16 (s, 4H), 4.01 (d, *J =* 15.0 Hz, 1H), 3.88–3.67 (m, 6H), 3.17–2.95 (m, 6H), 2.94–2.57 (m, 6H), 2.55–2.19 (m, 7H), 2.09 (d, *J =* 12.5 Hz, 1H), 1.93 (ddd, *J =* 35.5, 14.2, 9.9 Hz, 3H), 1.81–1.61 (m, 5H), 1.09–1.06 (m, 3H); ^13^C NMR (126 MHz, CDCl_3_): δ 158.75, 151.06, 146.50, 136.80, 135.08, 134.07, 129.48, 126.38, 121.94, 121.53, 117.02, 67.40, 61.82, 57.22, 56.47, 53.24, 52.72, 52.26, 52.17, 51.38, 47.65, 29.32, 29.07, 26.86, 21.93, 11.94; LC-MS (ESI-API, 254 nm) 75–95% MeOH in H_2_O (0.1% HCO_2_H), 3 min, 1.00 mL/min, C18 (Agilent Zorbax XDB-18, 50 mm × 4.6 mm, 3.5 μm), *m*/*z* = 533.4 (M + 1), t = 0.421 min, purity: **≥** 95%; HRMS calculated for C_32_H_49_N_6_O 533.39624; found 533.39615 [M + H].

##### *(R)*-5-(*(R)*-3-Methylmorpholino)-3,3a,4,9-tetrahydro-2H-isoxazolo­[2,3-*b*]­isoquinolin-2-one (**63**)

Procedure B was used, starting with **27** and **62i**. It was purified with column chromatography, starting with DCM and increasing the polarity with DCM:MeOH:NH_3_ (7N in MeOH) (90:10:2), to afford a 40% yield of compound **63**.

^1^H NMR (600 MHz, CDCl_3_): δ 7.51–7.44 (m, 1H), 7.14–7.11 (m, 1H), 6.97 (dd, *J =* 7.6, 1.2 Hz, 1H), 4.83–4.79 (m, 1H), 4.59 (td, *J =* 8.6, 7.9 Hz, 2H), 4.35–4.32 (m, 1H), 4.20–4.18 (m, 1H), 4.00–3.89 (m, 1H), 3.86–3.83 (m, 1H), 3.70 (td, *J =* 11.1, 2.5 Hz, 1H), 3.39 (dd, *J =* 16.2, 4.0 Hz, 1H), 3.22 (dd, *J =* 16.3, 4.3 Hz, 1H), 3.08 (ddp, *J =* 9.7, 6.5, 3.1 Hz, 1H), 2.92–2.87 (m, 1H), 2.68–2.61 (m, 1H), 2.51 (dd, *J =* 16.4, 11.1 Hz, 1H), 0.64 (d, *J =* 6.2 Hz, 3H).

##### *(R)*-5-(*(S)*-3-Methylmorpholino)-3,3a,4,9-tetrahydro-2H-isoxazolo­[2,3-*b*]­isoquinolin-2-one (**64**)

Procedure B was used, starting with **27** and **62j**. It was purified with column chromatography, starting with DCM and increasing the polarity with DCM:MeOH:NH_3_ (7N in MeOH) (90:10:2), to afford a 57% yield of compound **64**.

^1^H NMR (600 MHz, CDCl_3_): δ 7.51–7.44 (m, 1H), 7.14–7.11 (m, 1H), 6.97 (dd, *J =* 7.6, 1.2 Hz, 1H), 4.83–4.79 (m, 1H), 4.59 (td, *J =* 8.6, 7.9 Hz, 2H), 4.35–4.32 (m, 1H), 4.20–4.18 (m, 1H), 4.00–3.89 (m, 1H), 3.86–3.83 (m, 1H), 3.70 (td, *J =* 11.1, 2.5 Hz, 1H), 3.39 (dd, *J =* 16.2, 4.0 Hz, 1H), 3.22 (dd, *J =* 16.3, 4.3 Hz, 1H), 3.08 (ddp, *J =* 9.7, 6.5, 3.1 Hz, 1H), 2.92–2.87 (m, 1H), 2.68–2.61 (m, 1H), 2.51 (dd, *J =* 16.4, 11.1 Hz, 1H), 0.64 (d, *J =* 6.2 Hz, 3H).

***tert*-Butyl*(R)*-3-formyl-5-(*(R)*-3-methylmorpholino)-3,4-dihydroisoquinoline-2­(1*H*)-carboxylate (65)** was synthesized with the following steps.

Step 1.

##### (*(R)*-5-(*(R)*-3-Methylmorpholino)-1,2,3,4-tetrahydroisoquinolin-3-yl)­methanol

Compound **63** (0.485 g, 1.68 mmol) was dissolved in MeOH (10 mL) and 6N NaOH (1.24 mL, 7.46 mmol) was added. The reaction mixture was heated to 100 °C for 3 h and then cooled to room temperature; there were some precipitations. The solvent was evaporated. The residue was suspended in 5 mL of water and stirred overnight to allow the precipitation to complete. The precipitates were filtered and used for the next step without any purification.

Step 2.

##### *tert*-Butyl*(R)*-3-(hydroxymethyl)-5-(*(R)*-3-methylmorpholino)-3,4-dihydroisoquinoline-2­(1*H*)-carboxylate

[(3*R*)-5-[(3*R*)-3-methylmorpholin-4-yl]-1,2,3,4-tetrahydroisoquinolin-3-yl]­methanol (0.44 g, 1.68 mmol) was dissolved in THF (6 mL), and Na_2_CO_3_ (0.533 g, 5.03 mmol) was added with a few drops of water. The reaction mixture was stirred . Di-*tert*-butyl dicarbonate (0.58 mL, 2.52 mmol) was weighed and added to the reaction mixture, and continued to stir at room temperature overnight. The reaction was diluted with DCM and extracted with water. rThe oganic phase was dried with anhydrous MgSO_4_, filtered off, and evaporated. The residue was purified using column chromatography, starting with hexanes and gradually increasing the polarity with EA to give *tert*-butyl (3*R*)-3-(hydroxymethyl)-5-[(3*R*)-3-methylmorpholin-4-yl]-3,4-dihydro-1*H*-isoquinoline-2-carboxylate (220 mg, 0.60 mmol, 36% yield).

^1^H NMR (600 MHz, CDCl_3_): δ 7.19 (td, *J =* 7.7, 4.3 Hz, 1H), 7.05 (d, *J =* 7.6 Hz, 1H), 6.93 (d, *J =* 6.8 Hz, 1H), 4.70–4.45 (m, 2H), 4.35 (dd, *J =* 16.3, 4.2 Hz, 1H), 3.86 (ddt, *J =* 18.9, 8.0, 3.1 Hz, 2H), 3.78 (td, *J =* 11.3, 9.9, 5.2 Hz, 1H), 3.52–3.10 (m, 6H), 2.88–2.53 (m, 3H), 1.51 (d, *J =* 4.8 Hz, 9H), 0.75 (d, *J =* 6.2 Hz, 3H).

Step 3.

##### *tert*-Butyl*(R)*-3-formyl-5-(*(R)*-3-methylmorpholino)-3,4-dihydroisoquinoline-2­(1*H*)-carboxylate (**65**)

To the solution of *tert*-butyl (3*R*)-3-(hydroxymethyl)-5-[(3*R*)-3-methylmorpholin-4-yl]-3,4-dihydro-1*H*-isoquinoline-2-carboxylate (0.22 g, 0.6 mmol) in DCM (1.7 mL), TEA (0.42 mL, 2.99 mmol) was added, and the mixture was cooled to 0 °C with an ice bath. The solution of pyridine sulfur trioxide (0.38 g, 2.39 mmol) in DMSO (1.7 mL) was added to the reaction mixture at 0 °C. The reaction was stirred at 0 °C for 4 h. Sodium bicarbonate solution was added to the reaction mixture, which was then diluted with EA and stirred for 30 min. The phases were separated, and the aqueous phase was extracted with EA, and combined organic layer was dried over anhydrous MgSO_4_, filtered off, and evaporated. It was used as is for the next step.

***tert*-Butyl*(R)*-3-formyl-5-(*(S)*-3-methylmorpholino)-3,4-dihydroisoquinoline-2­(1*H*)-carboxylate (66)** was synthesized using the following steps.

Step 1.

##### (*(R)*-5-(*(S)*-3-Methylmorpholino)-1,2,3,4-tetrahydroisoquinolin-3-yl)­methanol

Compound **64** was suspended in MeOH (14 mL), and NaOH (3.57 mL, 21.4 mmol) was added. The mixture was heated to 100 °C for 2 h. The reaction mixture was then cooled to room temperature, resulting in some precipitations. The solvent was evaporated. The residue was suspended in 5 mL of water and stirred overnight for the precipitation to complete. The precipitates were filtered and used for the next step without any purification.

Step 2.

##### *tert*-Butyl*(R)*-3-(hydroxymethyl)-5-(*(S)*-3-methylmorpholino)-3,4-dihydroisoquinoline-2­(1*H*)-carboxylate

[(3*R*)-5-[(3*S*)-3-methylmorpholin-4-yl]-1,2,3,4-tetrahydroisoquinolin-3-yl]­methanol (0.56 g, 2.14 mmol) was dissolved in THF (10 mL), and sodium carbonate (907 mg, 8.56 mmol) was added with a few drops of water and stirred for a few minutes. Then, di-*tert*-butyl dicarbonate (0.89 mL, 3.85 mmol) was added to the reaction mixture and stirring was continued at room temperature overnight. The reaction was diluted with DCM and extracted with water. The organic phase was dried over anhydrous MgSO_4_, filtered off, and the filtrate was evaporated to dryness. The residue was purified with column chromatography with hexanes:EA to get *tert*-butyl (3*R*)-3-(hydroxymethyl)-5-[(3*S*)-3-methylmorpholin-4-yl]-3,4-dihydro-1*H*-isoquinoline-2-carboxylate (443 mg, 1.22 mmol, 57% yield).

^1^H NMR (400 MHz, CDCl_3_): δ 7.22 (t, *J =* 7.7 Hz, 1H), 7.14 (dd, *J =* 8.1, 1.3 Hz, 1H), 6.97 (d, *J =* 7.4 Hz, 1H), 4.68 (s, 1H), 4.43–4.33 (m, 1H), 4.29 (d, *J =* 16.0 Hz, 1H), 3.88 (ddd, *J =* 11.1, 3.2, 1.2 Hz, 2H), 3.78 (td, *J =* 10.7, 2.6 Hz, 1H), 3.56 (s, 2H), 3.38 (dd, *J =* 11.0, 9.2 Hz, 1H), 3.18 (ddp, *J =* 9.4, 6.2, 3.1 Hz, 1H), 3.00 (d, *J =* 5.6 Hz, 1H), 2.87 (ddd, *J =* 11.9, 10.3, 3.2 Hz, 1H), 2.75 (d, *J =* 12.0 Hz, 1H), 1.62 (s, 1H), 1.53 (s, 9H), 0.75 (d, *J =* 6.3 Hz, 3H).

Step 3.

##### *tert*-Butyl*(R)*-3-formyl-5-(*(S)*-3-methylmorpholino)-3,4-dihydroisoquinoline-2­(1*H*)-carboxylate (**66**)

To the solution of *tert*-butyl (3*R*)-3-(hydroxymethyl)-5-[(3*S*)-3-methylmorpholin-4-yl]-3,4-dihydro-1*H*-isoquinoline-2-carboxylate (0.23 g, 0.62 mmol) in DCM (2 mL), TEA (0.43 mL, 3.1 mmol) was added, and the mixture was cooled to 0 °C with an ice bath. The solution of pyridine sulfur trioxide (0.40 g, 2.48 mmol) in DMSO (2 mL) was added to the reaction mixture at 0 °C. The reaction was stirred at 0 °C for 4 h. Sodium bicarbonate solution was added to the reaction mixture, diluted with EA, and stirred for 30 min. The phases were separated, and the aqueous phase was extracted with EA. The combined organic layer was dried over anhydrous MgSO_4_, filtered off, and evaporated. It was used it as is for the next step.

***tert*** **-Butyl*(R)*-5-bromo-3-formyl-3,4-dihydroisoquinoline-2­(1*H*)-carboxylate (68)** was synthesized by the following steps.

Step 1.

##### *(R)*-(5-Bromo-1,2,3,4-tetrahydroisoquinolin-3-yl)­methanol

A 500 mL round-bottom flask equipped with a reflux condenser and a magnetic stir bar was charged with 11.0 g of **27** (41.2 mmol, 1 equiv) and 100 mL of MeOH. Then, 68.6 mL of 6N NaOH solution (412 mmol, 10 equiv) was added, and the reaction mixture was refluxed. After heating for 3 h, the reaction mixture was allowed to cool to room temperature, and the product was crystallized. 200 mL of water was added, and the suspension was stirred for 30 min, then cooled in an ice bath. The solids were filtered, washed with water, and dried, affording 9.25 g (92% yield) of the product as a white solid. It is used as is for the next step.

Step 2.

##### *tert*-Butyl*(R)*-5-bromo-3-(hydroxymethyl)-3,4-dihydroisoquinoline-2­(1*H*)-carboxylate

To the suspension of [(3*R*)-5-bromo-1,2,3,4-tetrahydroisoquinolin-3-yl] MeOH (18.49 g, 76.37 mmol) in 200 mL THF, a sodium carbonate (8.09 g, 76.37 mmol) solution in 100 mL water was added, followed by di-*tert*-butyl dicarbonate (17.73 mL, 76.37 mmol), and it wasstirred overnight. The reaction mixture was quenched by the addition of 2 N HCl solution until pH was 3, extracted with diethyl ether, washed with water and brine, and dried over anhydrous Na_2_SO_4_. The organics were concentrated, and the crude product was purified by crystallization from heptane to give 23.37 g (90% yield) of off-white crystals.

^1^H NMR (400 MHz, CDCl_3_): δ 7.46–7.41 (m, 1H), 7.08–7.01 (m, 2H), 4.92–4.43 (m, 1H), 4.27 (d, *J =* 16.8 Hz, 1H), 3.51 (s, 3H), 2.98 (d, *J =* 15.4 Hz, 1H), 2.92 (dd, *J =* 16.8, 6.0 Hz, 2H), 1.48 (s, 9H).

Step 3.

##### *tert*-Butyl*(R)*-5-bromo-3-formyl-3,4-dihydroisoquinoline-2­(1H)-carboxylate (**68**)

A 500 mL round-bottom flask equipped with a magnetic stir bar and septum was charged with 15.1 g *tert*-butyl *(R)*-5-bromo-3-(hydroxymethyl)-3,4-dihydroisoquinoline-2­(1H)-carboxylate (44.1 mmol, 1 equiv), 32.6 mL of TEA (234 mmol, 5.3 equiv), and 132 mL of DCM. After the reaction mixture was cooled to 0 °C, 28.1 g of SO3*Py (176 mmol, 4 equiv) dissolved in 46 mL of DMSO was added dropwise, and the reaction mixture was stirred at 0 °C for 2 h. Then the reaction mixture was quenched by the addition of sat. NaHCO_3_ solution, extracted with DCM, washed with water, and dried over anhydrous Na_2_SO_4_. After the organics were concentrated, the aldehyde was used in the next step without further purification.

##### *tert*-Butyl*(R)*-5-bromo-3-((methyl­(*(S)*-5,6,7,8-tetrahydroquinolin-8-yl)­amino)­methyl)-3,4-dihydroisoquinoline-2­(1*H*)-carboxylate (**69**)

To the solution of compound **67** (5.7 g, 35.14 mmol) in DCE (100 mL), STAB (19.19 g, 87.84 mmol) was added and stirred for 30 min at room temperature. Then, **68** (11.95 g, 35.14 mmol) was added to the reaction mixture and stirring was continued at room temperature overnight. The reaction was quenched with a saturated NaHCO_3_ solution. The aqueous phase was extracted with DCM. The combined organic layer was dried over anhydrous MgSO_4_, filtered off, and evaporated. The crude product was purified with column chromatography, starting with hexanes and increasing the polarity with EA slowly to 50% EA in hexanes. 13.5 g (79% yield) of an off-white foam was obtained.

^1^H NMR (500 MHz, CDCl_3_): δ 8.32 (d, *J =* 4.5 Hz, 1H), 7.37 (d, *J =* 7.8 Hz, 1H), 7.31–7.27 (m, 1H), 6.96 (t, *J =* 7.6 Hz, 2H), 6.91–6.83 (m, 1H), 4.65 (d, *J =* 18.6 Hz, 2H), 3.80 (d, *J =* 18.5 Hz, 1H), 3.71 (dd, *J =* 8.1, 4.7 Hz, 1H), 3.16 (d, *J =* 16.9 Hz, 1H), 2.84–2.71 (m, 2H), 2.67–2.57 (m, 2H), 2.38 (s, 3H), 2.02–1.91 (m, 2H), 1.88–1.74 (m, 2H), 1.68–1.58 (m, 1H), 1.49 (s, 9H); ^**13**^C NMR (151 MHz, CDCl_3_): δ 171.13, 157.70, 154.92, 146.63, 136.48, 135.34, 133.85, 130.34, 126.92, 125.29, 121.46, 79.75, 77.23, 64.75, 60.39, 48.15, 42.09, 28.62, 28.50, 26.40, 21.05, 20.14, 14.20; HRMS calculated for C_25_H_32_O_2_N_3_Br: 486.17507; found 486.17548 [M + H].

##### *tert*-Butyl*(R)*-3-((methyl­(*(S)*-5,6,7,8-tetrahydroquinolin-8-yl)­amino)­methyl)-5-(*(R)*-2-methylmorpholino)-3,4-dihydroisoquinoline-2­(1*H*)-carboxylate (**70a**)

Procedure B was used, starting with **69** and **62a**. The crude product was purified by column chromatography using EA:hexanes to afford a 93% yield of **70a**.

^1^H NMR (600 MHz, CDCl_3_): δ 8.26 (s, 1H), 7.32 (d, *J =* 7.7 Hz, 1H), 7.09 (t, *J =* 7.7 Hz, 1H), 6.99 (s, 1H), 6.84 (d, *J =* 7.9 Hz, 1H), 6.71 (d, *J =* 7.6 Hz, 0.6H), 6.67 (s, 0.4H), 4.63 (s, 0.4H), 4.57 (d, *J =* 17.0 Hz, 1H), 4.42 (s, 0.6H), 3.94–3.86 (m, 3H), 3.81–3.76 (m, 1H), 3.56 (s, 1H), 3.29–3.20 (m, 1H), 2.93 (d, *J =* 12.2 Hz, 1H), 2.88 (d, *J =* 11.2 Hz, 1H), 2.85–2.78 (m, 1H), 2.72 (t, *J =* 10.4 Hz, 1H), 2.68–2.60 (m, 3H), 2.56 (s, 1H), 2.45 (s, 0.4H), 2.35 (t, *J =* 10.8 Hz, 0.6H), 2.27 (s, 3H), 1.99 (s, 1H), 1.95–1.84 (m, 2H), 1.61–1.52 (m, 1H), 1.50 (s, 9H), 1.24 (d, *J =* 6.3 Hz, 3H); LC-MS (ESI-API, 254 nm) 50–95% MeOH in H_2_O (0.1% HCO_2_H), 6 min, 0.5 mL/min, C18 (Agilent Zorbax XDB-C18, 50 mm × 2.1 mm, 3.5 μm), *m*/*z* = 507.6 (M + H), t = 3.160 min.

##### *tert*-Butyl*(R)*-3-((methyl­(*(S)*-5,6,7,8-tetrahydroquinolin-8-yl)­amino)­methyl)-5-(*(S)*-2-methylmorpholino)-3,4-dihydroisoquinoline-2­(1*H*)-carboxylate (**70b**)

Procedure B was used, starting with **69** and **62b**. The crude product was purified by column chromatography using EA:hexanes to afford a 78% yield of **70b**.

^1^H NMR (400 MHz, CDCl_3_): δ 8.28 (s, 1H), 7.35 (d, *J =* 7.7 Hz, 1H), 7.12 (t, *J =* 7.8 Hz, 1H), 7.05–6.97 (m, 1H), 6.86 (dd, *J =* 8.0, 1.2 Hz, 1H), 6.72 (t, *J =* 9.7 Hz, 1H), 4.60 (d, *J =* 17.0 Hz, 1H), 4.02 (ddd, *J =* 11.1, 3.1, 1.6 Hz, 1H), 3.96–3.75 (m, 3H), 3.60 (s, 1H), 3.26 (t, *J =* 14.9 Hz, 1H), 3.14–2.94 (m, 2H), 2.83 (dd, *J =* 16.5, 9.0 Hz, 2H), 2.76–2.57 (m, 3H), 2.28 (s, 3H), 2.05–1.84 (m, 3H), 1.71–1.62 (m, 7H), 1.52 (s, 9H), 1.19 (d, *J =* 6.3 Hz, 3H).

##### *tert*-Butyl*(R)*-5-(*(S)*-2-(hydroxymethyl)­morpholino)-3-((methyl­(*(S)*-5,6,7,8-tetrahydroquinolin-8-yl)­amino)­methyl)-3,4-dihydroisoquinoline-2­(1*H*)-carboxylate (**70c**)

Procedure B was used, starting with **69** and **62c**. The crude product was purified by column chromatography, starting with DCM and increasing the polarity with DCM:MeOH:NH_3_ (7N in MeOH) (90:10:2), to afford a 55% yield of **70c**.

^1^H NMR (400 MHz, CDCl_3_): δ 8.30 (s, 1H), 7.35 (d, *J =* 7.7 Hz, 1H), 7.12 (t, *J =* 7.8 Hz, 1H), 7.03 (s, 1H), 6.86 (dd, *J =* 8.0, 1.1 Hz, 1H), 6.73 (q, *J =* 7.6 Hz, 1H), 4.61 (d, *J =* 17.6 Hz, 2H), 4.05–3.54 (m, 5H), 3.22 (d, *J =* 16.1 Hz, 1H), 3.04–2.56 (m, 6H), 2.31 (s, 4H), 2.12–1.79 (m, 1H), 1.67 (d, *J =* 25.3 Hz, 4H), 1.55 (s, 9H), 15.2–1.42 (m, 4H).

##### *tert*-Butyl*(R)*-5-(*(R)*-2-(hydroxymethyl)­morpholino)-3-((methyl­(*(S)*-5,6,7,8-tetrahydroquinolin-8-yl)­amino)­methyl)-3,4-dihydroisoquinoline-2­(1*H*)-carboxylate (**70d**)

Procedure B was used, starting with **69** and **62d**. The crude product was purified by column chromatography, starting with DCM and increasing the polarity with DCM:MeOH:NH_3_ (7N in MeOH) (90:10:2), to afford a 35% yield of **70d**.

^1^H NMR (400 MHz, CDCl_3_): δ 8.26 (s, 1H), 7.33 (d, *J =* 7.7 Hz, 1H), 7.09 (t, *J =* 7.8 Hz, 1H), 7.00 (t, *J =* 6.2 Hz, 1H), 6.86 (d, *J =* 7.9 Hz, 1H), 6.74–6.65 (m, 1H), 4.61 (br s, 0.4H), 4.58 (d, *J =* 16.9 Hz, 1H), 4.41 (s, 0.6H), 4.06 (dt, *J =* 11.0, 2.7 Hz, 1H), 3.93–3.81 (m, 3H), 3.77–3.69 (m, 1H), 3.65–3.56 (m, 2H), 3.21 (d, *J =* 15.9 Hz, 1H), 3.05 (td, *J =* 11.0, 3.1 Hz, 1H), 2.99 (d, *J =* 11.9 Hz, 1H), 2.92–2.83 (m, 1H), 2.81 (q, *J =* 8.2, 7.5 Hz, 1H), 2.76–2.59 (m, 2H), 2.49 (t, *J =* 10.7 Hz, 1H), 2.33 (s, 0.4H), 2.27 (s, 3H), 2.18 (s, 0.6H), 2.03–1.93 (m, 1H), 1.93–1.83 (m, 2H), 1.66–1.55 (m, 2H), 1.50 (s, 9H); LC-MS (ESI-API, 254 nm) 50–95% MeOH in H_2_O (0.1% HCO_2_H), 6 min, 0.5 mL/min, C18 (Agilent Zorbax XDB-C18, 50 mm × 2.1 mm, 3.5 μm), *m*/*z* = 523.6 (M + H), 234.3 (M/2 + H), t = 1.349 min.

##### *tert*-Butyl*(R)*-5-((1*R*,4*R*)-2-Oxa-5-azabicyclo­[2.2.1]­heptan-5-yl)-3-((methyl­(*(S)*-5,6,7,8-tetrahydroquinolin-8-yl)­amino)­methyl)-3,4-dihydroisoquinoline-2­(1*H*)-carboxylate (**70e**)

Procedure B was used, starting with **69** and **62e**. The crude product was purified by column chromatography using EA:hexanes to afford a 59% yield of **70e**.

^1^H NMR (400 MHz, CDCl_3_): δ 8.24 (s, 1H), 7.24 (d, 1H, J = 7.2 Hz), 6.93 (t, 2H, J = 7.6 Hz), 6.47 (d, 1H, J = 7.2 Hz), 6.44 (d, 1H, J = 8 Hz), 4.53 (m, 2H), 4.22 (d, 1H, J = 7.2 Hz), 4.08 (m, 1H), 3.84 (dd, 2H, J = 1.6, 7.6 Hz), 3.77 (d, 1H, J = 8.8 Hz), 3.60 (br s, 1H), 2.90 (m, 1H), 2.73 (m, 2H), 2.57 (dt, 1H, J = 5.2, 16.8 Hz), 2.42 (m, 1H), 2.29 (s, 3H), 2.20 (m, 1H), 1.89 (m, 2H), 1.81 (m, 2H), 1.56 (m, 1H), 1.47 (s, 9H); MS: 505.32 *m*/*z* (M+H^+^).

##### *tert*-Butyl*(R)*-5-((1*S*,4*S*)-2-oxa-5-azabicyclo­[2.2.1]­heptan-5-yl)-3-((methyl­(*(S)*-5,6,7,8-tetrahydroquinolin-8-yl)­amino)­methyl)-3,4-dihydroisoquinoline-2­(1*H*)-carboxylate (**70f**)

Procedure B was used, starting with **69** and **62f**. The crude product was purified by column chromatography using EA:hexanes to afford a 60% yield of **70f**.

^1^H NMR (400 MHz, CDCl_3_): δ 8.23 (d, 1H, J = 3.2 Hz), 7.29 (d, 1H, J = 7.2 Hz), 6.99 (quart, 1H, J = 8 Hz), 6.97 (m, 1H), 6.82 (d, 1H, J = 8 Hz), 6.53 (br s, 1H), 4.56 (s, 2H), 3.88 (d, 1H, J = 7.6 Hz), 3.80 (d, 1H, J = 17.2 Hz), 3.74 (dd, 1H, J = 2, 7.2 Hz), 3.55 (d, 1H, J = 9.6 Hz), 3.05 (d, 1H, J = 16 Hz), 2.85 (m, 1H), 2.77 (quart, 1H, J = 8.4 Hz), 2.59 (m, 3H), 2.27 (s, 3H), 1.96 (m, 1H), 1.88 (ABX, 2H, J = 9.6, 18 Hz), 1.84 (m, 1H), 1.56 (m, 1H), 1.47 (s, 9H); MS: 505.32 *m*/*z* (M+H^+^).

##### *tert*-Butyl*(R)*-5-((2*S*,6*R*)-2,6-dimethylmorpholino)-3-((methyl­(*(S)*-5,6,7,8-tetrahydroquinolin-8-yl)­amino)­methyl)-3,4-dihydroisoquinoline-2­(1*H*)-carboxylate (**70g**)

Procedure B was used, starting with **69** and **62g**. The crude product was purified by column chromatography using EA:hexanes to afford a 24% yield of **70g**.

^1^H NMR (400 MHz, CDCl_3_): δ 8.32 (d, *J =* 58.7 Hz, 1H), 7.46 (d, *J =* 7.5 Hz, 1H), 7.40–7.23 (m, 1H), 7.21–7.13 (m, 1H), 7.11–6.58 (m, 2H), 5.14 (d, *J =* 35.0 Hz, 2H), 4.87–4.33 (m, 2H), 4.25–3.75 (m, 2H), 3.75–3.11 (m, 4H), 3.08–2.78 (m, 4H), 2.64 (t, *J =* 9.6 Hz, 2H), 2.26 (s, 3H), 2.24–1.69 (m, 4H), 1.49 (s, 9H), 1.20 (d, *J =* 28.0 Hz, 6H); HRMS calculated for C_31_H_45_N_4_O_3_ 521.34705; found 521.34746 [M + H].

##### *tert*-Butyl*(R)*-5-((1*R*,5*S*)-8-Oxa-3-azabicyclo­[3.2.1]­octan-3-yl)-3-((methyl­(*(S)*-5,6,7,8-tetrahydroquinolin-8-yl)­amino)­methyl)-3,4-dihydroisoquinoline-2­(1*H*)-carboxylate (**70h**)

Procedure B was used, starting with **69** and **62h**. The crude product was purified with column chromatography using EA:hexanes to afford a 40% yield of a white foam.

^1^H NMR (400 MHz, CDCl_3_): δ 8.22 (d, 1H, J = 4 Hz), 7.33 (d, 1H, J = 8 Hz), 7.07 (t, 1H, J = 8 Hz), 7.00 (dd, 1H, J = 5, 7 Hz), 6.86 (d, 1H, J = 8 Hz), 6.65 (d, 1H, J = 6 Hz), 4.57 (ABq, 2H, J = 32 Hz, z = 49 Hz), 4.41 (d, 1H, J = 6 Hz), 4.32 (d, 1H, J = 6 Hz), 3.74 (t, 1H, J = 17 Hz), 3.60 (t, 1H, J = 6 Hz), 3.24 (d, 1H, J = 12 Hz), 3.14 (d, 1H, J = 17 Hz), 2.82 (m, 2H), 2.69 (m, 6H), 2.61 (t, 1H, J = 6 Hz), 2.25 (s, 3H), 2.21 (m, 1H), 2.09 (m, 1H), 1.93 (m, 2H), 1.79 (m, 1H), 1.72 (m, 1H), 1.59 (m, 1H), 1.48 (s, 9H); HRMS calculated for C_31_H_43_O_3_N_4_ 519.33297; found 519.33292 [M + H].

##### *tert*-Butyl*(R)*-3-((methyl­(*(S)*-5,6,7,8-tetrahydroquinolin-8-yl)­amino)­methyl)-5-(3-oxomorpholino)-3,4-dihydroisoquinoline-2­(1*H*)-carboxylate (**70i**)

A 20 mL μW tube equipped with a stir bar was charged with 300 mg of **69** (0.617 mmol, 1 equiv), 74.8 mg of morpholine-3-one (0.740 mmol, 1.2 equiv), 170 mg of K_2_CO_3_ (1.23 mmol, 2 equiv), and 11.8 mg of CuI (0.0617 mmol, 0.1 equiv), and the system was set under an Ar atmosphere. Then, 6.1 mL of toluene (degassed by bubbling through Ar for 1 h) and 13.0 μL of N, N’-dimethylethane-1,2-diamine (0.123 mmol, 0.2 equiv) were added. After stirring at 110 °C for 20 h, EA and water were added, and the product was extracted with EA (3X) and dried over Na_2_SO_4_. The organics were concentrated, and the crude product was purified with column chromatography using EA as an eluent, affording 160 mg (51%) of the title compound as a white foam (3:1 mixture of atropisomers).

^1^H NMR (400 MHz, CDCl_3_): δ 8.34 (dd, *J =* 4.8, 1.7 Hz, 0.25H), 8.29–8.21 (m, 0.75H), 7.38–7.16 (m, 2H), 7.10–6.93 (m, 3H), 4.85–4.42 (m, 2H), 4.42–4.27 (m, 2H), 4.17–3.99 (m, 3H), 3.93–3.52 (m, 3H), 2.97–2.57 (m, 5H), 2.46–2.19 (m, 1H), 2.28–2.19 (m, 3H), 2.03–1.57 (m, 4H), 1.51 (s, 2.25H), 1.50 (s, 6.75H); LC-MS (ESI-API, 254 nm), 75–95% MeOH in H_2_O (0.1% HCO_2_H), 3 min, 1.00 mL/min, C18 (Agilent Zorbax XDB-18, 50 mm × 4.6 mm, 3.5 μm), *m*/*z* = 507.2 (M + H), t = 0.475 min.

##### *tert*-Butyl*(R)*-3-((methyl­(*(S)*-5,6,7,8-tetrahydroquinolin-8-yl)­amino)­methyl)-5-(*(R)*-3-methylmorpholino)-3,4-dihydroisoquinoline-2­(1*H*)-carboxylate (**70j**)

Procedure C was used, starting with **67** and **65.** The crude material was purified with column chromatography, starting with hexanes and increasing the polarity with EA, to afford a 73% yield of compound **70j**.

^1^H NMR (400 MHz, CDCl_3_): δ 8.28 (d, *J =* 4.0 Hz, 1H), 7.33 (d, *J =* 7.2 Hz, 1H), 7.12 (t, *J =* 7.7 Hz, 1H), 7.01 (td, *J =* 7.6, 6.6, 4.5 Hz, 1H), 6.94 (d, *J =* 7.8 Hz, 1H), 6.76 (d, *J =* 9.4 Hz, 1H), 4.71–4.57 (m, 2H), 3.95–3.73 (m, 6H), 3.48–3.18 (m, 3H), 3.05–2.40 (m, 6H), 2.32 (s, 3H), 2.02–1.59 (m, 4H), 1.52 (s, 9H), 0.80 (d, *J =* 6.3 Hz, 3H).

##### *tert*-Butyl*(R)*-3-((methyl­(*(S)*-5,6,7,8-tetrahydroquinolin-8-yl)­amino)­methyl)-5-(*(S)*-3-methylmorpholino)-3,4-dihydroisoquinoline-2­(1*H*)-carboxylate (**70k**)

Procedure C was used, starting with **67** and **66.** The crude material was purified with column chromatography, starting with hexanes and increasing the polarity with EA to afford a 79% yield of compound **70k**.

^1^H NMR (400 MHz, CDCl_3_): δ 8.32–8.24 (m, 1H), 7.39–7.30 (m, 1H), 7.14 (t, *J =* 7.6 Hz, 1H), 7.07 (d, *J =* 7.8 Hz, 1H), 7.01 (dd, *J =* 7.7, 4.7 Hz, 1H), 6.81 (d, *J =* 7.4 Hz, 1H), 4.66 (s, 2H), 4.48 (s, 1H), 3.94–3.76 (m, 4H), 3.69 (s, 1H), 3.41–3.28 (m, 1H), 3.25–3.03 (m, 2H), 2.89 (ddd, *J =* 13.5, 10.5, 3.4 Hz, 1H), 2.80 (dd, *J =* 16.1, 6.3 Hz, 3H), 2.73–2.43 (m, 1H), 2.31 (s, 3H), 2.09–1.83 (m, 3H), 1.68–1.56 (m, 2H), 1.52 (s, 9H), 0.71 (d, *J =* 6.2 Hz, 3H).

##### *(S)*-*N*-Methyl-N-((*(R)*-5-(*(R)*-2-methylmorpholino)-1,2,3,4-tetrahydroisoquinolin-3-yl)­methyl)-5,6,7,8-tetrahydroquinolin-8-amine (**71**)

Procedure A was used, starting with **70a**. The crude product was purified with column chromatography, starting with DCM and increasing the polarity with DCM:MeOH:NH_3_ (7N in MeOH) (90:10:2), to afford a 36% yield of a yellow foam.

^1^H NMR (400 MHz, CDCl_3_): δ 8.48 (dd, *J =* 4.6, 1.4 Hz, 1H), 7.35 (d, *J =* 7.5 Hz, 1H), 7.09 (t, *J =* 7.9 Hz, 1H), 7.07 (dd, *J =* 7.8, 4.6 Hz, 1H), 6.86 (d, *J =* 7.8 Hz, 1H), 6.78 (d, *J =* 7.3 Hz, 1H), 4.05 (A of AB, *J*_*AB*_ = 15.4 Hz, 1H), 3.99–3.89 (m, 3H), 3.81–3.73 (m, 2H), 3.02 (td, *J =* 11.2, 3.1 Hz, 1H), 2.92 (dt, *J =* 11.9, 1.9 Hz, 1H), 2.89–2.65 (m, 5H), 2.55 (s, 3H), 2.54–2.44 (m, 2H), 2.23 (dd, *J =* 12.0, 9.8 Hz, 1H), 2.19–1.92 (m, 4H), 1.79–1.64 (m, 1H), 1.61 (br s, 1H), 1.14 (d, *J =* 6.3 Hz, 3H); ^**13**^C NMR (151 MHz, CDCl_3_): δ 158.18, 151.00, 146.85, 137.08, 136.63, 133.82, 130.11, 126.06, 122.03, 121.52, 116.76, 72.09, 67.27, 64.47, 59.95, 59.14, 51.65, 50.93, 48.84, 41.58, 30.29, 29.23, 26.29, 21.27, 18.86; LC-MS (ESI-API, 254 nm) 10–95% MeOH in H_2_O (0.1% HCO_2_H), 6 min, 0.6 mL/min, C18 (Agilent Zorbax XDB-C18, 50 mm × 2.1 mm, 3.5 μm), *m*/*z* = 407.5 (M + H), 204.4 (M/2 + H), t = 3.572 min; HRMS calculated for C_25_H_35_ON_4_ 407.28054; found: 407.28018 [M + H].

##### *(S)*-*N*-Methyl-N-((*(R)*-5-(*(S)*-2-methylmorpholino)-1,2,3,4-tetrahydroisoquinolin-3-yl)­methyl)-5,6,7,8-tetrahydroquinolin-8-amine (**72**)

Procedure A was used, starting with **70b**. The crude product was purified with column chromatography, starting with DCM and increasing the polarity with DCM:MeOH:NH_3_ (7N in MeOH) (90:10:2), to afford a 32% yield of a yellow foam.

^1^H NMR (600 MHz, CDCl_3_): δ 8.44 (dd, *J =* 4.7, 1.7 Hz, 1H), 7.32 (ddd, *J =* 7.6, 1.9, 0.9 Hz, 1H), 7.13–6.99 (m, 2H), 6.82 (d, *J =* 7.8 Hz, 1H), 6.75 (d, *J =* 7.6 Hz, 1H), 4.04 (d, *J =* 15.4 Hz, 1H), 3.94–3.90 (m, 2H), 3.85 (ddd, *J =* 11.0, 3.1, 1.5 Hz, 1H), 3.78 (td, *J =* 11.1, 2.4 Hz, 1H), 3.72 (dtt, *J =* 12.5, 6.4, 3.1 Hz, 1H), 2.87–2.73 (m, 7H), 2.70–2.64 (m, 2H), 2.51 (ddd, *J =* 21.5, 10.6, 2.9 Hz, 3H), 2.47 (s, 3H), 2.16–2.08 (m, 1H), 2.08–1.88 (m, 2H), 1.68 (dtdd, *J =* 13.1, 10.5, 5.0, 2.6 Hz, 1H), 1.18 (d, *J =* 6.2 Hz, 3H); ^**13**^C NMR (151 MHz, CDCl_3_): δ 158.06, 150.96, 146.85, 136.71, 133.89, 130.01, 126.12, 122.00, 121.58, 116.82, 72.34, 67.27, 64.53, 59.99, 58.00, 52.13, 51.61, 48.65, 41.14, 30.21, 29.25, 26.05, 21.31, 19.05; HRMS calculated for C_25_H_35_N_4_O 407.28054; found 407.28024 [M + H].

##### *(S)*-N-((*(R)*-5-((2*S*,6*R*)-2,6-Dimethylmorpholino)-1,2,3,4-tetrahydroisoquinolin-3-yl)­methyl)-*N*-methyl-5,6,7,8-tetrahydroquinolin-8-amine (**73**)

Procedure A was used, starting with **70c** to afford an 81% yield of a white foam.

^1^H NMR (400 MHz, CDCl_3_): δ 8.43 (dd, *J =* 4.7, 1.9 Hz, 1H), 7.32 (dd, *J =* 7.7, 1.7 Hz, 1H), 7.12–7.01 (m, 2H), 6.81 (d, *J =* 7.6 Hz, 1H), 6.74 (d, *J =* 7.6 Hz, 1H), 4.07 (d, *J =* 15.5 Hz, 1H), 4.00–3.86 (m, 2H), 3.77 (ddtd, *J =* 16.0, 12.4, 6.2, 2.2 Hz, 2H), 2.87–2.76 (m, 6H), 2.71–2.47 (m, 3H), 2.42 (s, 3H), 2.35–2.08 (m, 3H), 2.04–1.91 (m, 2H), 1.76–1.56 (m, 1H), 1.23–1.19 (m, 1H), 1.17 (d, *J =* 5.2 Hz, 3H), 1.13 (d, *J =* 6.3 Hz, 3H); ^13^C NMR (101 MHz, CDCl_3_): δ 156.77, 151.29, 145.81, 137.99, 135.70, 134.73, 129.73, 126.18, 121.86, 121.61, 116.93, 72.19, 72.03, 64.51, 59.84, 58.45, 57.26, 51.63, 48.16, 40.44, 29.73, 29.20, 25.47, 21.26, 19.08, 18.90; HRMS calculated for C_26_H_37_N_4_O 421.3478; found: 421.3472 [M + H].

##### *(S)*-N-((*(R)*-5-((1*R*,5*S*)-8-Oxa-3-azabicyclo­[3.2.1]­octan-3-yl)-1,2,3,4-tetrahydroisoquinolin-3-yl)­methyl)-*N*-methyl-5,6,7,8-tetrahydroquinolin-8-amine (**74**)

Procedure A was used, starting with **70d**, to afford a 38% yield of a white foam.

^1^H NMR (400 MHz, CDCl_3_): δ 8.46 (dd, 1H, J = 1, 5 Hz), 7.33 (dd, 1H, J = 1, 8 Hz), 7.06 (d, 1H, J = 9 Hz), 7.05 (q, 1H, J = 5 Hz), 6.89 (d, 1H, J = 8 Hz), 6.76 (d, 1H, J = 8 Hz), 4.39 (d, 1H, J = 6 Hz), 4.30 (d, 1H, J = 6 Hz), 3.97 (ABq, 2H, J = 15 Hz, v = 38 Hz), 3.95 (m, 1H), 3.25 (dd, 1H, J = 1.6, 11 Hz), 2.69–2.87 (m, 7H), 2.60 (q, 2H, J = 11 Hz), 2.49 (s, 3H), 1.89–2.23 (m, 9H), 1.70 (m, 1H); ^13^C NMR (101 MHz, CDCl_3_): δ 158.06, 150.44, 146.81, 136.87, 136.68, 133.79, 130.35, 126.16, 122.20, 121.54, 117.83, 75.15, 74.91, 64.50, 60.14, 57.17, 56.41, 51.63, 48.75, 41.18, 30.18, 29.20, 28.56, 28.30, 26.21, 21.24; LC-MS: 100% @ 3.35 min for 419.4 *m*/*z* (M+H)^+^; HRMS calculated for C_26_H_35_ON_4_ 419.28054; found 419.28001 [M + H].

##### (*(S)*-4-(*(R)*-3-((Methyl­(*(S)*-5,6,7,8-tetrahydroquinolin-8-yl)­amino)­methyl)-1,2,3,4-tetrahydroisoquinolin-5-yl)­morpholin-2-yl)­methanol (**75**)

Procedure A was used, starting with **70e**, to afford a 55% yield of a white foam.

^1^H NMR (400 MHz, CDCl_3_): δ 8.42 (dd, *J =* 4.7, 1.7 Hz, 1H), 7.45–7.41 (m, 1H), 7.20 (t, *J =* 7.8 Hz, 1H), 7.14 (dd, *J =* 7.7, 4.8 Hz, 1H), 7.00–6.91 (m, 1H), 6.87 (d, *J =* 7.6 Hz, 1H), 4.39 (d, *J =* 15.8 Hz, 1H), 4.22 (d, *J =* 15.8 Hz, 1H), 4.12–3.97 (m, 2H), 3.88–3.73 (m, 2H), 3.73–3.56 (m, 2H), 3.33–2.93 (m, 6H), 2.89–2.62 (m, 5H), 2.52–2.44 (m, 1H), 2.31 (s, 3H), 2.15–1.83 (m, 2H), 1.82–1.66 (m, 1H), 1.39–1.02 (m, 2H); ^13^C NMR (151 MHz, CDCl_3_) δ 157.28, 151.04, 146.54, 137.64, 134.42, 131.53, 128.08, 127.19, 122.14, 121.92, 118.00, 76.53, 67.00, 64.64, 63.70, 58.39, 54.23, 52.34, 51.22, 45.00, 29.71, 29.14, 26.63, 24.32, 21.37; HRMS calculated for C_25_H_35_N_4_O_2_: 423.27545; found 423.27517 [M + H].

##### (*(R)*-4-(*(R)*-3-((Methyl­(*(S)*-5,6,7,8-tetrahydroquinolin-8-yl)­amino)­methyl)-1,2,3,4-tetrahydroisoquinolin-5-yl)­morpholin-2-yl)­methanol (**76**)

Procedure A was used, starting with **70e,** to afford a 87% yield of a white foam.

^1^H NMR (600 MHz, CDCl_3_): δ 8.48 (d, *J =* 3.2 Hz, 1H), 7.35 (dd, *J =* 7.7, 1.6 Hz, 1H), 7.10 (t, *J =* 7.8 Hz, 1H), 7.07 (dd, *J =* 7.7, 4.7 Hz, 1H), 6.85 (d, *J =* 7.8 Hz, 1H), 6.79 (d, *J =* 7.6 Hz, 1H), 4.06 (A of AB, *J*_*AB*_ = 15.4 Hz, 1H), 3.98–3.92 (m, 3H), 3.83 (td, *J =* 10.8, 2.5 Hz, 1H), 3.80–3.74 (m, 2H), 3.69–3.65 (m, 1H), 2.96–2.74 (m, 8H), 2.69 (dt, *J =* 16.4, 4.7 Hz, 1H), 2.59 (td, *J =* 12.4, 11.4, 3.0 Hz, 1H), 2.52 (s, 3H), 2.25 (br s, 1H), 2.18–2.12 (m, 1H), 2.10–1.92 (m, 3H), 1.77–1.67 (m, 1H), 1.70 (br s, 1H); ^**13**^C NMR (151 MHz, CDCl_3_): δ 158.09, 150.83, 146.85, 137.02, 136.66, 133.87, 130.06, 126.11, 122.11, 121.54, 116.72, 76.30, 66.88, 64.20, 63.81, 59.88, 52.73, 52.29, 51.58, 48.83, 41.50, 30.39, 29.26, 25.99, 21.32; LC-MS (ESI-API, 254 nm) 10–95% MeOH in H_2_O (0.1% HCO_2_H), 6 min, 0.6 mL/min, C18 (Agilent Zorbax XDB-C18, 50 mm × 2.1 mm, 3.5 μm), *m*/*z* = 423.5 (M + H), 212.3 (M/2 + H), t = 2.672 min; HRMS calculated for C_25_H_35_O_2_N_4_ 423.27545; found: 423.27599 [M + H].

##### *(S)*-N-((*(R)*-5-((1*R*,4*R*)-2-Oxa-5-azabicyclo­[2.2.1]­heptan-5-yl)-1,2,3,4-tetrahydroisoquinolin-3-yl)­methyl)-*N*-methyl-5,6,7,8-tetrahydroquinolin-8-amine (**77**)

Procedure A was used, starting with **70f,** to afford a 86% yield of a white foam.

^1^H NMR (400 MHz, CDCl_3_): δ 8.43 (dd, 1H, J = 1.6, 4.8 Hz), 7.33 (d, 1H, J = 8 Hz), 7.06 (m, 1H), 7.03 (t, 1H, J = 7.6 Hz), 6.83 (d, 1H, J = 8 Hz), 6.65 (d, 1H, J = 7.6 Hz), 4.55 (s, 1H), 4.11 (m, 2H), 3.98 (m, 1H), 3.88 (d, 1H, J = 7.2 Hz), 3.74 (dd, 1H, J = 2.4, 7.6 Hz), 3.48 (d, 1H, J = 9.6 Hz), 2.86 (m, 2H), 2.76 (m, 2H), 2.67 (m, 1H), 2.44 (s, 3H), 1.99 (m, 2H), 1.92 (m, 2H), 1.89 (m, 2H), 1.70 (m, 1H); ^13^C NMR (100 MHz, CDCl_3_): δ 157.78, 149.39, 146.82, 136.75, 133.89, 127.74, 125.73, 121.57, 119.74, 114.45, 77.79, 77.76, 73.43, 64.50, 60.65, 59.85, 59.77, 51.70, 48.42, 40.65, 35.39, 31.52, 29.26, 25.13, 21.32; LCMS (10–95% MeOH) 99.67% at 254 nm), 96.27% TIC (MW+H^+^ 405m/z); HRMS calculated for C_25_H_33_N_4_O 405.26489; found, 405.26558 [M + H].

##### *(S)*-N-((*(R)*-5-((1*S*,4*R*)-2-Oxa-6-azabicyclo­[2.2.1]­heptan-6-yl)-1,2,3,4-tetrahydroisoquinolin-3-yl)­methyl)-*N*-methyl-5,6,7,8-tetrahydroquinolin-8-amine (**78**)

Procedure A was used, starting with **70g** to afford an 80% yield of a white foam.

^1^H NMR (400 MHz, CDCl_3_): δ 8.42 (dd, 1H, J = 1.6, 4.8 Hz), 7.32 (d, 1H, J = 8 Hz), 7.02 (dd, 1H, J = 4.8, 7.6 Hz), 6.98 (t, 1H, J = 8 Hz), 6.57 (d, 1H, J = 7.2 Hz), 6.49 (d, 1H, J = 8 Hz), 4.51 (s, 1H), 4.22 (d, 1H, J = 7.6 Hz), 4.06 (d, 2H, J = 9.2 Hz), 3.94 (m, 2H), 3.83 (dd, 1H, J = 1.6, 7.6 Hz), 3.65 (dd, 1H, J = 2, 9.6 Hz), 2.93 (d, 1H, J = 9.2 Hz), 2.74 (m, 1H), 2.69 (m, 2H), 2.54 (m, 1H), 2.46 (s, 3H), 2.22 (ABX, J = 10, 8 Hz, z = 5.2 Hz), 2.01 (m, 1H), 1.93 (m, 2H), 1.86 (m, 1H), 1.68 (m, 1H); ^13^C NMR (100 MHz, CDCl_3_): δ 157.88, 147.89, 146.83, 136.86, 136.71, 133.93, 125.56, 125.44, 121.56, 118.36, 113.22, 76.99, 76.96, 71.05, 64.44, 60.19, 59.70, 51.67, 48.69, 40.96, 36.33, 32.49, 29.25, 25.36, 21.30; LCMS: (75–95% ACN): 95.82% (254 nm); 99.07% TIC (M+H^+^ for 405m/z); HRMS calculated for C_25_H_33_N_4_O 405.26489; found, 405.26552.

##### *(S)*-*N*-Methyl-N-((*(R)*-5-(*(R)*-3-methylmorpholino)-1,2,3,4-tetrahydroisoquinolin-3-yl)­methyl)-5,6,7,8-tetrahydroquinolin-8-amine (**79**)

Procedure A was used, starting with **70j,** to afford a 37% yield of a white foam.

^1^H NMR (400 MHz, CDCl_3_): δ 8.50 (dd, *J =* 4.8, 1.7 Hz, 1H), 7.37 (dd, *J =* 7.7, 1.7 Hz, 1H), 7.15–7.02 (m, 3H), 6.87 (d, *J =* 7.4 Hz, 1H), 4.08–3.90 (m, 3H), 3.84 (ddd, *J =* 14.2, 9.9, 2.8 Hz, 3H), 3.73 (td, *J =* 10.9, 2.5 Hz, 1H), 3.31 (dd, *J =* 10.9, 9.6 Hz, 1H), 3.19–3.04 (m, 1H), 2.85–2.77 (m, 4H), 2.76–2.64 (m, 3H), 2.59 (s, 3H), 2.48 (dd, *J =* 13.1, 10.1 Hz, 1H), 2.38–2.25 (m, 1H), 2.17–1.87 (m, 3H), 1.81–1.61 (m, 1H), 0.65 (d, *J =* 6.2 Hz, 3H); ^13^C NMR (101 MHz, CDCl_3_): δ 158.25, 149.57, 146.84, 136.87, 136.67, 133.89, 133.32, 125.96, 123.61, 121.55, 120.73, 73.63, 67.95, 64.47, 59.63, 54.51, 51.69, 48.76, 41.75, 30.09, 29.72, 29.27, 26.79, 21.40, 14.89; HRMS calculated for C_25_H_35_NO 407.28054; found 407.28068 [M + H].

##### *(S)*-*N*-Methyl-N-((*(R)*-5-(*(S)*-3-methylmorpholino)-1,2,3,4-tetrahydroisoquinolin-3-yl)­methyl)-5,6,7,8-tetrahydroquinolin-8-amine (**80**)

Procedure A was used, starting with **70k,** to afford a 73% yield of white foam.

^1^H NMR (400 MHz, CDCl_3_): δ 8.49 (dd, *J =* 4.8, 1.7 Hz, 1H), 7.39 (dd, *J =* 7.7, 1.7 Hz, 1H), 7.14 (d, *J =* 7.7 Hz, 1H), 7.10 (dd, *J =* 7.8, 4.8 Hz, 1H), 6.97 (d, *J =* 7.8 Hz, 1H), 6.85 (d, *J =* 7.5 Hz, 1H), 4.14 (d, *J =* 15.3 Hz, 1H), 4.05–3.96 (m, 2H), 3.81 (dddd, *J =* 28.2, 20.4, 10.9, 2.8 Hz, 3H), 3.53–3.31 (m, 2H), 3.23 (dqd, *J =* 9.2, 6.2, 2.9 Hz, 1H), 3.04 (dd, *J =* 16.6, 3.2 Hz, 1H), 2.96–2.77 (m, 4H), 2.77–2.58 (m, 2H), 2.55 (s, 3H), 2.26–1.88 (m, 5H), 1.74 (dddd, *J =* 13.3, 10.4, 8.7, 4.5 Hz, 1H), 0.79 (d, *J =* 6.2 Hz, 3H); ^13^C NMR (101 MHz, CDCl_3_): δ 158.16, 149.13, 146.79, 136.83, 136.25, 133.97, 131.94, 125.94, 122.68, 121.64, 119.91, 73.31, 67.88, 64.45, 59.67, 52.75, 51.92, 50.57, 48.53, 30.45, 29.77, 29.25, 26.47, 21.38, 14.26; HRMS calculated for C_25_H_35_NO 407.28054; found 407.28014 [M + H].

##### 4-(*(R)*-3-((Methyl­(*(S)*-5,6,7,8-tetrahydroquinolin-8-yl)­amino)­methyl)-1,2,3,4-tetrahydroisoquinolin-5-yl)­morpholin-3-one (**81**)

Procedure A was used, starting with **70i,** to afford an 89% yield of a white foam (3:1 mixture of atropisomers).

^1^H NMR (600 MHz, CDCl_3_): δ 8.42 (dd, *J =* 4.7, 1.8 Hz, 1H), 7.31 (dd, *J =* 7.5, 1.7 Hz, 1H), 7.15 (t, *J =* 7.9 Hz, 0.25H), 7.14 (t, *J =* 7.9 Hz, 0.75H), 7.03 (dd, *J =* 7.8, 4.5 Hz, 0.75H), 7.04–6.98 (m, 0.5H), 7.00 (d, *J =* 7.7 Hz, 0.75H), 6.98 (d, *J =* 7.6 Hz, 0.25H), 6.94 (d, *J =* 7.7 Hz, 0.75H), 4.31 (A of AB, *J*_*AB*_ = 17.5 Hz, 0.25H), 4.29 (s, 1.5H), 4.25 (B of AB, *J*_*AB*_ = 16.5 Hz, 0.25H), 4.07 (A of AB, *J*_*AB*_ = 15.8 Hz, 0.25H), 4.02–3.89 (m, 4H), 3.83 (B of AB, *J*_*AB*_ = 15.2 Hz, 0.75H), 3.69 (dt, *J =* 12.1, 5.2 Hz, 0.25H), 3.53 (ddd, *J =* 12.1, 6.5, 4.1 Hz, 0.75H), 3.50–3.42 (m, 1H), 3.19 (br s, 2H), 2.96–2.89 (m, 0.25H), 2.81–2.71 (m, 2.75H), 2.64 (d, *J =* 15.8 Hz, 1H), 2.55–2.42 (m, 1H), 2.46 (d, *J =* 1.5 Hz, 2.25H), 2.42 (s, 0.75H), 2.26 (dd, *J =* 16.7, 10.8 Hz, 0.25H), 2.16 (dd, *J =* 16.2, 10.9 Hz, 0.75H), 2.05–1.82 (m, 3H), 1.72–1.61 (m, 1H); ^13^C NMR (151 MHz, CDCl_3_): δ 166.31, 165.83 (minor isomer), 157.65, 157.58 (minor isomer), 146.66 (both isomers), 139.91 (minor isomer), 139.74, 137.66, 137.41 (minor isomer), 136.64, 136.59 (minor isomer), 133.86 (minor isomer), 133.66, 132.40 (minor isomer), 131.69, 126.73, 126.68, 126.57 (minor isomer), 126.15 (minor isomer), 124.77, 124.10 (minor isomer), 121.47, 121.44 (minor isomer), 68.27, 68.25 (minor isomer), 64.39 (minor isomer), 64.27, 64.05 (minor isomer), 63.93, 59.81 (minor isomer), 59.48, 51.13 (minor isomer), 50.95, 49.92 (minor isomer), 49.25, 48.46, 48.13 (minor isomer), 41.22, 40.16 (minor isomer), 29.29 (minor isomer), 29.10 (minor isomer), 29.06 (two signals), 25.44 (minor isomer), 25.32, 21.20 (minor isomer), 21.15; LC-MS (ESI-API, 254 nm) 50–95% MeOH in H_2_O (0.1% HCO_2_H), 3 min, 1.00 mL/min, C18 (Agilent Zorbax XDB-18, 50 mm × 4.6 mm, 3.5 μm), *m*/*z* = 429.2 (M + Na), 407.2 (M + H), 204.2 (M/2 + H), t = 0.460 min; HRMS calculated for C_24_H_31_O_2_N_4_ 407.2442; found: 407.2440 [M + H].

##### *(S)*-*N*-Methyl-N-((*(R)*-2-methyl-5-morpholino-1,2,3,4-tetrahydroisoquinolin-3-yl)­methyl)-5,6,7,8-tetrahydroquinolin-8-amine (**82**)

Procedure C was used, starting with paraformaldehyde and **45**. The crude product was purified with column chromatography, starting with DCM and increasing the polarity with DCM:MeOH:NH_3_ (7N in MeOH) (90:10:2), to afford a 91% yield of a yellowish foam.

^1^H NMR (400 MHz, CDCl_3_): δ 8.41 (d, *J =* 4.9 Hz, 1H), 7.30 (d, *J =* 7.8 Hz, 1H), 7.08 (t, *J =* 7.7 Hz, 1H), 7.00 (dd, *J =* 7.7, 4.7 Hz, 1H), 6.85 (d, *J =* 7.9 Hz, 1H), 6.73 (d, *J =* 7.5 Hz, 1H), 3.84 (t, *J =* 4.5 Hz, 5H), 3.80–3.74 (m, 1H), 3.75 (A of AB, *J*_AB_ = 17.5 Hz, 2H), 3.68 (B of AB, *J*_AB_ = 15.8 Hz, 2H), 2.98–2.75 (m, 8H), 2.70–2.58 (m, 2H), 2.48 (dd, *J =* 12.2, 7.5 Hz, 1H), 2.41 (s, 3H), 2.28 (s, 3H), 2.08–1.95 (m, 1H), 1.95–1.87 (m, 2H), 1.68–1.57 (m, 1H); ^13^C NMR (100 MHz, CDCl_3_): δ 157.64, 151.02, 146.49, 136.37, 135.43, 133.68, 129.12, 129.10, 125.89, 121.57, 121.39, 116.49, 67.30, 64.86, 57.02, 56.35, 55.94, 52.12, 40.72, 39.49, 28.60, 27.33, 25.76, 20.09; HRMS calculated for C_25_H_35_N_4_O 407.2805; found: 407.2806 [M + H].

##### *tert*-Butyl*(R)*-5-bromo-3-((((3,5-dimethylpyridin-2-yl)­methyl)­(methyl)­amino)­methyl)-3,4-dihydroisoquinoline-2­(1*H*)-carboxylate (**84**)

Procedure C was used, starting with **83** ^52^ and **68,** to afford a 57% yield of a white foam.

***(R)*** **-1-(3,5-Dimethylpyridin-2-yl)-** ***N*** **-methyl-N-((5-morpholino-1,2,3,4-tetrahydroisoquinolin-3-yl)­methyl)­methanamine (85)** was synthesized through the following steps.

Step 1.

##### *tert*-Butyl*(R)*-3-((((3,5-dimethylpyridin-2-yl)­methyl)­(methyl)­amino)­methyl)-5-morpholino-3,4-dihydroisoquinoline-2­(1*H*)-carboxylate

Procedure B was used, starting with **84** and morpholine. The crude material was purified by column chromatography using EA:hexanes to afford an 80% yield as a yellow foam.

^1^H NMR (600 MHz, CDCl_3_): δ 7.97 (d, *J =* 7.2 Hz, 1H), 7.24–7.17 (m, 1H), 7.06 (t, *J =* 7.8 Hz, 1H), 6.79 (d, *J =* 7.7 Hz, 1H), 6.63–6.51 (m, 1H), 4.55 (d, *J =* 17.4 Hz, 1H), 4.52–4.41 (m, 1H), 3.83 (ddd, *J =* 9.5, 6.5, 2.4 Hz, 2H), 3.75 (ddd, *J =* 11.1, 6.2, 2.5 Hz, 2H), 3.60–3.49 (m, 2H), 3.45 (d, *J =* 12.2 Hz, 1H), 3.02–2.90 (m, 3H), 2.75–2.57 (m, 3H), 2.33 (s, 3H), 2.24 (s, 3H), 2.23 (s, 3H), 2.10–1.97 (m, 1H), 1.88–1.68 (m, 1H), 1.45 (s, 9H); LC-MS (ESI-API, 254 nm) 75–95% MeOH in H_2_O (0.1% HCO_2_H), 3 min, 1.00 mL/min, C18 (Agilent Zorbax XDB-18, 50 mm × 4.6 mm, 3.5 μm), *m*/*z* = 481.4 (M + 1), purity: **≥**95%.

Step 2.

##### *(R)*-1-(3,5-Dimethylpyridin-2-yl)-*N*-methyl-N-((5-morpholino-1,2,3,4-tetrahydroisoquinolin-3-yl)­methyl)­methanamine (**85**)

Procedure A was used to afford a 70% yield of white foam.

^1^H NMR (400 MHz, CDCl_3_): δ 8.18 (d, *J =* 1.5 Hz, 1H), 7.26 (d, *J =* 2.1 Hz, 1H), 7.11 (t, *J =* 7.8 Hz, 1H), 6.88 (d, *J =* 7.7 Hz, 1H), 6.78 (d, *J =* 7.6 Hz, 1H), 4.25 (br s, 1H), 4.11 (A of AB, *J*_*AB*_ = 15.4 Hz, 1H), 4.06 (B of AB, *J*_*AB*_ = 15.5 Hz, 1H), 3.86–3.74 (m, 4H), 3.70 (A of AB, *J*_*AB*_ = 13.1 Hz, 1H), 3.61 (B of AB, *J*_*AB*_ = 13.0 Hz, 1H), 3.07–2.90 (m, 4H), 2.76–2.69 (m, 2H), 2.65 (dd, *J =* 12.7, 10.3 Hz, 1H), 2.56 (dd, *J =* 12.4, 3.7 Hz, 1H), 2.37–2.22 (m, 1H), 2.36 (s, 3H), 2.31 (s, 3H), 2.26 (s, 3H); ^13^C NMR (100 MHz, CDCl_3_): δ 153.75, 151.02, 146.43, 138.94, 135.41, 131.93, 131.76, 129.36, 126.33, 121.88, 116.95, 67.36, 62.54, 62.28, 52.16, 51.28, 47.63, 43.18, 29.27, 18.22, 17.86; HRMS calculated for C_23_H_33_ON_4_ 381.26489; found: 381.26489 [M + H].

***(R)*** **-4-(3-((((3,5-Dimethylpyridin-2-yl)­methyl)­(methyl)­amino)­methyl)-1,2,3,4-tetrahydroisoquinolin-5-yl)­morpholin-3-one (86)** was synthesized by the following steps.

Step 1.

##### *tert*-Butyl*(R)*-3-((((3,5-dimethylpyridin-2-yl)­methyl)­(methyl)­amino)­methyl)-5-(3-oxomorpholino)-3,4-dihydroisoquinoline-2­(1*H*)-carboxylate

A mixture of **84** (0.33 g, 0.69 mmol), morpholin-3-one (0.08 g, 0.83 mmol), N, N’-dimethylethane-1,2-diamine (0.01 mL, 0.14 mmol), potassium carbonate (0.19 g, 1.39 mmol), and copper­(I) iodide (0.01 g, 0.07 mmol) in anhydrous, degassed toluene (3 mL) was heated to 110 °C with stirring overnight. The reaction mixture was poured into water and extracted with EA. The organic layer was dried over anhydrous MgSO_4_, filtered off, and evaporated. The product was purified with column chromatography with hexanes:EA, 100% EA, and then 10% MeOH in EA.

^1^H NMR (600 MHz, CDCl_3_): δ 8.06 (dd, *J =* 69.2, 2.1 Hz, 1H), 7.24 (t, *J =* 7.8 Hz, 1H), 7.17 (t, *J =* 7.7 Hz, 1H), 7.02 (dd, *J =* 21.8, 7.6 Hz, 1H), 6.85 (d, *J =* 7.7 Hz, 1H), 4.85–4.50 (m, 2H), 4.39–4.25 (m, 2H), 4.00 (dddd, *J =* 18.1, 16.1, 11.9, 6.0 Hz, 3H), 3.88–3.68 (m, 1H), 3.62 (ddd, *J =* 12.1, 6.7, 3.5 Hz, 1H), 3.56 (d, *J =* 12.2 Hz, 1H), 3.52–3.36 (m, 2H), 2.89–2.75 (m, 1H), 2.69–2.40 (m, 2H), 2.35 (s, 3H), 2.27 (t, *J =* 5.1 Hz, 6H), 1.46 (s, 9H).

Step 2.

##### *(R)*-4-(3-((((3,5-Dimethylpyridin-2-yl)­methyl)­(methyl)­amino)­methyl)-1,2,3,4-tetrahydroisoquinolin-5-yl)­morpholin-3-one (**86**)

Procedure A was used to afford a 77% yield of white foam.

^1^H NMR (400 MHz, CDCl_3_): δ 8.22 (d, *J =* 2.2 Hz, 1H), 7.29 (s, 1H), 7.22 (td, *J =* 7.7, 3.8 Hz, 1H), 7.11–6.96 (m, 2H), 4.37 (s, 2H), 4.35 (d, *J =* 3.9 Hz, 1H), 4.11–3.92 (m, 4H), 3.81–3.67 (m, 1H), 3.67–3.55 (m, 2H), 3.50 (dddd, *J =* 17.9, 12.3, 5.7, 3.9 Hz, 1H), 3.03 (tt, *J =* 10.2, 3.6 Hz, 1H), 2.70–2.43 (m, 3H), 2.41 (s, 3H), 2.33–2.27 (m, 7H); ^13^C NMR (101 MHz, CDCl_3_): δ 166.51, 153.74, 146.65, 140.01, 137.64, 132.14, 131.87, 130.80, 127.06, 126.89, 126.36, 125.08, 68.49, 64.12, 62.87, 51.04, 50.13, 49.46, 48.63, 43.23, 29.19, 18.33, 17.96; HRMS calculated for C_23_H_31_N_4_O_2_ 395.24415; found 395.24320 [M + H].

***(S)*** **-N-(*(S)*-1-(4-Methoxyphenyl)­ethyl)-*N*-methyl-3,4-dihydro-2*H*-pyrano­[3,2-*b*]­pyridin-4-amine (87)** was synthesized through the following steps.

Step 1.

##### *(S)*-N-(*(S)*-1-(4-Methoxyphenyl)­ethyl)-3,4-dihydro-2*H*-pyrano­[3,2-*b*]­pyridin-4-amine

To the solution of 2,3-dihydropyrano­[3,2-*b*]­pyridin-4-one (2.38 g, 15.96 mmol) in anhydrous THF (50 mL), titanium­(IV) isopropoxide (9.45 mL, 31.91 mmol) and (1*S*)-1-(4-methoxyphenyl)­ethanamine (2.59 mL, 17.55 mmol) were added and stirred for 4 h at room temperature. STAB (10.68 g, 47.87 mmol) and MeOH (12.5 mL) were added to the reaction mixture and stirred overnight at room temperature. The reaction mixture was quenched with saturated NaHCO_3_ solution and some EA was added. The mixture was stirred for 30 min at room temperature. The phases were separated, and the aqueous phase was extracted with EA 3 times. The combined organic layers were dried over anhydrous Na_2_SO_4_, filtered off, and evaporated. The residue was purified with column chromatography using EA:hexanes. (4S)-N-[rac-(1S)-1-(4-methoxyphenyl)­ethyl]-3,4-dihydro-2H-pyrano­[3,2-*b*]­pyridin-4-amine (1.3 g, 4.57 mmol, 29% yield).

^1^H NMR (400 MHz, CDCl_3_): δ 8.18 (dd, *J =* 3.5, 2.5 Hz, 1H), 7.42–7.34 (m, 2H), 7.12–7.06 (m, 2H), 6.91–6.86 (m, 2H), 4.22 (ddd, *J =* 10.9, 7.7, 3.1 Hz, 1H), 4.09 (t, *J =* 6.6 Hz, 1H), 4.07–3.99 (m, 1H), 3.93 (dd, *J =* 6.3, 4.9 Hz, 1H), 3.83 (s, 3H), 2.30–1.69 (m, 3H), 1.46 (d, *J =* 6.6 Hz, 3H).

Step 2.

##### *(S)*-N-(*(S)*-1-(4-Methoxyphenyl)­ethyl)-*N*-methyl-3,4-dihydro-2*H*-pyrano­[3,2-*b*]­pyridin-4-amine

Procedure C was used, starting with *(S)*-N-(*(S)*-1-(4-methoxyphenyl)­ethyl)-3,4-dihydro-2*H*-pyrano­[3,2-*b*]­pyridin-4-amine and HCHO, to afford *(S)*-N-(*(S)*-1-(4-methoxyphenyl)­ethyl)-*N*-methyl-3,4-dihydro-2*H*-pyrano­[3,2-*b*]­pyridin-4-amine as a yellow foam (100% yield).

^1^H NMR (400 MHz, CDCl_3_): δ 8.24 (dd, *J =* 3.8, 2.3 Hz, 1H), 7.46–7.38 (m, 2H), 7.12–7.04 (m, 2H), 6.93–6.85 (m, 2H), 4.44–4.34 (m, 2H), 4.14–4.04 (m, 2H), 3.82 (s, 3H), 2.31–2.19 (m, 1H), 2.06 (s, 3H), 1.94 (dddd, *J =* 14.2, 7.2, 5.4, 2.9 Hz, 1H), 1.44 (d, *J =* 6.7 Hz, 3H).

Step 3.

##### *(S)*-*N*-Methyl-3,4-dihydro-2H-pyrano­[3,2-*b*]­pyridin-4-amine (**87**)

Procedure A was used to afford **87**, which was carried on as is in the next step.

##### *tert*-Butyl*(R)*-5-bromo-3-(((*(S)*-3,4-dihydro-2H-pyrano­[3,2-*b*]­pyridin-4-yl)­(methyl)­amino)­methyl)-3,4-dihydroisoquinoline-2­(1*H*)-carboxylate (**89**)

Procedure C was used, starting with **68** and **87**, and the crude material was purified with column chromatography, starting with hexanes and increasing the polarity with EA, to afford an 85% yield of a yellow foam.

^1^H NMR (400 MHz, CDCl_3_): δ 8.06 (s, 1H), 7.41 (dd, *J =* 7.8, 1.2 Hz, 1H), 7.10–7.06 (m, 1H), 7.05–6.97 (m, 2H), 6.91 (d, *J =* 25.7 Hz, 1H), 4.80–4.49 (m, 2H), 4.41 (ddd, *J =* 11.3, 7.7, 3.9 Hz, 1H), 4.15 (tt, *J =* 7.2, 4.6 Hz, 2H), 4.02–3.75 (m, 1H), 3.69 (t, *J =* 5.4 Hz, 1H), 3.25–2.95 (m, 1H), 2.81 (dd, *J =* 16.8, 6.2 Hz, 1H), 2.68 (s, 1H), 2.44 (dd, *J =* 12.9, 8.3 Hz, 1H), 2.38 (s, 3H), 1.98 (dddd, *J =* 14.1, 8.3, 4.8, 3.7 Hz, 1H), 1.52 (s, 9H).

##### *tert*-Butyl*(R)*-5-bromo-3-((methyl­(*(S)*-3-methyl-5,6,7,8-tetrahydroquinolin-8-yl)­amino)­methyl)-3,4-dihydroisoquinoline-2­(1*H*)-carboxylate (**90**)

Procedure C was used, starting with **68** and **88** ^52^ and the crude material was purified with column chromatography, starting with hexanes and increasing the polarity with EA, to afford a 46% yield of a white foam.

^1^H NMR (400 MHz, CDCl_3_): δ 8.17 (s, 1H), 7.39 (d, *J =* 7.8 Hz, 1H), 7.13–7.08 (m, 1H), 6.97 (t, *J =* 7.7 Hz, 1H), 6.88 (d, *J =* 7.5 Hz, 1H), 4.64 (s, 1H), 4.52 (s, 1H), 3.78 (d, *J =* 17.8 Hz, 1H), 3.70 (dd, *J =* 7.5, 5.0 Hz, 1H), 3.15 (d, *J =* 16.9 Hz, 1H), 2.85–2.69 (m, 2H), 2.66–2.53 (m, 2H), 2.41 (s, 3H), 2.22 (s, 3H), 2.05–1.78 (m, 2H), 1.64 (ddt, *J =* 21.1, 8.8, 3.6 Hz, 2H), 1.51 (s, 7H), 1.50 (s, 1H); MS (*m*/*z*): 502.3 (M+H^+^, ^81^Br), 500.3 (M+H^+^, ^79^Br).

***(S)*** **-N,3-Dimethyl-N-((** ***(R)*** **-5-morpholino-1,2,3,4-tetrahydroisoquinolin-3-yl)­methyl)-5,6,7,8-tetrahydroquinolin-8-amine (91)** was synthesized with the following steps.

Step 1.

##### *tert*-Butyl*(R)*-3-((methyl­(*(S)*-3-methyl-5,6,7,8-tetrahydroquinolin-8-yl)­amino)­methyl)-5-morpholino-3,4-dihydroisoquinoline-2­(1*H*)-carboxylate

Procedure B was used, starting with **91** and morpholine. The crude material was purified with column chromatography, starting with DCM and increasing the polarity with DCM:MeOH:NH_3_ (7N in MeOH) (90:10:2), to afford a 42% yield of a yellow foam.

^1^H NMR (400 MHz, CDCl_3_): δ 8.08 (s, 2H), 7.23–7.07 (m, 4H), 6.87 (d, *J =* 7.4 Hz, 2H), 6.73–6.63 (m, 2H), 5.32 (s, 1H), 4.58 (d, *J =* 16.8 Hz, 2H), 4.45 (s, 1H), 3.97–3.79 (m, 10H), 3.54 (t, *J =* 6.1 Hz, 2H), 3.25 (d, *J =* 15.9 Hz, 2H), 3.05 (ddd, *J =* 11.8, 6.2, 2.8 Hz, 4H), 2.88–2.56 (m, 12H), 2.27 (d, *J =* 13.3 Hz, 13H), 2.02 (dp, *J =* 12.2, 6.1 Hz, 2H), 1.89 (p, *J =* 3.8 Hz, 4H), 1.59 (dq, *J =* 13.9, 6.5 Hz, 3H), 1.52 (s, 16H); MS (*m*/*z*): 508.4 (M+H^+^), 254.3, 226.3, 146.2; MS (*m*/*z*): 507.4 (M+H^+^), 506.4 (M^+^), 253.8, 225.8, 203.8.

Step 2.

##### *(S)*-N,3-Dimethyl-N-((*(R)*-5-morpholino-1,2,3,4-tetrahydroisoquinolin-3-yl)­methyl)-5,6,7,8-tetrahydroquinolin-8-amine (**91**)

Procedure A was used to afford a 71% yield of white foam.

^1^H NMR (600 MHz, CDCl_3_): δ 8.32 (d, *J =* 2.2 Hz, 1H), 7.20–7.16 (m, 1H), 7.12 (t, *J =* 7.7 Hz, 1H), 6.89 (dd, *J =* 7.9, 1.2 Hz, 1H), 6.81 (dd, *J =* 7.6, 1.1 Hz, 1H), 4.08 (d, *J =* 15.3 Hz, 1H), 3.98–3.90 (m, 2H), 3.83 (dddd, *J =* 35.7, 11.0, 6.3, 2.7 Hz, 4H), 3.02 (ddt, *J =* 9.3, 7.0, 2.9 Hz, 2H), 2.88 (dd, *J =* 16.2, 3.3 Hz, 1H), 2.84–2.62 (m, 5H), 2.54 (s, 3H), 2.54–2.48 (m, 1H), 2.29 (s, 3H), 2.16 (dd, *J =* 16.3, 10.5 Hz, 1H), 2.10–1.92 (m, 2H), 1.71 (tddd, *J =* 10.2, 7.6, 4.8, 1.8 Hz, 1H); ^13^C NMR (151 MHz, CDCl_3_): δ 155.10, 151.10, 147.36, 137.19, 136.97, 133.19, 130.79, 130.11, 126.08, 122.07, 116.69, 67.50, 64.23, 59.87, 52.23, 51.60, 48.80, 41.57, 30.33, 29.14, 26.35, 21.28, 18.01; MS (*m*/*z*): 407.4 (M+H^+^), 262.3, 231.3, 204.3, 146.2; HRMS calculated for C_25_H_35_ON_4_ 407.28054; found: 407.28008 [M + H].

**4-(*(R)*-3-((Methyl­(*(S)*-3-methyl-5,6,7,8-tetrahydroquinolin-8-yl)­amino)­methyl)-1,2,3,4-tetrahydroisoquinolin-5-yl)­morpholin-3-one (92)** was synthesized through the following steps.

Step 1.

##### *tert*-Butyl*(R)*-3-((methyl­(*(S)*-3-methyl-5,6,7,8-tetrahydroquinolin-8-yl)­amino)­methyl)-5-(3-oxomorpholino)-3,4-dihydroisoquinoline-2­(1*H*)-carboxylate

A mixture of **90** (0.28 g, 0.55 mmol), N, N’-dimethylethane-1,2-diamine (0.01 mL, 0.11 mmol), potassium carbonate (0.15 g, 1.1 mmol), and copper­(I)­iodide (0.01 g, 0.05 mmol) in anhydrous, degassed toluene (3 mL) was heated to 110 °C overnight. The reaction mixture was poured iinto water and extracted with EA. The organic layer was dried over anhydrous MgSO_4_, filtered off, and evaporated. The crude material was purified by column chromatography, starting with hexanes and increasing the polarity to 100% EA, then further increasing the polarity to 10%MeOH in EA, to afford 54% yield of a white foam (2:1 atropisomers)

^1^H NMR (400 MHz, CDCl_3_): δ 8.17 (s, 0.36H), 8.06 (s, 0.56H), 7.27–7.13 (m, 2H), 7.06 (d, *J =* 7.5 Hz, 1H), 6.94 (d, *J =* 7.5 Hz, 1H), 4.86–4.45 (m, 2H), 4.37 (d, *J =* 6.0 Hz, 2H), 4.24–4.00 (m, 3H), 3.83–3.51 (m, 1H), 2.93–2.55 (m, 6H), 2.26 (d, *J =* 4.4 Hz, 6H), 2.06–1.78 (m, 1H), 1.71 (s, 4H), 1.51 (s, 2.77H), 150 (s, 6.72H), 1.28 (t, *J =* 7.1 Hz, 1H).

Step 2.

##### 4-(*(R)*-3-((Methyl­(*(S)*-3-methyl-5,6,7,8-tetrahydroquinolin-8-yl)­amino)­methyl)-1,2,3,4-tetrahydroisoquinolin-5-yl)­morpholin-3-one (**92**)

Procedure A was used to afford a 60% yield of white foam.

^1^H NMR (600 MHz, CDCl_3_): δ 8.29 (s, 0.25H), 8.27 (d, *J =* 2.1 Hz, 0.75H), 7.26–7.18 (m, 2H), 7.12–6.99 (m, 2H), 4.35 (s, 1.50H), 4.33 (d, *J =* 6.8 Hz, 0.50H), 4.23–4.12 (m, 1H), 4.07–3.93 (m, 4H), 3.74 (dt, *J =* 11.0, 5.1 Hz, 0.25H), 3.61 (ddd, *J =* 11.3, 6.9, 4.1 Hz, 0.75H), 3.53 (dt, *J =* 12.2, 4.9 Hz, 1H), 2.94 (d, *J =* 11.5 Hz, 1H), 2.89 (dd, *J =* 13.3, 2.9 Hz, 1H), 2.77 (ddt, *J =* 21.5, 15.4, 7.7 Hz, 2H), 2.71–2.52 (m, 3H), 2.46 (s, 3H), 2.44 (s, 1H), 2.29 (s, 3H), 2.09–1.85 (m, 4H), 1.85–1.60 (m, 1H); ^13^C NMR (151 MHz, CDCl_3_): δ 166.58, 166.12 (minor), 154.74, 154.58 (minor), 147.35 (minor), 147.11, 140.15 (minor), 139.96, 137.69, 137.49 (minor), 133.46, 133.42 (minor), 131.37 (minor), 131.19, 127.31, 127.05 (minor), 126.89, 126.41, 125.47, 124.76, 68.47, 64.23 (minor), 64.14, 59.04, 52.99, 51.74, 51.30 (minor), 50.89, 50.08, 48.92, 47.48, 29.71 (minor), 29.09, 28.26 (minor), 28.07, 25.80 (minor), 25.58, 20.54, 17.97; HRMS calculated for C_25_H_33_O_2_N_4_ 421.2598; found 421.26023 [M + H].

**(4*S*)-*N*-Methyl-N-((5-morpholino-1,2,3,4-tetrahydroisoquinolin-3-yl)­methyl)-3,4-dihydro-2*H*-pyrano­[3,2-** ***b*** **]­pyridin-4-amine (93)** was synthesized through the following steps.

Step 1.

##### *tert*-Butyl 3-(((*(S)*-3,4-dihydro-2H-pyrano­[3,2-*b*]­pyridin-4-yl)­(methyl)­amino)­methyl)-5-morpholino-3,4-dihydroisoquinoline-2­(1H)-carboxylate

Procedure B was used, starting with **89** and morpholine. The crude material was purified with column chromatography using EA:hexanes to afford an 81% yield of a white foam.

^1^H NMR (400 MHz, CDCl_3_): δ 8.01 (d, *J =* 18.1 Hz, 1H), 7.18–7.00 (m, 3H), 6.87 (d, *J =* 8.0 Hz, 1H), 6.70 (dd, *J =* 36.6, 7.6 Hz, 1H), 4.85–4.39 (m, 1H), 4.56–4.35 (m, 2H), 4.20–4.04 (m, 1H), 4.03–3.49 (m, 5H), 3.28–2.97 (m, 3H), 2.80–2.51 (m, 3H), 2.38 (t, *J =* 11.1 Hz, 1H), 2.33 (s, 3H), 2.07 (s, 3H), 1.52 (s, 9H), 1.33–1.21 (m, 1H).

Step 2.

##### (4*S*)-*N*-Methyl-N-((5-morpholino-1,2,3,4-tetrahydroisoquinolin-3-yl)­methyl)-3,4-dihydro-2*H*-pyrano­[3,2-*b*]­pyridin-4-amine (**93**)

Procedure A was used to afford a 64% yield of a white foam.

^1^H NMR (600 MHz, CD_3_OD): δ 8.18 (t, *J =* 3.0 Hz, 1H), 7.28 (t, *J =* 7.8 Hz, 1H), 7.25 (d, *J =* 3.0 Hz, 2H), 7.13 (dd, *J =* 8.0, 1.1 Hz, 1H), 7.03 (dd, *J =* 7.7, 1.1 Hz, 1H), 4.44–4.38 (m, 3H), 4.23 (dd, *J =* 10.4, 6.0 Hz, 1H), 4.17 (td, *J =* 11.3, 2.2 Hz, 1H), 3.69 (tt, *J =* 10.5, 4.2 Hz, 1H), 3.29–3.28 (m, 1H), 3.26 (d, *J =* 4.5 Hz, 1H), 3.17–3.10 (m, 2H), 3.05–3.02 (m, 8H), 3.00–2.93 (m, 1H), 2.76 (dd, *J =* 17.3, 10.5 Hz, 1H), 2.66 (s, 3H), 2.27 (dddd, *J =* 14.1, 11.4, 10.3, 3.9 Hz, 1H), 2.11 (s, 3H); ^**13**^C NMR (151 MHz, CD_3_OD): δ 153.66, 150.05, 143.07, 140.78, 129.61, 127.69, 127.02, 125.47, 123.98, 122.73, 119.29, 72.97, 65.39, 61.13, 57.58, 54.25, 51.64, 43.80, 42.98, 33.60, 25.32, 20.51; HRMS calculated for C_24_H_34_N_5_O 408.27579; found 408.27576 [M + H].

**4-(*(R)*-3-(((*(S)*-3,4-Dihydro-2*H*-pyrano­[3,2-*b*]­pyridin-4-yl)­(methyl)­amino)­methyl)-1,2,3,4-tetrahydroisoquinolin-5-yl)­morpholin-3-one (94)** was synthesized through the following steps.

Step 1.

##### *tert*-Butyl*(R)*-3-(((*(S)*-3,4-dihydro-2*H*-pyrano­[3,2-*b*]­pyridin-4-yl)­(methyl)­amino)­methyl)-5-(3-oxomorpholino)-3,4-dihydroisoquinoline-2­(1*H*)-carboxylate

A mixture of *tert*-butyl (3*R*)-5-bromo-3-[[[(4*S*)-3,4-dihydro-2*H*-pyrano­[3,2-*b*]­pyridin-4-yl]-methylamino]­methyl]-3,4-dihydro-1H-isoquinoline-2-carboxylate (0.33 g, 0.67 mmol), morpholin-3-one (0.08 g, 0.8 mmol), N, N’-dimethylethane-1,2-diamine (0.01 mL, 0.13 mmol), potassium carbonate (0.18 g, 1.33 mmol), and copper­(I) iodide (0.01 g, 0.07 mmol) in anhydrous, degassed toluene (3 mL) was heated to 110 °C with stirring overnight. The reaction mixture was poured into water and extracted with EA 2 times. The combined organic layer was dried over anhydrous MgSO_4_, filtered off, and evaporated. The product was purified with column chromatography, first starting with hexanes:EA, then 100% EA, followed by 10%MeOH in EA, to give *tert*-butyl (3R)-3-[[[(4S)-3,4-dihydro-2H-pyrano­[3,2-*b*]­pyridin-4-yl]-methylamino]­methyl]-5-(3-oxomorpholin-4-yl)-3,4-dihydro-1H-isoquinoline-2-carboxylate (0.281 g, 0.55 mmol, 83% yield) as a 2:1 atropisomer mixture.

^1^H NMR (400 MHz, CDCl_3_): δ 8.09 (s, 0.44H), 7.95 (s, 0.88H), 7.27–7.17 (m, 1H), 7.14–7.08 (m, 1H), 7.08–7.01 (m, 3H), 4.43–4.26 (m, 3H), 4.22–4.09 (m, 2H), 4.09–3.98 (m, 3H), 3.93–3.46 (m, 3H), 2.98–2.50 (m, 3H), 2.28 (s, 3H), 1.98 (ddt, *J =* 13.6, 8.5, 4.1 Hz, 1H), 1.69 (d, *J =* 22.0 Hz, 3H), 1.52 (s, 3H), 1.50 (s, 6H).

Step 2.

##### 4-(*(R)*-3-(((*(S)*-3,4-Dihydro-2*H*-pyrano­[3,2-*b*]­pyridin-4-yl)­(methyl)­amino)­methyl)-1,2,3,4-tetrahydroisoquinolin-5-yl)­morpholin-3-one (**94**)

Procedure A was used to afford a 60% yield of a white foam (3:1 mixture of atropisomers).

^1^H NMR (600 MHz, CDCl_3_): δ 8.21­(dd, *J =* 3.9, 2.2 Hz, 0.25H), 8.19 (dd, *J =* 3.9, 2.2 Hz, 0.75H), 7.20 (td, *J =* 7.7, 4.2 Hz, 1H), 7.10 (dd, *J =* 4.9, 2.5 Hz, 2H), 7.07–7.04 (m, 1H), 7.03 (d, *J =* 7.7 Hz, 0.25H), 7.01 (d, *J =* 7.7 Hz, 0.75H), 4.37 (ddd, *J =* 12.6, 6.0, 2.8 Hz, 1H), 4.31 (A of AB, *J*_*AB*_ = 17.5 Hz, 0.25H), 4.29 (s, 1.5H), 4.25 (B of AB, *J*_*AB*_ = 16.5 Hz, 0.25H), 4.07 (A of AB, *J*_*AB*_ = 15.8 Hz, 0.25H), 4.23–3.90 (m, 6H), 3.83 (B of AB, *J*_*AB*_ = 15.2 Hz, 0.75H), 3.73 (ddd, *J =* 12.3, 6.1, 4.3 Hz, 0.25H), 3.59 (ddd, *J =* 12.3, 6.7, 4.0 Hz, 0.75H), 3.54–3.45 (m, 1H), 3.06–2.96 (m, 1H), 2.83 (d, *J =* 12.7 Hz, 0.75H), 2.73 (dd, *J =* 13.0, 4.3 Hz, 0.25H), 2.64–2.50 (m, 2H), 2.46 (s, 2H), 2.45 (s, 1H), 2.41–2.15 (m, 2H), 2.13–2.01 (m, 1H); ^13^C NMR (151 MHz, CDCl_3_): δ 166.52, 166.02 (minor isomer), 152.58, 152.53 (minor isomer), 144.33 (minor isomer), 144.21, 141.39 (minor isomer), 141.22, 140.16 (minor isomer), 140.01, 132.36, 131.58, 127.19 (minor isomer), 126.86, 126.31, 125.28, 124.48, 124.27 (minor isomer), 123.53, 123.46 (minor isomer), 74.86, 68.49, 65.00, 64.25 (minor isomer), 64.13, 60.30 (minor isomer), 60.24, 59.65 (minor isomer), 59.27, 53.42, 51.52, 51.33 (minor isomer), 50.13, 48.96, 25.65, 25.54 (minor isomer); HRMS calculated for C_23_H_29_N_4_O_3_ 409.22342; found 582.39659 [M + H].

**(4a*R*,10b*S*)-1,2,3,4,4a,5,6,10b-Octahydro-1,10-phenanthroline (95)** was synthesized through the following steps.

Step 1.

##### Ethyl 3-(8-oxo-5,6,7,8-tetrahydroquinolin-7-yl)­propanoate

To a solution of 6,7-dihydro-5*H*-quinolin-8-one (12.00 g, 81.54 mmol) in benzene (300 mL) was added p-toluenesulfonic acid monohydrate (1.55 g, 8.15 mmol) and pyrrolidine (13.49 mL, 163.08 mmol). The reaction mixture was heated to reflux using a Dean–Stark trap for 16 h. The reaction mixture was cooled to room temperature and concentrated. The residue was taken up in toluene, concentrated, and dried under vacuum. The residue was dissolved in EtOH (300 mL), and ethyl prop-2-enoate (12.45 mL, 114.15 mmol) was added. The reaction solution was heated to 78 °C for 4 h and allowed to cool to room temperature. The reaction mixture was concentrated to almost half volume, and 150 mL of water was added and extracted with DCM (100 mL) three times. The combined organic layers were dried over anhydrous MgSO_4_, filtered off, and evaporated. The residue was purified with column chromatography, starting with hexanes: EA (1:1) and then slowly increasing the polarity to 100% EA, to give ethyl 3-(8-oxo-6,7-dihydro-5H-quinolin-7-yl)­propanoate (10 g, 40.438 mmol, 50% yield) as a brown oil.

^1^H NMR (500 MHz, CD_3_OD): δ 8.56 (dd, *J =* 4.6, 0.8 Hz, 1H), 7.84 (dd, *J =* 7.9, 0.8 Hz, 1H), 7.52 (dd, *J =* 7.9, 4.6 Hz, 1H), 4.12 (q, *J =* 7.1 Hz, 2H), 3.13–3.06 (m, 2H), 2.76–2.71 (m, 1H), 2.50 (t, *J =* 7.8 Hz, 2H), 2.29–2.24 (m, 2H), 1.98–1.93 (m, 1H), 1.83–1.78 (m, 1H), 1.23 (t, *J =* 7.2 Hz, 3H).

Step 2.

##### Ethyl-3-((7*R*,8*S*)-8-((*(S)*-1-(4-methoxyphenyl)­ethyl)­amino)-5,6,7,8-tetrahydroquinolin-7-yl)­propanoate

To a solution of ethyl 3-(8-oxo-6,7-dihydro-5*H*-quinolin-7-yl)­propanoate (10.00 g, 40.44 mmol) in toluene (350 mL) p-toluenesulfonic acid monohydrate (0.77 g, 4.04 mmol) and rac-(1*S*)-1-(4-methoxyphenyl)­ethanamine (8.96 mL, 60.66 mmol) were added. The reaction mixture was refluxed under a Dean–Stark trap for 4 h. The reaction mixture was cooled to room temperature, evaporated, and the residue was dissolved in DCE (350 mL). STAB (25.71 g, 121.32 mmol) was added and stirred at room temperature overnight. The reaction mixture was diluted with DCE (350 mL) and washed with water. The organic phase was dried over anhydrous Na_2_SO_4_, filtered off, and evaporated. The residue was purified with column chromatography, starting with hexanes:EA (5:1) and then increasing the polarity with EA to give 8.55 g (55% yield) of the desired product.

^1^H NMR (500 MHz, CD_3_OD): δ 8.37 (d, *J =* 4.0 Hz, 1H), 7.54 (d, *J =* 7.5 Hz, 1H), 7.44 (d, *J =* 8.6 Hz, 2H), 7.22 (dd, *J =* 7.7, 4.8 Hz, 1H), 6.89 (d, *J =* 8.8 Hz, 2H), 4.24 (q, *J =* 6.8 Hz, 1H), 4.10 (q, *J =* 7.1 Hz, 2H), 4.06 (d, *J =* 7.2 Hz, 1H), 3.80 (s, 3H), 2.95–2.74 (m, 2H), 2.04–2.00 (m, 1H), 1.88–1.63 (m, 5H), 1.30 (d, *J =* 6.8 Hz, 3H), 1.29–1.25 (m, 1H), 1.23 (t, *J =* 7.1 Hz, 3H).

Step 3.

##### 3-((7*R*,8*S*)-8-((*(S)*-1-(4-Methoxyphenyl)­ethyl)­amino)-5,6,7,8-tetrahydroquinolin-7-yl)­propan-1-ol

To a 0 °C solution of ethyl 3-[(7*R*,8*S*)-8-[[(1S)-1-(4-methoxyphenyl)­ethyl]­amino]-5,6,7,8-tetrahydroquinolin-7-yl]­propanoate (8.55 g, 22.35 mmol) in THF (250 mL) a 2 M solution of lithium aluminum hydride (11.18 mL, 22.35 mmol) in THF was added dropwise. The reaction mixture was stirred for 1 h at 0 °C and quenched with 0.33 mL of water dropwise, then 0.33 mL of 1N NaOH solution, followed by 0.99 mL of water. The mixture was diluted with 250 mL of THF and allowed to warm to room temperature. The reaction mixture was stirred at room temperature for 30 min, and solids were filtered off. The filtrate was concentrated, and the residue was purified using column chromatography, starting with DCM and increasing the polarity with DCM:MeOH:NH_3_ (7N in MeOH) (90:10:2), to give a yellow oil.

Step 4.

##### (4a*R*,10b*S*)-1-(*(S)*-1-(4-Methoxyphenyl)­ethyl)-1,2,3,4,4a,5,6,10b-octahydro-1,10-phenanthroline.

To a solution of 3-[(7*R*,8*S*)-8-[[(1*S*)-1-(4-methoxyphenyl)­ethyl]­amino]-5,6,7,8-tetrahydroquinolin-7-yl]­propan-1-ol (5.60 g, 16.45 mmol) and N,N-diisopropylethylamine (4.30 mL, 24.67 mmol) in DCM (75 mL) was added methanesulfonyl chloride (1.67 mL, 21.38 mmol) slowly, dropwise. 4-Dimethylaminopyridine (0.20 g, 1.64 mmol) was added to the reaction mixture and stirred for 16 h at room temperature. The reaction mixture was diluted with 100 mL of water. The aqueous phase was extracted with DCM 2 times. The combined organic layer was dried over anhydrous Na_2_SO_4_, filtered off, and evaporated. The residue was purified with column chromatography, starting with DCM and increasing the polarity using DCM:MeOH:NH_3_ (7N in MeOH) (90:10:2).

^1^H NMR (500 MHz, CD_3_OD): δ 8.44 (d, *J =* 4.8 Hz, 1H), 7.55 (d, *J =* 7.5 Hz, 2H), 7.49 (d, *J =* 8.6 Hz, 3H), 7.24 (dd, *J =* 7.6, 4.8 Hz, 1H), 6.89 (d, *J =* 8.8 Hz, 2H), 4.46–4.11 (m, 2H), 3.78 (s, 3H), 3.13–2.98 (m, 1H), 2.80–2.55 (m, 2H), 2.50–1.65 (m, 4H).

Step 5.

##### (4a*R*,10b*S*)-1,2,3,4,4a,5,6,10b-Octahydro-1,10-phenanthroline (**95**)

(4a*R*,10b*S*)-1-[(1*S*)-1-(4-Methoxyphenyl)­ethyl]-3,4,4a,5,6,10b-hexahydro-2*H*-1,10-phenanthroline (0.75 g, 2.33 mmol) was dissolved in DCM (5 mL), and TFA (3.58 mL, 46.52 mmol) was added to the reaction solution. It was stirred at room temperature overnight. The reaction mixture was diluted with EA and water and stirred for 30 min. The phases were separated: the organic phase was washed with water, and the combined water phase was basified with 1N NaOH to pH > 12. It was then extracted with DCM several times. The combined organic layer was dried over anhydrous MgSO_4_, filtered off, and evaporated. It was used as is for the next step.

##### *tert*-Butyl*(R)*-5-bromo-3-(((4a*R*,10b*S*)-3,4,4a,5,6,10b-hexahydro-1,10-phenanthrolin-1­(2*H*)-yl)­methyl)-3,4-dihydroisoquinoline-2­(1*H*)-carboxylate (**96**)

Procedure C was used, starting with **68** and **95**. The crude material was purified with column chromatography using EA:hexanes to afford an 85% yield of a yellow foam.

^1^H NMR (400 MHz, CDCl_3_): δ 8.05–7.94 (m, 1H), 7.46 (dd, *J =* 7.7, 1.7 Hz, 1H), 7.32 (dd, *J =* 7.9, 1.2 Hz, 1H), 7.02 (ddd, *J =* 14.0, 7.6, 4.7 Hz, 1H), 6.88 (q, *J =* 8.0 Hz, 1H), 6.55 (dd, *J =* 29.2, 7.7 Hz, 1H), 4.77–4.50 (m, 1H), 4.36 (dd, *J =* 65.0, 17.4 Hz, 1H), 3.32 (d, *J =* 2.9 Hz, 2H), 3.25–2.86 (m, 2H), 2.76 (hept, *J =* 7.5, 6.5 Hz, 4H), 2.60–2.31 (m, 1H), 2.28–2.09 (m, 1H), 2.05–1.76 (m, 3H), 1.75–1.63 (m, 3H), 1.58–1.52 (m, 1H), 1.52 (s, 9H); ^13^C NMR (101 MHz, CDCl_3_): δ 159.20, 154.94, 144.13, 138.48, 135.15, 133.14, 132.10, 129.98, 126.72, 124.49, 122.10, 79.36, 66.09, 57.48, 53.71, 49.04, 46.95, 44.29, 41.73, 40.74, 33.94, 30.36, 28.54, 26.88, 22.80, 13.92.

**4-(*(R)*-3-(((4a*R*,10b*S*)-3,4,4a,5,6,10b-Hexahydro-1,10-phenanthrolin-1­(2*H*)-yl)­methyl)-1,2,3,4-tetrahydroisoquinolin-5-yl)­morpholine (97)** was synthesized with the following steps.

Step 1.

##### *tert*-Butyl*(R)*-3-(((4a*R*,10b*S*)-3,4,4a,5,6,10b-hexahydro-1,10-phenanthrolin-1­(2*H*)-yl)­methyl)-5-morpholino-3,4-dihydroisoquinoline-2­(1*H*)-carboxylate

Procedure B was used, starting with **96** and morpholine. The crude material was purified with column chromatography, starting with DCM and increasing the polarity with DCM:MeOH:NH_3_ (7N in MeOH) (90:10:2), to afford a 50% yield of a white foam.

^1^H NMR (400 MHz, CDCl_3_): δ 8.05–7.90 (m, 1H), 7.50–7.42 (m, 1H), 7.01 (ddt, *J =* 19.8, 11.0, 6.2 Hz, 2H), 6.77 (dd, *J =* 8.0, 4.3 Hz, 1H), 6.31 (dd, *J =* 39.7, 7.6 Hz, 1H), 4.45 (d, *J =* 17.5 Hz, 1H), 4.31 (d, *J =* 17.2 Hz, 1H), 3.93–3.70 (m, 4H), 3.30 (dd, *J =* 17.4, 6.8 Hz, 2H), 3.21–2.94 (m, 4H), 2.94–2.32 (m, 6H), 2.26–2.08 (m, 1H), 2.03–1.79 (m, 2H), 1.68 (s, 6H), 1.52 (s, 9H).

Step 2.

##### 4-(*(R)*-3-(((4a*R*,10b*S*)-3,4,4a,5,6,10b-Hexahydro-1,10-phenanthrolin-1­(2*H*)-yl)­methyl)-1,2,3,4-tetrahydroisoquinolin-5-yl)­morpholine (**97**)

Procedure A was used to afford a 77% yield of white foam.

^1^H NMR (600 MHz, CDCl_3_): δ 8.35 (dd, *J =* 4.8, 1.7 Hz, 1H), 7.39 (dd, *J =* 7.7, 1.6 Hz, 1H), 7.09–7.02 (m, 2H), 6.81 (dd, *J =* 8.0, 1.1 Hz, 1H), 6.70 (dd, *J =* 7.6, 1.1 Hz, 1H), 4.05–3.88 (m, 2H), 3.77 (dddd, *J =* 32.3, 11.0, 6.3, 2.8 Hz, 5H), 3.44 (d, *J =* 3.0 Hz, 1H), 3.04 (ddd, *J =* 16.6, 7.8, 5.3 Hz, 2H), 2.94 (ddd, *J =* 11.9, 6.3, 2.8 Hz, 2H), 2.86–2.76 (m, 2H), 2.76–2.68 (m, 2H), 2.66 (ddd, *J =* 11.7, 6.3, 2.6 Hz, 2H), 2.47 (d, *J =* 12.8 Hz, 1H), 2.36–2.31 (m, 1H), 2.18 (td, *J =* 11.3, 2.9 Hz, 1H), 2.11–1.97 (m, 2H), 1.82–1.68 (m, 2H), 1.68–1.53 (m, 3H); ^**13**^C NMR (151 MHz, CDCl_3_): δ 157.49, 151.08, 145.96, 137.11, 133.63, 126.09, 122.49, 121.94, 116.70, 74.85, 67.51, 67.35, 58.90, 52.19, 51.44, 34.03, 28.99, 27.00, 23.36, 21.90; HRMS calculated for C_26_H_35_N_4_O 419.28054; found 408.28030 [M + H].

**4-(*(R)*-3-(((4a*R*,10b*S*)-3,4,4a,5,6,10b-Hexahydro-1,10-phenanthrolin-1­(2*H*)-yl)­methyl)-1,2,3,4-tetrahydroisoquinolin-5-yl)­morpholin-3-one (98)** was synthesized with the following steps.

Step 1.

##### *tert*-Butyl*(R)*-3-(((4a*R*,10b*S*)-3,4,4a,5,6,10b-hexahydro-1,10-phenanthrolin-1­(2*H*)-yl)­methyl)-5-(3-oxomorpholino)-3,4-dihydroisoquinoline-2­(1*H*)-carboxylate

A mixture of *tert*-butyl (3*R*)-3-[[(4a*R*,10b*S*)-3,4,4a,5,6,10b-hexahydro-2*H*-1,10-phenanthrolin-1-yl]­methyl]-5-bromo-3,4-dihydro-1*H*-isoquinoline-2-carboxylate (0.34 g, 0.67 mmol), morpholin-3-one (0.08 g, 0.8 mmol), N, N’-dimethylethane-1,2-diamine (0.01 mL, 0.13 mmol), potassium carbonate (0.18 g, 1.33 mmol), and copper­(I) iodide (0.01 g, 0.07 mmol) in anhydrous, degassed toluene (3 mL) was heated to 110 °C with stirring overnight. The reaction mxture was poured into water and extracted with EA 2 times. The combined organic layer was dried over anhydrous MgSO_4_, filtered off, and evaporated. The product was purified with column chromatography, first starting with hexanes: EA, then increasing to 100% EA, and then increasing the polarity to 10%MeOH in EA to give *tert*-butyl-(3*R*)-3-[[(4a*R*,10b*S*)-3,4,4a,5,6,10b-hexahydro-2*H*-1,10-phenanthrolin-1-yl]­methyl]-5-(3-oxomorpholin-4-yl)-3,4-dihydro-1*H*-isoquinoline-2-carboxylate (0.189 g, 0.36 mmol, 53% yield) as a mixture of 4:1 atropisomers.

^1^H NMR (400 MHz, CDCl_3_): δ 8.16 (s, 0.25H), 8.04–7.94 (m, 1H), 7.48 (d, *J =* 7.7 Hz, 1H), 7.41 (d, *J =* 7.6 Hz, 0.22H), 7.18–6.91 (m, 3H), 6.85–6.70 (m, 0.23H), 6.61 (dd, *J =* 29.4, 7.7 Hz, 1H), 4.81–4.44 (m, 2H), 4.44–4.23 (m, 3H), 4.09–3.91 (m, 2H), 3.78 (dt, *J =* 10.8, 5.1 Hz, 1H), 3.65–3.24 (m, 2H), 3.24–2.95 (m, 1H), 2.93–2.62 (m, 3H), 2.57–2.27 (m, 2H), 2.07 (s, 1H), 1.89 (dd, *J =* 38.3, 20.5 Hz, 2H), 1.76–1.51 (m, 6H), 1.51 (s, 6.75H), 1.50 (s, 2.25H).

Step 2.

##### 4-(*(R)*-3-(((4a*R*,10b*S*)-3,4,4a,5,6,10b-Hexahydro-1,10-phenanthrolin-1­(2*H*)-yl)­methyl)-1,2,3,4-tetrahydroisoquinolin-5-yl)­morpholin-3-one (**98**)

Procedure A was used to afford a 31% yield of a white foam (3:1 atropisomer mixture).

^1^H NMR (600 MHz, CDCl_3_): δ 8.41 (dd, *J =* 4.9, 1.6 Hz, 0.25H), 8.39 (dd, *J =* 4.7, 1.6 Hz, 0.75H), 7.42 (dd, *J =* 7.6, 1.6 Hz, 1H), 7.17 (t, *J =* 7.8 Hz, 1H), 7.11 (td, *J =* 7.2, 4.7 Hz, 1H), 7.03–6.96 (m, 2H), 4.74 (s, 1.5H), 4.37 (dd, *J =* 5.0, 2.5 Hz, 0.25H), 4.34 (d, *J =* 2.2 Hz, 1.75H), 4.32 (d, *J =* 4.9 Hz, 0.25H), 4.29 (s, 0.25H), 4.10–3.92 (m, 5H), 3.76–3.69 (ddd, *J =* 11.6, 7.2, 4.0 Hz, 0.25H), 3.58 (ddd, *J =* 11.6, 7.2, 4.0 Hz, 0.75H), 3.54–3.38 (m, 2H), 3.06 (m, 2H), 3.00–2.91 (m, 0.75H), 2.91–2.81 (m, 0.25H), 2.85–2.64 (m, 3H), 2.55 (dd, *J =* 16.2, 3.7 Hz, 0.75H), 2.48 (dd, *J =* 16.2, 3.7 Hz, 0.25H), 2.43–1.99 (m, 3H), 1.82–1.57 (m, 5H); ^13^C NMR (151 MHz, CDCl_3_): δ 166.48, 165.93­(minor isomer), 157.45, 157.35­(minor isomer), 146.13­(minor isomer), 145.90, 140.10­(minor isomer), 139.99, 137.13, 136.98­(minor isomer), 133.67, 133.45­(minor isomer), 132.07, 126.85­(minor isomer), 126.82, 126.58, 126.55­(minor isomer), 124.87, 124.46, 124.30 (minor isomer), 122.53, 122.42­(minor isomer), 74.85, 68.47, 67.37, 66.92 (minor isomer), 64.23 (minor isomer), 63.60, 58.93, 51.27, 50.92 (minor isomer), 49.83 (minor isomer), 49.42, 34.13, 31.24, 29.31, 29.12 (minor isomer), 27.10, 26.97, 23.47 (minor isomer), 23.14, 22.03 (minor isomer), 21.82; HRMS calculated for C_26_H_33_N_4_O_2_ 433.2598; found 433.25934 [M + H].

##### *tert*-Butyl 5-morpholino-3-((quinolin-8-ylamino)­methyl)-3,4-dihydroisoquinoline-2­(1H)-carboxylate (**100**)

Procedure C was used, starting with **99** and **32**. The crude material was purified with column chromatography using EA:hexanes to afford a 51% yield of a light yellow foam.

^1^H NMR (600 MHz, CDCl_3_): δ 8.69 (s, 1H), 8.04 (s, 1H), 7.36 (s, 1H), 7.31 (t, *J =* 7.8 Hz, 1H), 7.22 (t, *J =* 7.8 Hz, 1H), 7.05–6.98 (m, 1H), 6.96 (d, *J =* 8.0 Hz, 1H), 6.94–6.86 (m, 1H), 6.69 (d, *J =* 7.6 Hz, 1H), 6.39 (s, 1H), 4.88 (s, 0.4H), 4.76 (s, 0.6H), 4.71–4.55 (m, 1H), 4.47–4.35 (m, 1H), 3.81 (s, 4H), 3.39 (s, 1H), 3.35 (dd, *J =* 15.7, 2.3 Hz, 1H), 3.01 (s, 2H), 2.97 (s, 1H), 2.80 (s, 2H), 2.71 (dd, *J =* 15.7, 5.6 Hz, 1H), 1.52 (s, 9H); ^13^C NMR (151 MHz, CDCl_3_): δ 155.14, 151.30, 146.73, 144.34, 138.09, 135.87, 134.67, 128.68, 127.94, 127.52, 126.98, 121.81, 121.47, 117.08, 113.69, 104.36, 80.37, 67.21, 52.48, 48.67, 48.33, 44.31, 44.11, 43.91, 28.49, 26.70; LC-MS (ESI-API, 254 nm) 95% MeOH in H_2_O (0.1% HCO_2_H), 3 min, 1.00 mL/min, C18 (Agilent Zorbax XDB-18, 50 mm × 4.6 mm, 3.5 μm), *m*/*z* = 475.2 (M + H), t = 1.145 min.

**(*R*)-*N*-Methyl-*N*-((5-morpholino-1,2,3,4-tetrahydroisoquinolin-3-yl)­methyl)­quinolin-8-amine (101)** was synthesized through the following steps.

Step 1.

##### *tert*-Butyl 3-((methyl­(quinolin-8-yl)­amino)­methyl)-5-morpholino-3,4-dihydroisoquinoline-2­(1H)-carboxylate

Procedure C was used, starting with **100** and HCHO (37% aqueous solution). The crude material was purified with column chromatography using EA:hexanes to afford a 51% yield of a yellow foam.

^1^H NMR (600 MHz, CDCl_3_): δ 8.68 (s, 0.5H), 8.53 (s, 0.5H), 8.05 (d, *J =* 8.2 Hz, 1H), 7.38 (t, *J =* 7.8 Hz, 1H), 7.31–7.26 (m, 2H), 7.10–7.00 (m, 2H), 6.77–6.70 (m, 1.5H), 6.47 (s, 0.5H), 4.96 (s, 0.5H), 4.68 (s, 0.5H), 4.63 (d, *J =* 16.7 Hz, 0.5H), 4.38 (d, *J =* 17.0 Hz, 0.5H), 4.20 (s, 0.5H), 3.99 (d, *J =* 16.6 Hz, 0.5H), 3.80–3.50 (m, 5.5H), 3.44 (d, *J =* 17.0 Hz, 0.5H), 3.18 (s, 1.5H), 3.15 (s, 1.5H), 3.03 (d, *J =* 15.8 Hz, 0.5H), 2.94 (d, *J =* 16.1 Hz, 0.5H), 2.88–2.78 (m, 2H), 2.68–2.47 (m, 3H), 1.46 (s, 4.5H), 1.44 (s, 4.5H).

Step 2.

##### (*R*)-*N*-Methyl-*N*-((5-morpholino-1,2,3,4-tetrahydroisoquinolin-3-yl)­methyl)­quinolin-8-amine (**101**)

Procedure C was used to afforda 37% yield of yellow foam.

^1^H NMR (600 MHz, CDCl_3_): δ 8.89 (d, *J =* 3.1 Hz, 1H), 8.11 (d, *J =* 8.3 Hz, 1H), 7.47–7.40 (m, 2H), 7.38 (dd, *J =* 8.2, 4.2 Hz, 1H), 7.25 (d, *J =* 8.2 Hz, 1H), 7.11 (t, *J =* 7.7 Hz, 1H), 6.87 (d, *J =* 7.9 Hz, 1H), 6.80 (d, *J =* 7.6 Hz, 1H), 4.11 (s, 2H), 3.82–3.70 (m, 5H), 3.58 (d, *J =* 12.6 Hz, 1H), 3.26 (s, 1H), 3.14 (s, 3H), 2.98–2.93 (m, 2H), 2.91 (dd, *J =* 16.3, 3.5 Hz, 1H), 2.71–2.66 (m, 2H), 2.24 (t, *J =* 13.6 Hz, 1H), 1.60 (br s, 1H); ^**13**^C NMR (151 MHz, CDCl_3_): δ 151.12, 149.76, 147.83, 142.91, 137.00, 136.50, 129.98, 129.76, 126.64, 126.19, 121.86, 121.13, 120.92, 117.08, 116.68, 67.41, 62.52, 52.40, 52.20, 48.82, 42.13, 30.36; LC-MS (ESI-API, 254 nm) 50–95% MeOH in H_2_O (0.1% HCO_2_H), 6 min, 0.5 mL/min, C18 (Agilent Zorbax XDB-C18, 50 mm × 2.1 mm, 3.5 μm), *m*/*z* = 389.5 (M + H), 195.3 (M/2 + H), t = 0.816 min; HRMS calculated for C_24_H_29_ON_4_ 389.23359; found: 389.23312 [M + H].

## Supplementary Material





## Notes

NMR spectra (^1^H, ^13^C) and HPLC traces for final compounds **28, 42, 45, 75**, and **81** used in the mouse PK experiments and statement of animal care in mouse PK experiments (PDF)
Molecular formula strings and assay data (CSV)

## Abbreviations

070: AMD11070
AcOH: acetic acid
ADMET: absorption, distribution, metabolism, elimination, and toxicity as pharmacokinetic properties
API: atmospheric pressure ionization
AUC: area under the curve
BH3.Me2S: borane dimethyl sulfide
BINAP: (2,2′-bis­(diphenylphosphino)-1,1′-binaphthyl)
Boc: *tert*-butyloxycarbonyl
BSA: bovine serum albumin
C8h: serum concentration at the eighth hour
Caco-2: Human Colon Adenocarcinoma cell line
cAMP: cyclic adenosine monophosphate
Cbz: benzyloxycarbonyl protecting group
CCR5: C–C chemokine receptor type 5
CD8: cluster of differentiation 8
CL: clearance
Cmax: maximum reached serum concentration
cLogD: calculated LogD
CXCL12: stromal Cell-Derived Factor-1
CXCR4: C-X-C chemokine receptor type 4
CYP or CYP 450: cytochrome P450 enzyme
DCE: 1,2-dichloroethane
DCM: dichloromethane
DIBAL-H: diisobutylaluminum hydride
DMAP: 4-dimethylaminopyridine
DMF: dimethylformamide
DMP: Dess-Martin periodinane
DMSO: dimethyl sulfoxide
EA: ethyl acetate
EDCI: N-(3-(dimethylamino)­propyl)-N′-ethylcarbodiimide hydrochloride
ESI: electrospray ionization
Et: ethyl
F: oral bioavailability
(HCHO): formaldehyde
GPCR: G-protein coupled receptor
H_2_SO_4_: sulfuric acid
HBA: hydrogen bond acceptor
HBD: hydrogen bond donor
HEPES: 4-(2-hydroxyethyl)-1-piperazine ethanesulfonic acid
hERG: human Ether-à-go-go-Related Gene
HIV-1: human immunodeficiency virus type 1
HPLC: high-performance liquid chromatography
HRMS: high-resolution mass spectrometry
HSC: hematopoietic stem cell
IC_50_: inhibition concentration for half of biological materials population
IV: intravenous
LC-MS: liquid chromatography–mass spectrometry
LiOH.H_2_O: lithium hydroxide monohydride
LRf: lower Rf
MeOH: methanol
Mpk: milligrams per kilogram
MW: molecular weight
n-bu: *n*-butyl
n-pr: n-propyl
NADP: nicotinamide adenine dinucleotide phosphate
NADPH: reduced form of NADP+
NaOH: sodium hydroxide
NMR: nuclear magnetic resonance spectroscopy
PAMPA: parallel artificial membrane permeability assay
Pd2­(dba)­3: tris­(dibenzylideneacetone)­dipalladium­(0)
Phth: phthalimide protecting group
PK: Pharmacokinetic
Py-SO_3_: pyridine-sulfur trioxide complex
SAR: structure–activity relationship
STAB: sodium triacetoxyborohydride
T1/2: half-life
TFA: trifluoroacetic acid
THF: tetrahydrofuran
THIQ/TIQ: tetrahydroisoquinoline
THN: tetrahydronaphyridine
THQ: tetrahydroquinoline
Ti­(OiPr)_4_: titanium isopropoxide
TMSNCO: trimethylsilyl isocyanate
TRIS-base: 2-amino-2-(hydroxymethyl)-1,3-propanediol
URf: upper Rf
UV: ultraviolet
V: volume of distribution
