# Supplementary material for: Discovery of Novel 2‑Morpholine Tetrahydroisoquinoline CXCR4 Antagonists with Unique Properties
Source: J Med Chem. 2026 Apr 6;69(8):9009–50. doi: 10.1021/acs.jmedchem.5c03471 (PMC13126686; doi:10.1021/acs.jmedchem.5c03471)
Supplement: Supplementary file 1 [file jm5c03471_si_001.pdf]

Supplementary Materials for
Discovery of Novel 2-Morpholine Tetrahydroisoquinoline CXCR4 Antagonists with Unique Properties

Yesim Altas Tahirovic,[§] Zafer Sahin,[§] Edgars Jecs,[§] Eric J. Miller,[§] Huy H. Nguyen,[§] Robert J. Wilson,[§] Michelle Kim,[§] Marshall Fritz,[§] Yusr Zaghlula,[§] Savita K. Sharma,[§] Perry Bartsch,[§] Tao Wang,[‡] Chi S. Sum,[‡] Mary E. Cvijic,[‡] Anthony A. Paiva,[‡] Gretchen M. Schroeder,[‡] Lawrence J. Wilson,*[§] Dennis C. Liotta *[§]

§Department of Chemistry, Emory University, 1515 Dickey Drive NE, Atlanta, GA 30322, United States

‡Bristol-Myers Squibb Research & Development, Princeton, NJ 08543, United States

Corresponding author e-mails: wilsolj@emory.edu; dliotta@emory.edu

**Table of Contents**	**Page**
Figure S1. HPLC Trace for Compound **28**	S2
Figure S2. HPLC Trace for Compound **42**	S2
Figure S3. HPLC Trace for Compound **45**	S3
Figure S4. HPLC Trace for Compound **75**	S3
Figure S5. HPLC Trace for Compound **81**	S4
Figure S6. ^{1}H-NMR Spectra for Compound **28**	S5
Figure S7. ^{13}C-NMR Spectra for Compound **28**	S6
Figure S8. ^{1}H-NMR Spectra for Compound **42**	S7
Figure S9. ^{13}C-NMR Spectra for Compound **42**	S8
Figure S10. ^{1}H-NMR Spectra for Compound **45**	S9
Figure S11. ^{13}C-NMR Spectra for Compound **45**	S10
Figure S12. ^{1}H-NMR Spectra for Compound **75**	S11
Figure S13. ^{13}C-NMR Spectra for Compound **75**	S12
Figure S14. ^{1}H-NMR Spectra for Compound **81**	S13
Figure S15. ^{13}C-NMR Spectra for Compound **81**	S14

HPLC Traces for compounds in Figure 5 and Table 6.

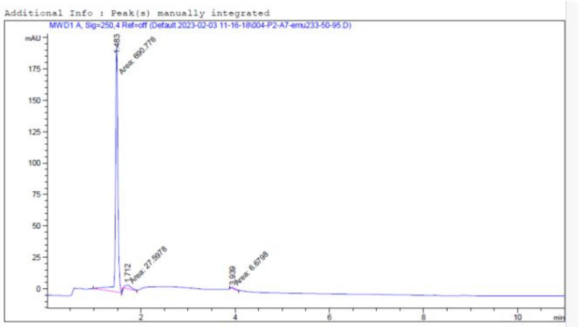

Signal 1: MWD1 A, Sig=250,4 Ref=off

Peak #	RetTime [min]	Type	Width [min]	Area [mAU*s]	Height [mAU]	Area %
1	1.483	MM	0.0582	690.77576	197.66954	95.2724
2	1.712	MM	0.1597	27.59784	2.87989	3.8063
3	3.939	MM	0.1199	6.67980	9.28366e-1	0.9213
Totals :				725.05339	201.47780	

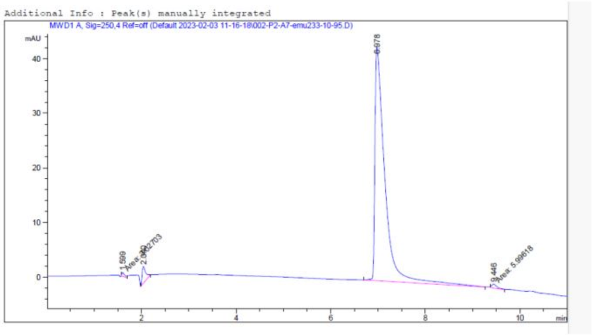

Signal 1: MWD1 A, Sig=250,4 Ref=off

Peak #	RetTime [min]	Type	Width [min]	Area [mAU*s]	Height [mAU]	Area %
1	1.599	MM	0.0750	3.02703	6.72752e-1	0.4694
2	2.040	BB	0.0771	15.91988	3.03976	2.4688
3	6.978	BB	0.2048	619.90735	42.84700	96.1320
4	9.446	MM	0.1327	5.99618	7.53162e-1	0.9299
Totals :				644.85043	47.31268	

Figure S1. HPLC Traces for Compound **28**.

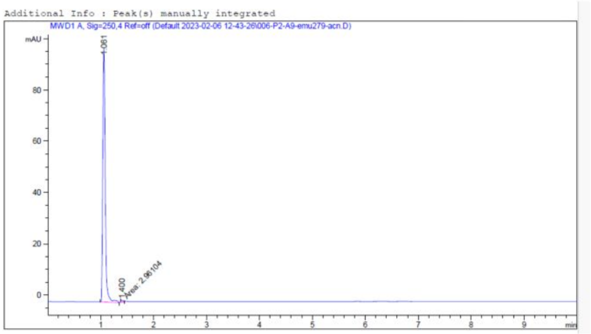

Signal 1: MWD1 A, Sig=250,4 Ref=off

Peak #	RetTime [min]	Type	Width [min]	Area [mAU*s]	Height [mAU]	Area %
1	1.061	BV R	0.0491	315.61221	99.17891	99.0705
2	1.400	MM	0.0616	2.96104	8.01303e-1	0.9295
Totals :				318.57325	99.98021	

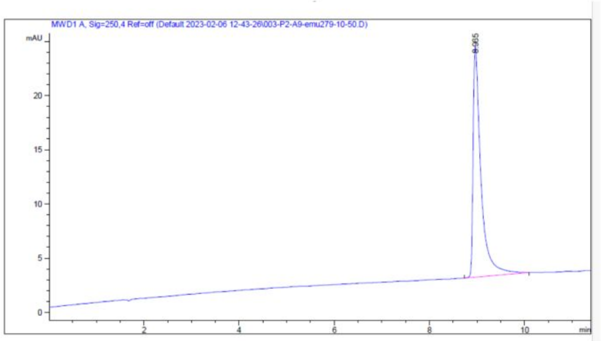

Peak #	RetTime [min]	Type	Width [min]	Area [mAU*s]	Height [mAU]	Area %
1	8.965	BB	0.1641	245.24875	21.34171	100.0000
Totals :				245.24875	21.34171	

Figure S2. HPLC Traces for Compound **42**.

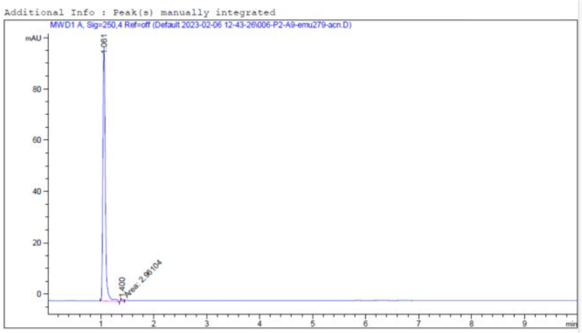

Signal 1: MWD1 A, Sig=250,4 Ref=off

Peak #	RetTime [min]	Type	Width [min]	Area [mAU*s]	Height [mAU]	Area %
1	1.061	BV R	0.0491	315.61221	99.17891	99.0705
2	1.400	MM	0.0616	2.96104	8.01303e-1	0.9295
Totals :				318.57325	99.98021	

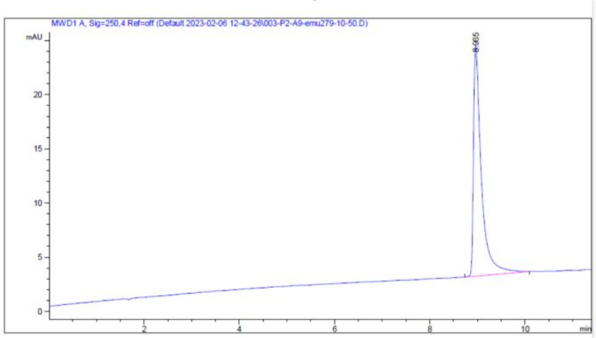

Peak #	RetTime [min]	Type	Width [min]	Area [mAU*s]	Height [mAU]	Area %
1	8.965	BB	0.1641	245.24875	21.34171	100.0000
Totals :				245.24875	21.34171	

Figure S3. HPLC Traces for Compound **45**.

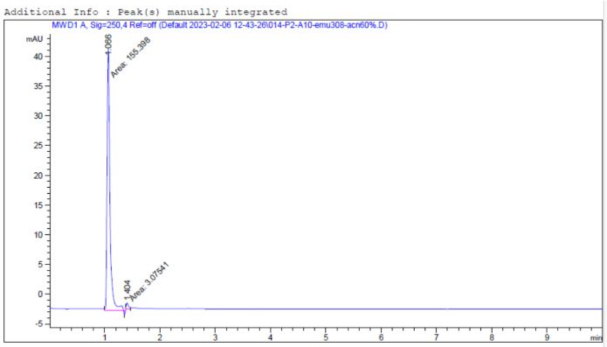

Signal 1: MWD1 A, Sig=250,4 Ref=off

Peak #	RetTime [min]	Type	Width [min]	Area [mAU*s]	Height [mAU]	Area %
1	1.066	MM	0.0584	155.39781	44.33251	98.0594
2	1.404	MM	0.0477	3.07541	1.07504	1.9406
Totals :				158.47322	45.40755	

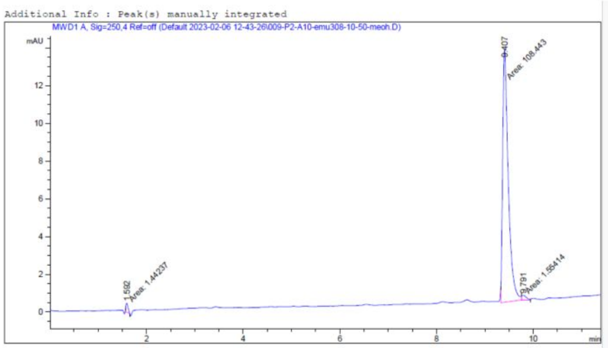

Signal 1: MWD1 A, Sig=250,4 Ref=off

Peak #	RetTime [min]	Type	Width [min]	Area [mAU*s]	Height [mAU]	Area %
1	1.592	MM	0.0511	1.44237	4.70730e-1	1.2943
2	9.407	MM	0.1348	108.44324	13.40413	97.3111
3	9.791	MM	0.1069	1.55414	2.42234e-1	1.3946
Totals :				111.43975	14.11710	

Figure S4. HPLC Traces for Compound **75**.

Additional Info : Peak(s) manually integrated

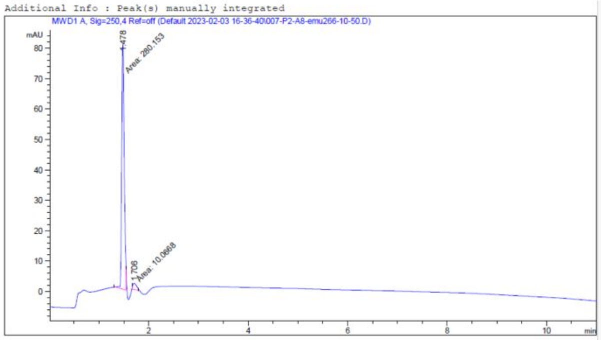

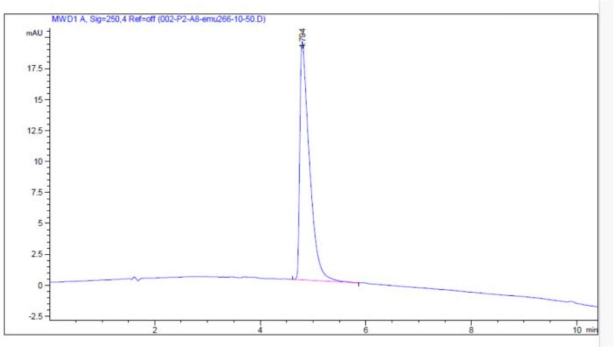

Signal 1: MWD1 A, Sig=250,4 Ref=off

Peak #	RetTime [min]	Type	Width [min]	Area [mAU*s]	Height [mAU]	Area %
1	1.478	MM	0.0575	280.15338	81.15968	96.5313
2	1.706	MM	0.0828	10.06684	2.02593	3.4687
Totals :				290.22022	83.18561	

Signal 1: MWD1 A, Sig=250,4 Ref=off

Peak #	RetTime [min]	Type	Width [min]	Area [mAU*s]	Height [mAU]	Area %
1	4.794	BB	0.1816	243.96735	19.28520	100.0000
Totals :				243.96735	19.28520	

Figure S5. HPLC Traces for Compound **81**.

^{1}H-NMR and ^{13}C-NMR spectra for compounds in Figure 5 and Table 6.

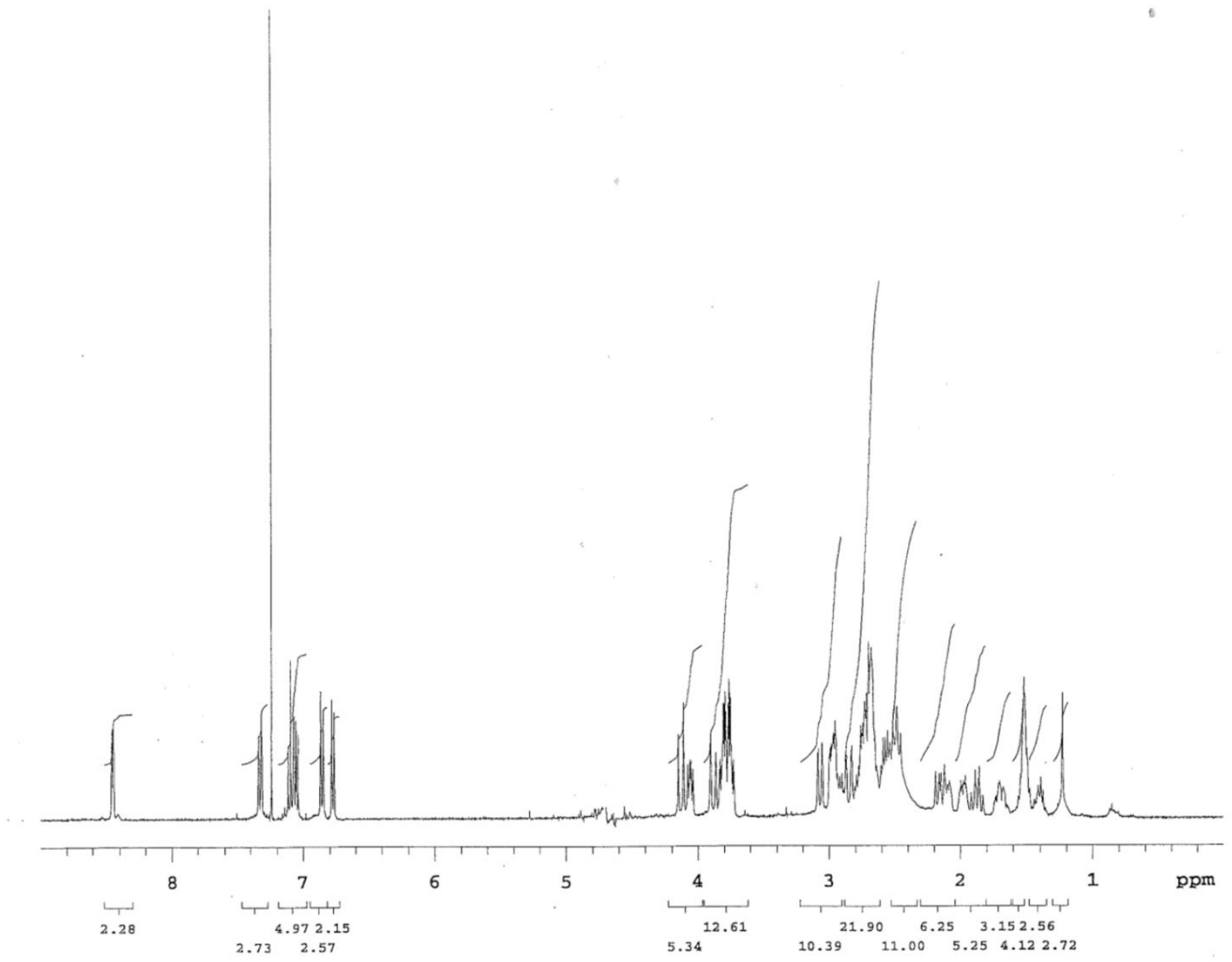

Figure S6. ^{1}H-NMR Spectra for Compound **28**.

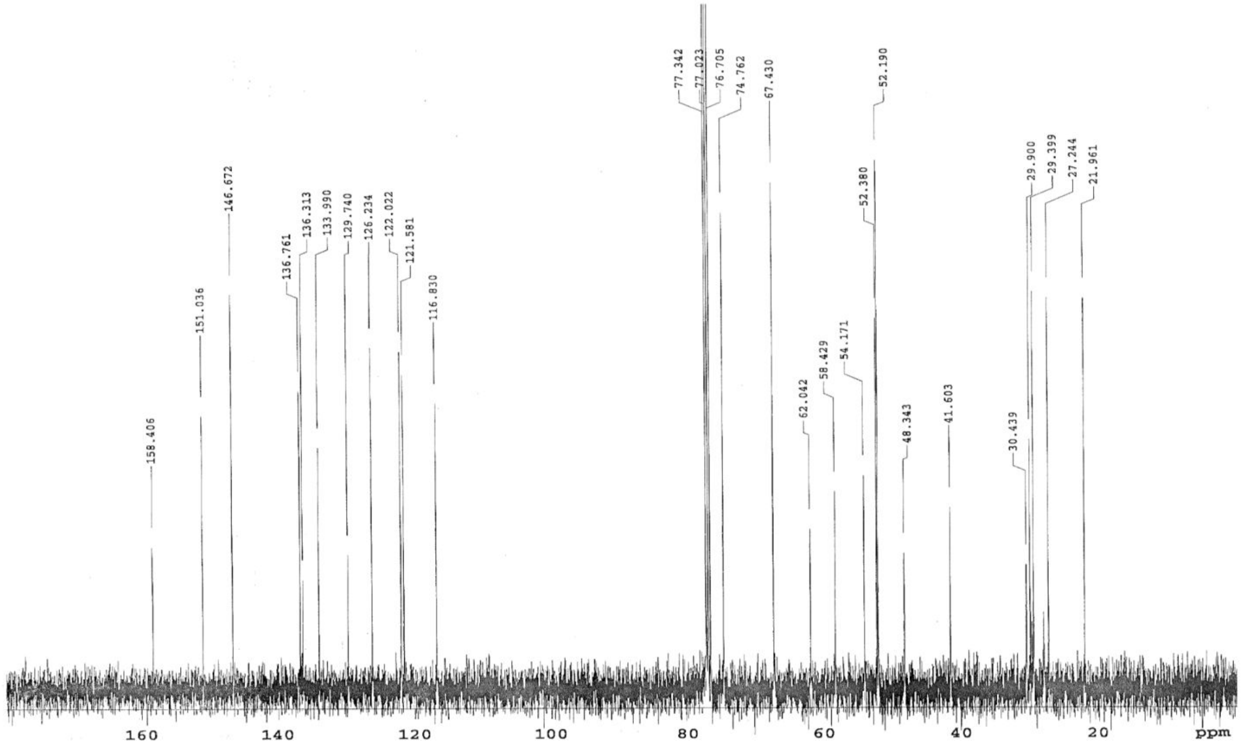

Figure S7. ^{13}C-NMR Spectra for Compound **28**.

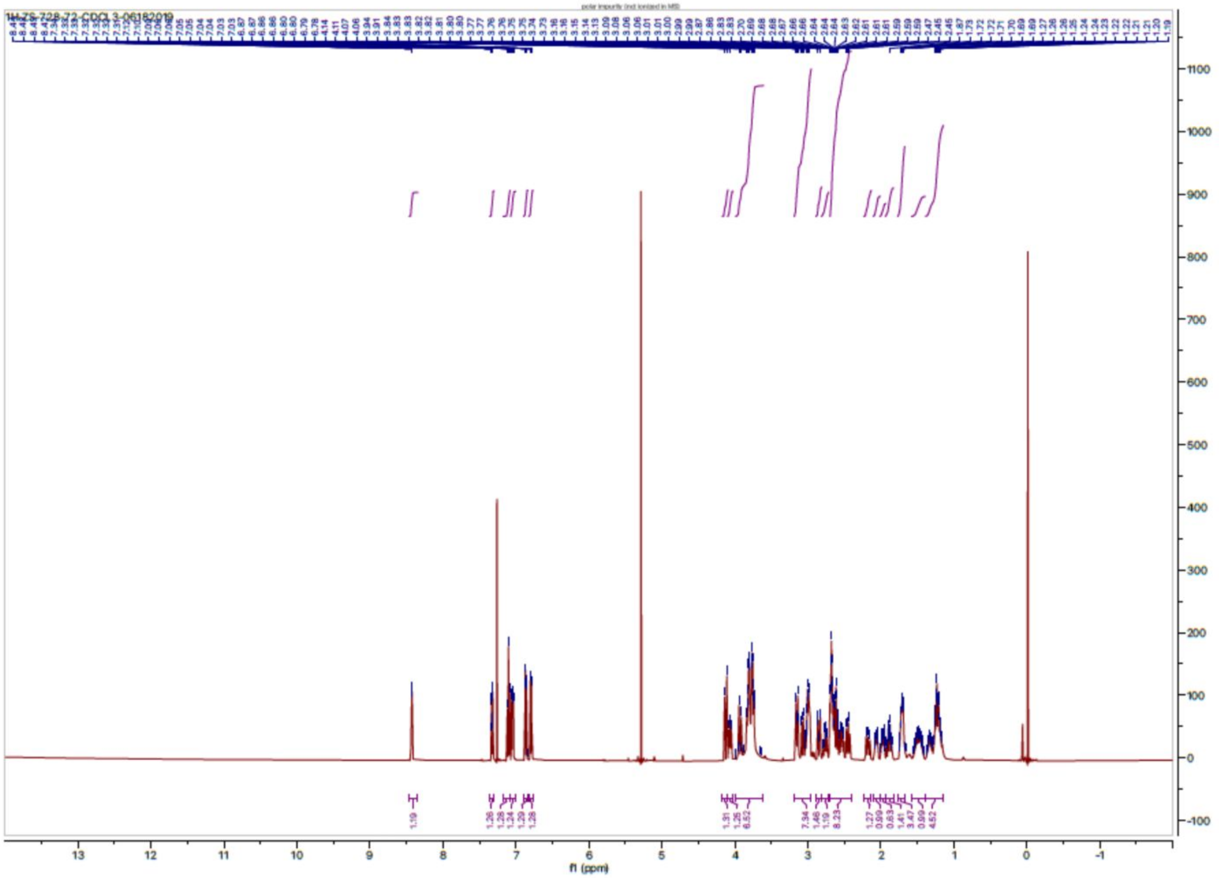

Figure S8. ^{1}H-NMR Spectra for Compound **42**.

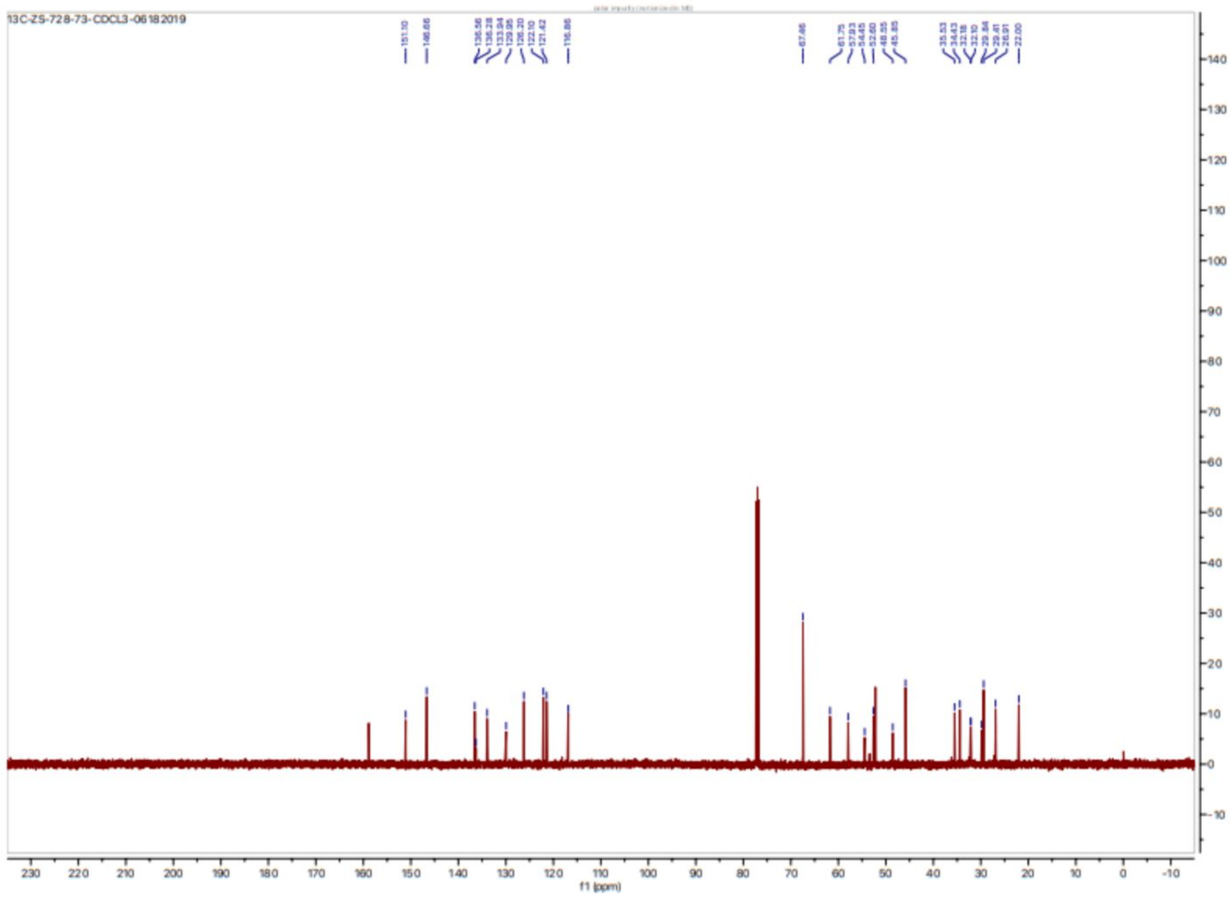

Figure S9. ^{13}C-NMR Spectra for Compound **42**.

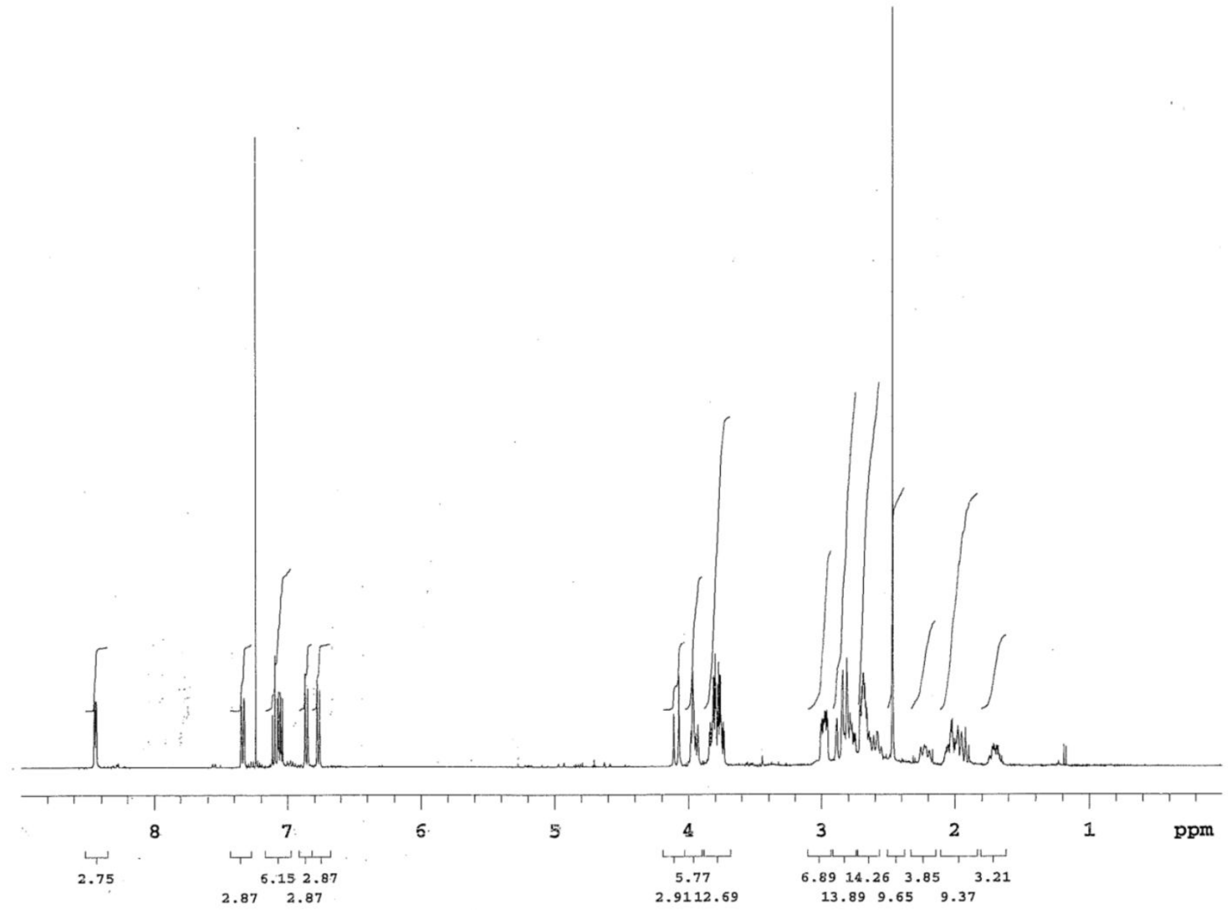

Figure S10. ^{1}H-NMR Spectra for Compound **45**.

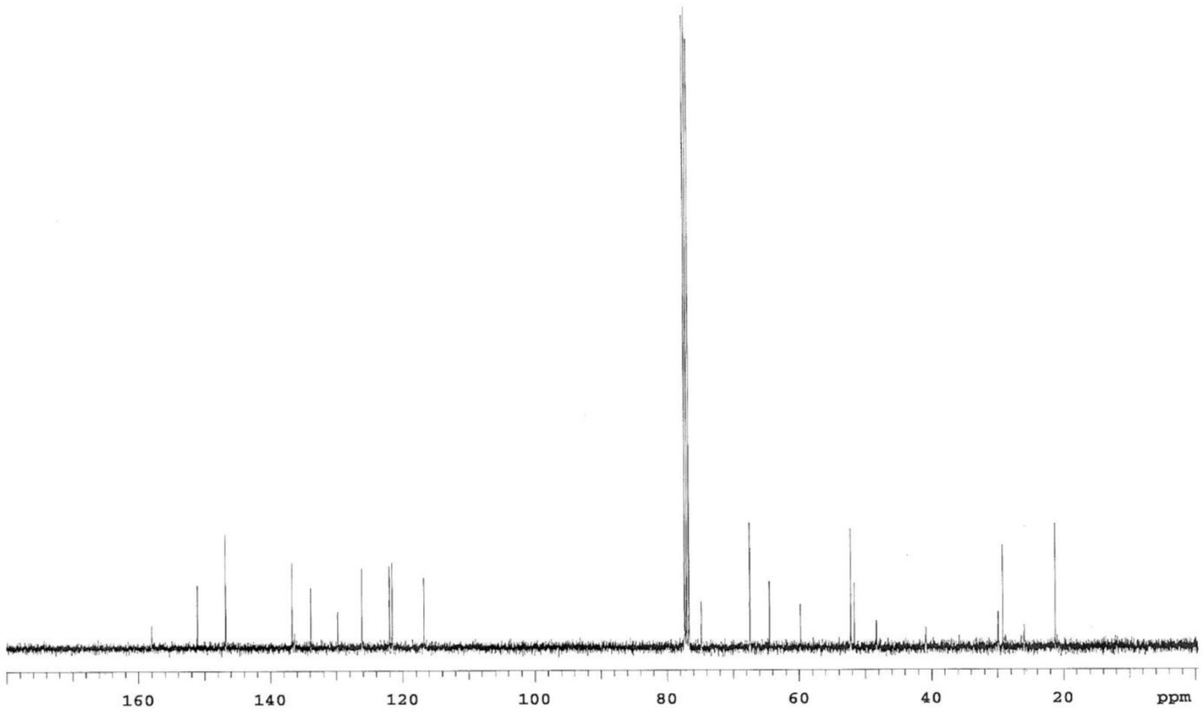

Figure S11. ^{13}C-NMR Spectra for Compound **45**.

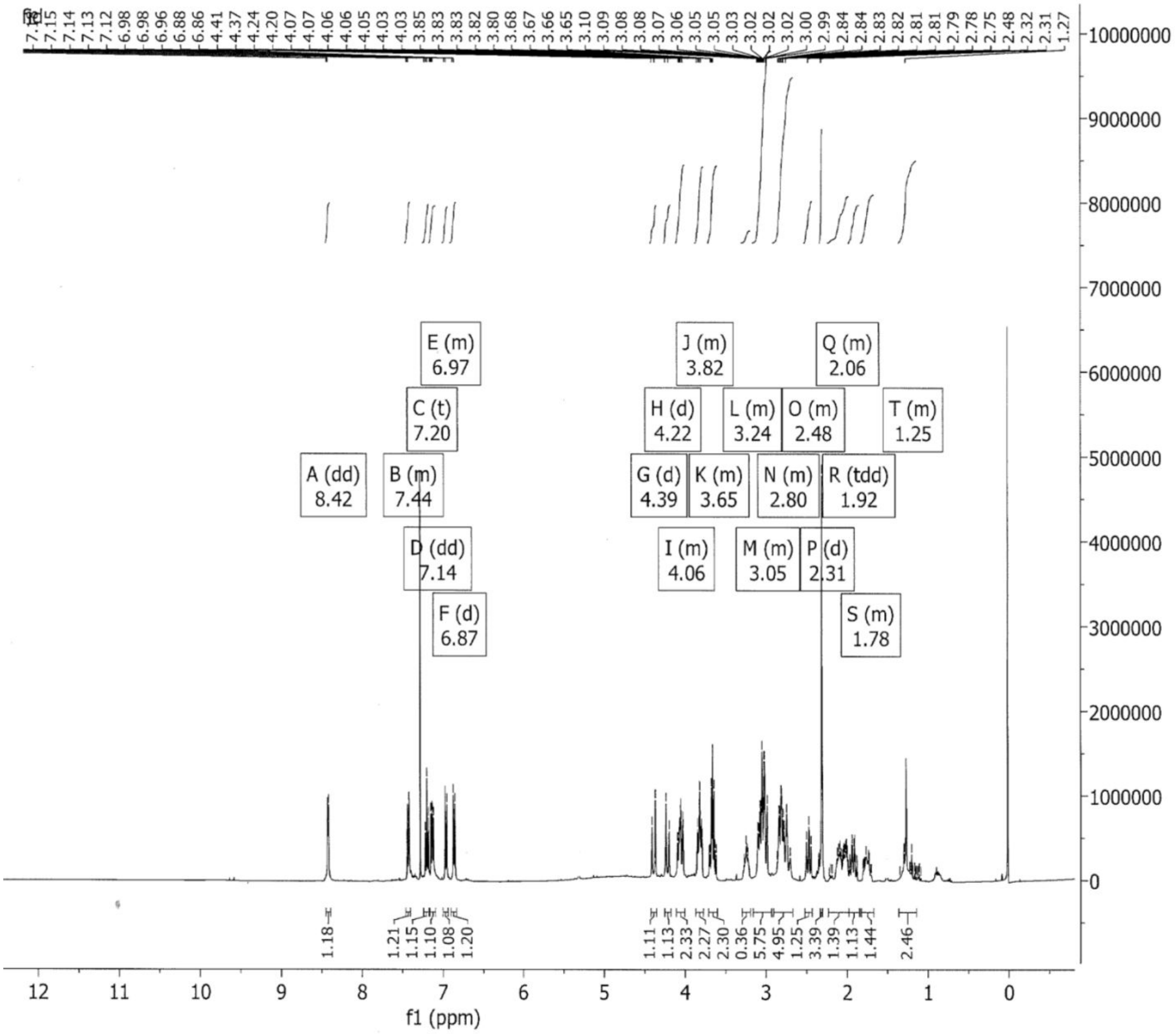

Figure S12. ^{1}H-NMR Spectra for Compound **75**.

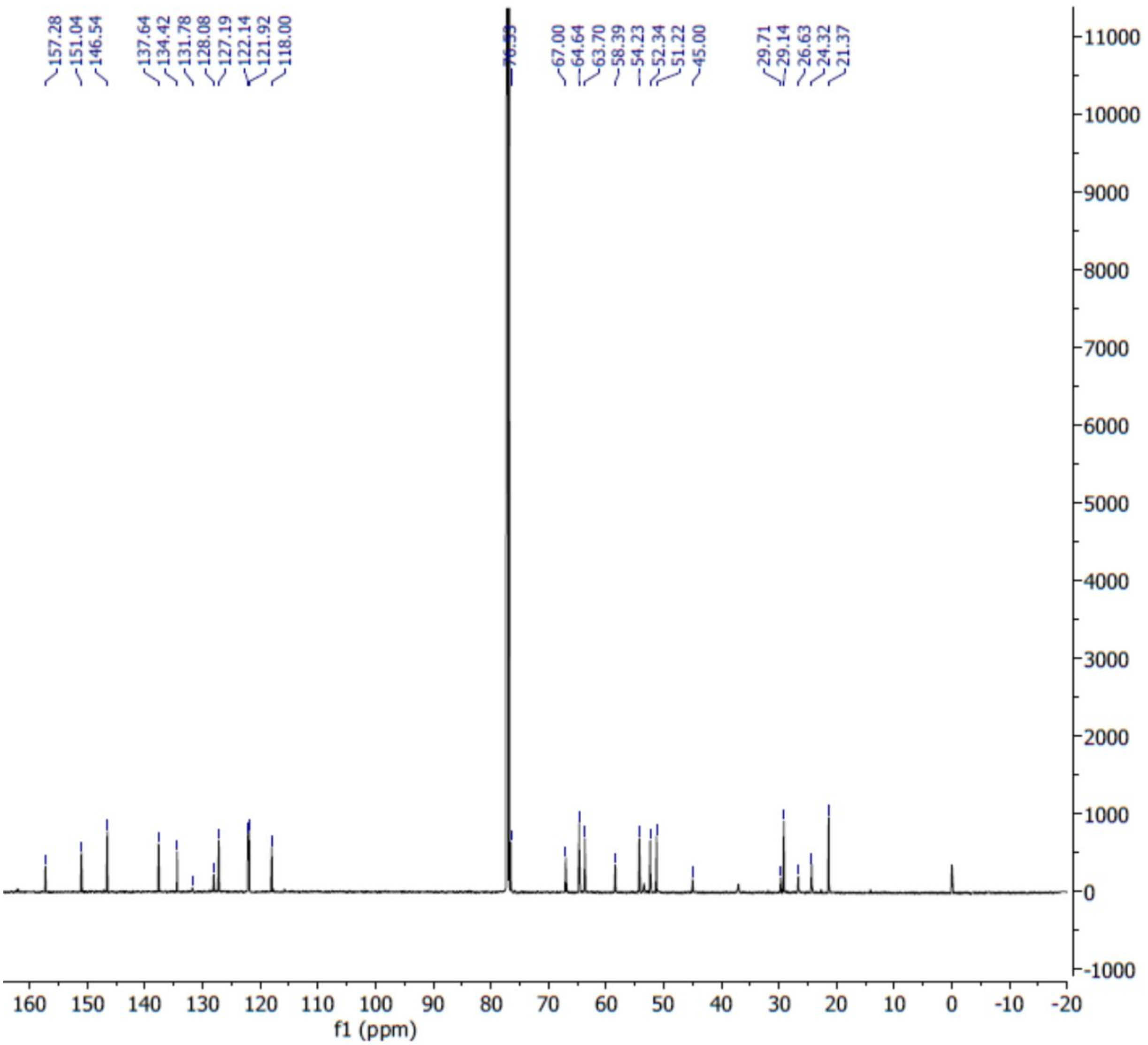

Figure S13. ^{13}C-NMR Spectra for Compound **75**.

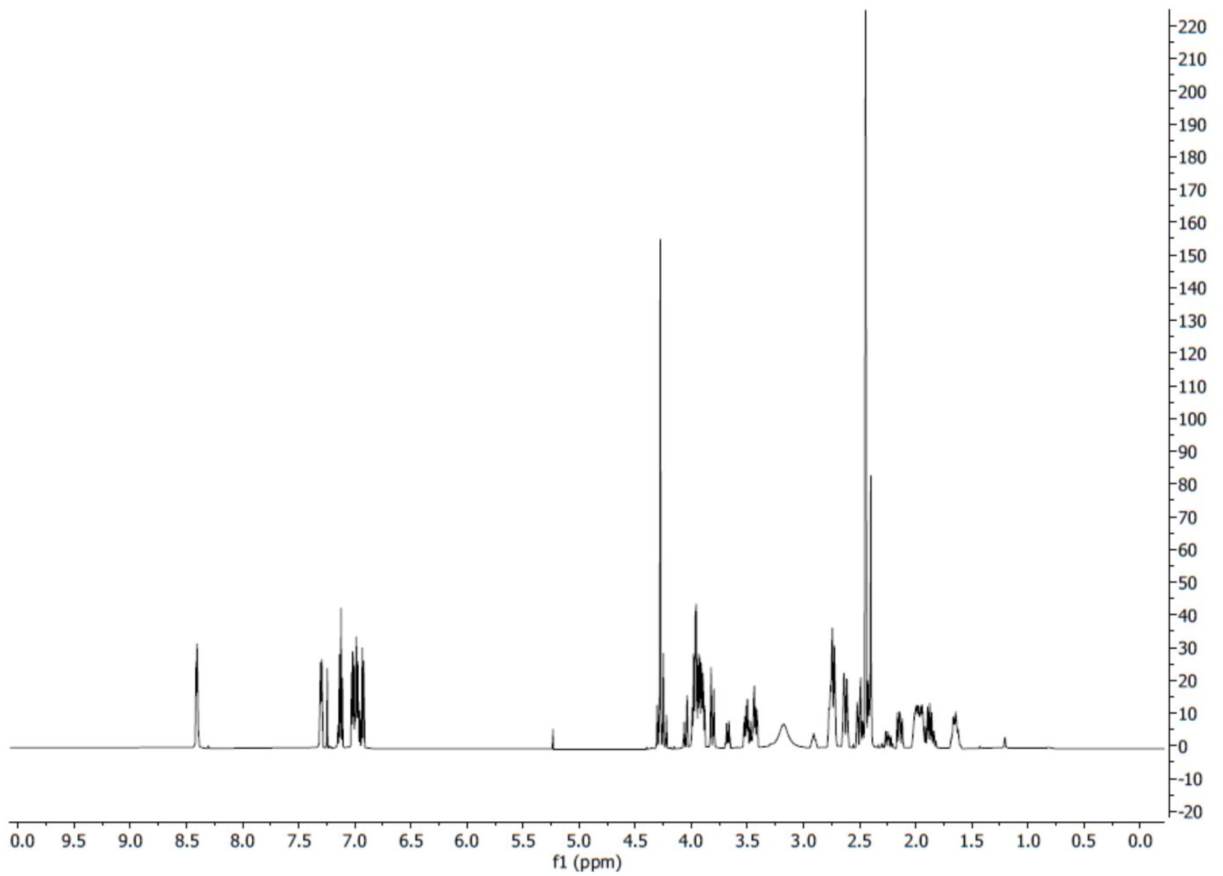

Figure S14. ^{1}H-NMR Spectra for Compound **81**.

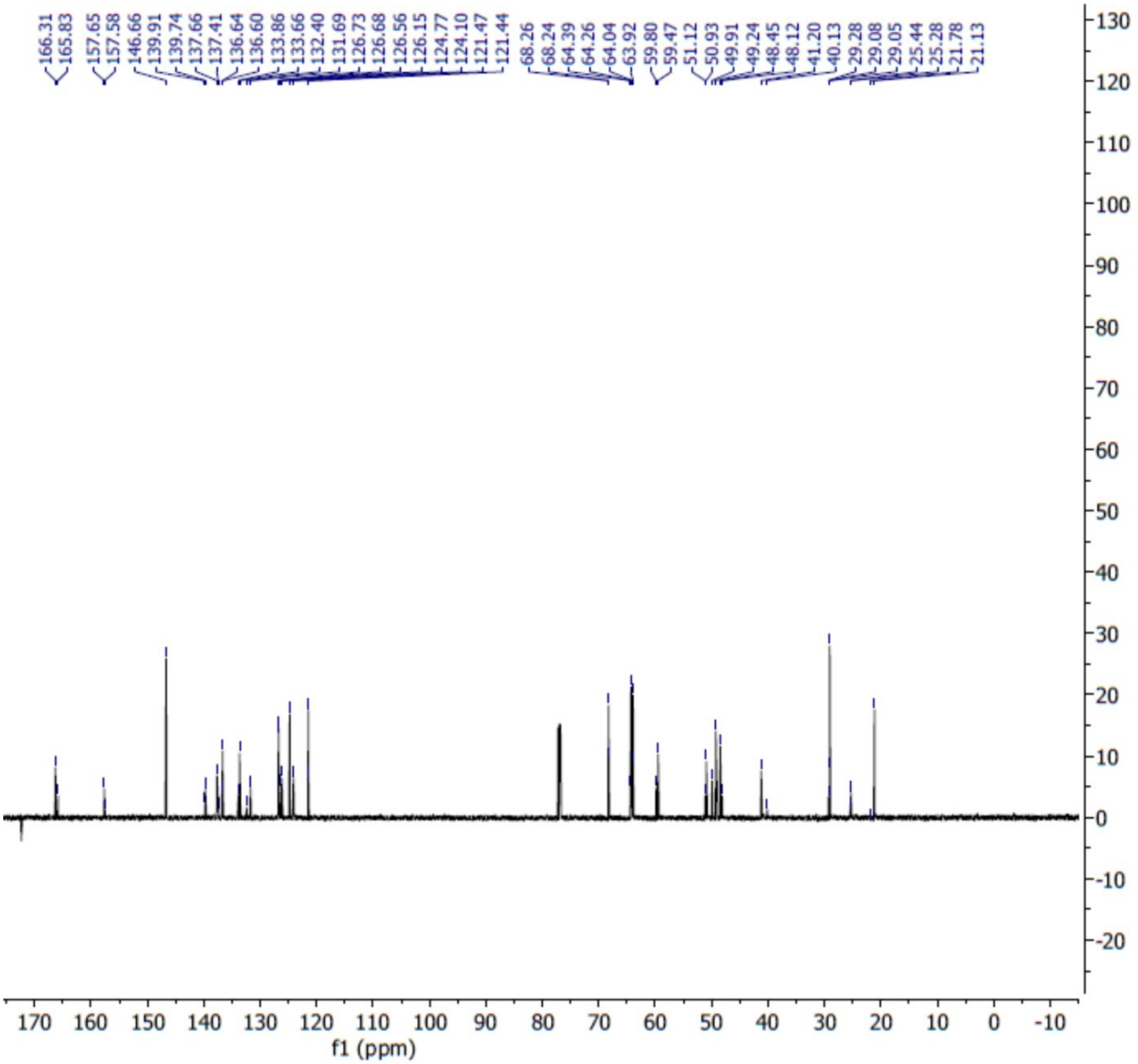

Figure S15. ^{13}C-NMR Spectra for Compound **81**.
